# Systematic characterization of the branch point binding protein, splicing factor 1, gene family in plant development and stress responses

**DOI:** 10.1186/s12870-020-02570-6

**Published:** 2020-08-18

**Authors:** Kai-Lu Zhang, Zhen Feng, Jing-Fang Yang, Feng Yang, Tian Yuan, Di Zhang, Ge-Fei Hao, Yan-Ming Fang, Jianhua Zhang, Caie Wu, Mo-Xian Chen, Fu-Yuan Zhu

**Affiliations:** 1 Co-Innovation Center for Sustainable Forestry in Southern China, College of Biology and the Environment, Nanjing Forestry University, Nanjing, 210037 Jiangsu Province China; 2 College of Light Industry and Food Engineering, Nanjing Forestry University, Nanjing, 210037 Jiangsu Province China; 3 Key Laboratory of Pesticide & Chemical Biology, Ministry of Education, College of Chemistry, Central China Normal University, Wuhan, 430079 China; 4 Shenzhen Research Institute, The Chinese University of Hong Kong, Shenzhen, China; 5 Department of Biology, Hong Kong Baptist University, and State Key Laboratory of Agrobiotechnology, The Chinese University of Hong Kong, Shatin, Hong Kong; 6 Shenzhen Institute of Synthetic Biology, Shenzhen Institutes of Advanced Technology, Chinese Academy of Sciences, Shenzhen, 518055 PR China

**Keywords:** Alternative splicing, Expression profile, Phylogenetics, Plants, Promoter, Splicing factor

## Abstract

**Background:**

Among eukaryotic organisms, alternative splicing is an important process that can generate multiple transcripts from one same precursor messenger RNA, which greatly increase transcriptome and proteome diversity. This process is carried out by a super-protein complex defined as the spliceosome. Specifically, splicing factor 1/branchpoint binding protein (SF1/BBP) is a single protein that can bind to the intronic branchpoint sequence (BPS), connecting the 5′ and 3′ splice site binding complexes during early spliceosome assembly. The molecular function of this protein has been extensively investigated in yeast, metazoa and mammals. However, its counterpart in plants has been seldomly reported.

**Results:**

To this end, we conducted a systematic characterization of the *SF1* gene family across plant lineages. In this work, a total of 92 sequences from 59 plant species were identified. Phylogenetic relationships of these sequences were constructed, and subsequent bioinformatic analysis suggested that this family likely originated from an ancient gene transposition duplication event. Most plant species were shown to maintain a single copy of this gene. Furthermore, an additional RNA binding motif (RRM) existed in most members of this gene family in comparison to their animal and yeast counterparts, indicating that their potential role was preserved in the plant lineage.

**Conclusion:**

Our analysis presents general features of the gene and protein structure of this splicing factor family and will provide fundamental information for further functional studies in plants.

## Background

In eukaryotes, canonical splicing removes noncoding intronic sequences and assembles the coding elements into mature mRNAs while alternative splicing (AS) generates different multiple transcripts that encode proteins with distinct structures and functions by differential usage of exons or splice site [58, 70]. The resulting transcripts of AS greatly contribute to post-transcriptional regulation, biological complexity and proteome diversity in eukaryotes [20, 50, 74]. Given that on average there are approximately 8 exons in each transcript in the human transcriptome and the degenerative nature of corresponding splice sites [20], pre-mRNA splicing is sophistically catalysed by the spliceosome. Spliceosome is a multi-megadalton protein complex, which consists of five (U1, U2, U4, U5 and U6) small nuclear ribonucleoprotein particles (snRNPs) and over 100 spliceosomal proteins [74]. Furthermore, the early assembly of spliceosome complex E or the commitment complex is an ATP-independent process and contains U1 snRNPs, SF1 and U2 snRNP auxiliary factors (U2AF large and U2AF small subunits) [48, 51]. Subsequently, the pre-spliceosome complex A is formed by replacing SF1 with SF3b155/SAP155 of U2 snRNPs [19, 67, 77]. Stepwise assembly of the following spliceosome during the splicing reaction has been reported as well [44, 63]; however, splice site recognition is a critical step during early assembly of the spliceosome. The current model describes the binding of U1 snRNP and U1 snRNA to a short stretch of 6 nucleotides at the 5′ splice site, of splicing factor 1 (SF1)/mammalian branch point binding protein (mBBP) at the branch point, and of U2 snRNP auxiliary factors at the 3′ splice site [46]. These three *cis*-elements are necessary but usually insufficient to define a specific exon–intron boundary. Thus, additional splicing enhancers or silencers located at exons and introns may allow the recognition of genuine splice sites during early spliceosome assembly [29].

Importantly, SF1 preferentially binds to the intron branch point sequence (BPS) which is adjacent to the binding site (polypyrimidine tract, Py) of U2AF large subunits (mammal U2AF65 and fission yeast U2AF59), bridging U1 and U2AF to form an intermediate lariat structure [58, 81]. In particular, SF1 is characterized by the presence of two types of RNA binding motifs at the N-terminus, a K homology/Quaking 2 (KH/QUA2) domain which originated from the human heterogeneous ribonucleoprotein (hnRNP) K protein [17, 66] and one or two zinc knuckle motif(s) (CX_2_CX_4_HX_4_C, X represents any amino acid). SF1 also contains a proline-rich region at C-terminus [2, 3]. Intriguingly, the yeast KH domain specifically binds to the BPS of pre-mRNAs with a Gly-Pro-Arg-Gly motif and the variable loop of the KH domain [39] and is necessary for spliceosome assembly [57]. The first but not the second zinc knuckle domain in yeast has been demonstrated to bind RNA with high affinity [16]. Moreover, the stability of the SF1–U2AF65–RNA complex is further affected by the phosphorylation status of several SF1 serine residues (Ser20, Ser80 and Ser82) in vitro [45]. The proline-rich region of SF1 interacts with U1 snRNP Prp40/FBP11 in yeast and human [2, 38]. In regards to its interaction partner, the U2AF large subunit, the N-terminal of SF1 interacts with its non-canonical RNA recognition motifs (RRM) or U2AF homology motif (UHM) [57, 62], whereas the other two RRMs of U2AF large subunit bind to the Py region [65].

A previous study in fission yeast (*Schizosaccharomyces pombe*) suggests that the initial co-recognition of the branch site and 3′ splice site is pivotal for correct splicing of target pre-mRNAs [60]. Because of the importance of splice site recognition for gene expression and protein diversity, SF1 has been demonstrated to play essential roles in a number of eukaryotic species including human (*Homo sapiens*), mice (*Mus musculus*), budding yeast (*Saccharomyces cerevisiae*), common fruit fly (*Drosophila melanogaster*) and roundworm (*Caenorhabditis elegans*) [2, 27, 47, 64, 68]. For example, in humans, missense mutation of splicing factors which are responsible for splice site recognition, such as SF1, has been linked to tumourigenesis [33]. Similarly, heterozygous SF1 (+/−) knockdown mice are susceptible to colon tumourigenesis induced by an organotrophic carcinogen, azoxymethane [64], and SF1 has been found to associate with beta-catenin/TCF4 complex, suggesting its role in carcinogenesis [49]. In contrast, knockdown of SF1 suppresses the development of germ cell tumours in mice [83], indicating its tissue dependency in cancer research. Furthermore, the molecular function of SF1 has been extensively studied in yeast. For instance, a sf1 mutant strain causes frequent exon skipping in fission yeast [52]. Additionally, SF1 has been proposed to recognize sub-optimal sequences in specific introns and lead to nuclear accumulation of pre-mRNA with aberrant splicing [73]. However, increasing evidence indicates that this protein is a regulator of splice site recognition and does not reduce general splicing, specifically during alternative splicing by targeting a subset of genes [46, 52, 68]. This hypothesis is supported by the fact that knockdown of SF1 in both yeast and human extracts only slightly affects the splicing outcome [22]. RNAi targeting of this gene has been demonstrated to not affect the splicing pattern of several splicing marker genes tested [68].

In comparison to studies in human and yeast, few reports have been published related to plant *SF1* genes. Similar functions of the *Arabidopsis SF1* gene were proposed in an early study in 2014 [30]. This plant SF1 homologue is reportedly responsible for the splicing of a group of transcripts. The loss-of-function mutant (*atsf1–2*) of this gene leads to abnormal development (early flowering and dwarfism) and ABA or heat stress sensitivity in *Arabidopsis* [30, 36]. Subsequently, the domain structure and its functional relationships have been substantially investigated [36], and the RRM domain is considered crucial to maintain its function in plants. Moreover, SF1 may have a different mechanism of 3′ splice site recognition in plant because the plant SF1 homologs contain a different RRM domain compared with fungal and metazoan counterparts [53, 78]. On the other hand, a study found that AtSF1 may be likely to play a functional role in the cytoplasm because it was found to shuttle between the nucleus and cytoplasm [54]. However, no related investigations have been conducted on the phylogenetic analysis of plant *SF1* genes and their regulatory mechanisms. Although it is a highly conserved family and has conserved functions in eukaryotes, plant *SF1* genes may have overlapping and distinct roles compared to the mammalian genes. Hence, studying the phylogenetic relationship and regulatory mechanism of plant *SF1* genes may make us understand the evolutionary history, characteristics an expression profile of this gene family and predict specific functions in plants. This can lay the foundation for further functional studies in Viridiplantae. To this end, we systematically identified 92 *SF1* sequences from 59 plant species, ranging from algae to higher plants. Meanwhile, the gene and protein structure, potential regulation at promoter regions and expression pattern of these genes were further investigated. In this study, we hypothesize that plant SF1 is structurally different from its counterparts in animals and yeast, but it is conserved among lower and higher plants, indicating its specific role in alternative splicing in branch point recognition.

## Methods

### Sequence acquisition and identification of plant *SF1* genes

The *Arabidopsis thaliana SF1* protein sequence (AT5G51300) was used to search similar sequences in all available plant species from the Phytozome v12.1 database (https://phytozome.jgi.doe.gov/pz/portal.html) [18] by running the BLASTp program with an e-value cutoff = 1e-^10^ (the other parameters were the default settings) [7]. Then, the retrieved protein sequences were examined and filtered using the HMMER score (default settings) [31], which contained PF16275 (Splicing factor 1 helix-hairpin domain, SF1-HH), PF00013 (K Homology domain, KH_1) and PF00076 (RNA recognition motif, RRM_1). Finally, 92 putative *SF1* sequences from 59 plant species were identified. Detailed information including groups, plant species, common names and number of SF1 homologs reported for each plant species for subsequent analysis are listed in Table S1. Subcellular location prediction of identified SF1 proteins was carried out using WoLF PSORT (https://wolfpsort.hgc.jp/) [25].

### Construction of molecular phylogenetic tree of plant *SF1* genes

Protein sequences of the aforesaid plant *SF1* genes were extracted from Phytozome v12.1 database for phylogenetic relationship analysis. The sequences with the longest coding sequences were chosen for genes with multiple different splicing isoforms. Then, multiple *SF1* protein sequences were aligned with the Muscle v3.8 software with default settings [13]. The molecular phylogenetic tree of plant *SF1* genes was then constructed using the maximum likelihood method (ML, JTT + G + I model) via PhyML v3.0 program with the following parameters: initial tree: BioNJ; discrete gamma model: yes; number of categories: 4; gamma shape parameter: 0.709; proportion of invariant: 0.021 subtree patterns aliasing: no [21]. FigTree v1.4.3 was used to visualize and edit the phylogenetic tree.

### Gene structure, protein domain and multiple Em for motif elicitation (MEME) analysis

Required genomic, cDNA, and peptide sequences and all *SF1* gene structures were downloaded from the Phytozome v12.1 database. Corresponding intron phases were generated using the online program Gene Structure Display Server 2.0 (GSDS2.0) (http://gsds.cbi.pku.edu.cn) [26]. Correlation analysis of *SF1* exons were performed by using the piece2 webserver (http://www.bioinfogenome.net/piece/search.php? tdsourcetag=s_pctim_aiomsg) [76]. *SF1* protein sequences were used to search for matching Pfam families using the HMMER website (https://www.ebi.ac.uk/Tools/hmmer/) [14]. Then, protein domain patterns were drawn by using TBtools software [8] according to the full Pfam resultant table. Conserved motifs of plant *SF1* cDNA sequences and protein sequences were analysed on the MEME online program (http://meme-suite.org/tools/meme) [5] considering a maximum of the 10 most preserved motifs predicted for each sequence and leaving other settings on the default parameters.

### Motif prediction in promoter regions of plant *SF1* genes

The 1.5-kb 5′-flanking sequences of plant *SF1* genes were extracted from genomic data available in Phytozome database. Prediction of plant putative cis-elements was performed with the online server PlantCARE (http://bioinformatics.psb.ugent.be/webtools/plantcare/html/) [37]. Motifs related to tissue-specific expression, internal hormones and external environmental stress response were selected for further analysis and discussion.

### Expression analysis base on microarray datasets and gene expression experiments

Expression data of *Arabidopsis*, *S. tuberosum*, *G. max, S. lycopersicum, P. trichocarpa* and *B. distachyon*, including tissue specificity and stress responses, were extracted from the eFP browser series of the Bio-analytic Resource for plant biology (http://bar.utoronto.ca/) [34]. Expression values of selected plant *SF1* genes were log transformed (lg) to generate visualize expression difference heatmaps by using BAR HeatMapper Tool program (http://bar.utoronto.ca/ntools/cgi-bin/ntools_heatmapper.cgi).

### Gene expression experiments

Total RNA of samples from different plant tissues were extracted by RNeasy Mini kit (QIAGEN, USA) and subsequently reversed transcribed into cDNA by FastKing gDNA Dispelling RT SuperMix FastKing (TIANGEN, China) according to the manufacturer’s instruction. RT-PCR amplification were programmed as followings: 95 °C, 3 min; 95 °C, 30 s; 52 °C, 15 s; 72 °C, 45 s; 26/30 cycles; 72 °C 5 min. SYBR Premix Ex TaqTM (Accurate Biotechnology Co., Ltd. Hunan China) was used for quantitative real-time RT-PCR analysis which was conducted on the StepOne Plus real-time PCR system following optimized program: 95 °C, 30 s; 95 °C, 5 s; 60 °C, 30 s; 40 cycles. The data were normalized to the expression of internal reference genes (Table S6) and the transcript abundance was determined by the comparative CT value method [61].

### Analysis of protein-protein interaction network and structural conservation

A protein-protein interaction network was generated by the STRING website (https://string-db.org) [12] with representative protein sequences from *Arabidopsis*. The following basic settings were employed: meaning of network edges, evidence (line colour indicates the type of interaction evidence); and active interaction sources, experiments.

There are three domains in the *Arabidopsis* SF1 protein. The phosphorylation and U2AF65 binding of the N-terminal domain of splicing factor 1 during 3′ splice site recognition of *Homo sapiens* (PDBID: 2M0G, identity: 36%, E-value: 7E-17) was similar to that of the K Homology domain. The structure for recognition of the intron branch site RNA by splicing factor 1 of *Homo sapiens* (PDBID: 1K1G, identity: 47%, E-value: 9E-27) can be used as the template for the splicing factor 1 helix-hairpin domain. Therefore, homology modelling was performed with modeller [43] based on two crystal structures. The amino acid conservation scores were calculated using the ConSurf Web server based on the ML method [4]. Input attributes were the 3D model and multiple sequence alignment (Figure S4). Related figures were created based on Pymol with default settings [79].

### Analysis gene structure evolution with orthologue group of *SF1* genes

Reconstruction of the evolutionary history of the structure of the plant *SF1* family of orthologous genes was carried out by searching AT5G51300.1 in the PIECE 2 sever (http://www.bioinfogenome.net/piece/index.php). This provided an exon-intron display for orthologous genes from gene structure data sets linked to the phylogenetic tree.

## Results

### Sequence identification and phylogenetic analysis of the plant *SF1* gene family

**Fig. 1:**
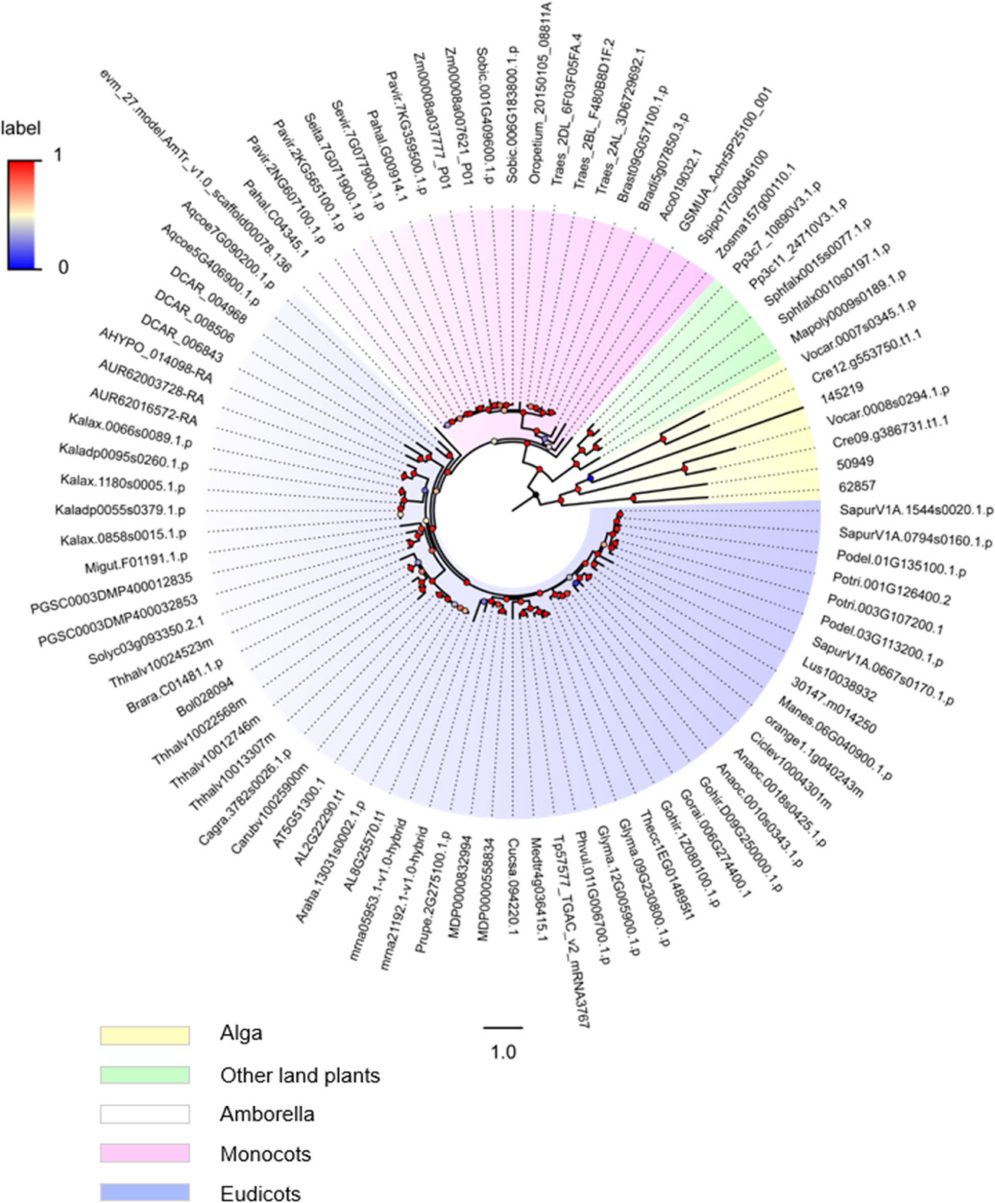
Circular phylogenetic tree of the *SF1* gene family available in plants. The phylogenetic tree of *SF1* genes in plants was constructed based on maximum-likelihood with JTT + G model by using PhyML v3.037. A total of 92 protein sequences from 59 plant species were chosen to calculate the phylogenetic relationship for tree construction. Bootstrap values are labelled at each major branch. The corresponding information of each transcript such as species name, common name, number of identified transcripts and their transcript ID (nomenclature) are shown in Table S1 (taxonomies based on APG-IV system)

**Fig. 2:**
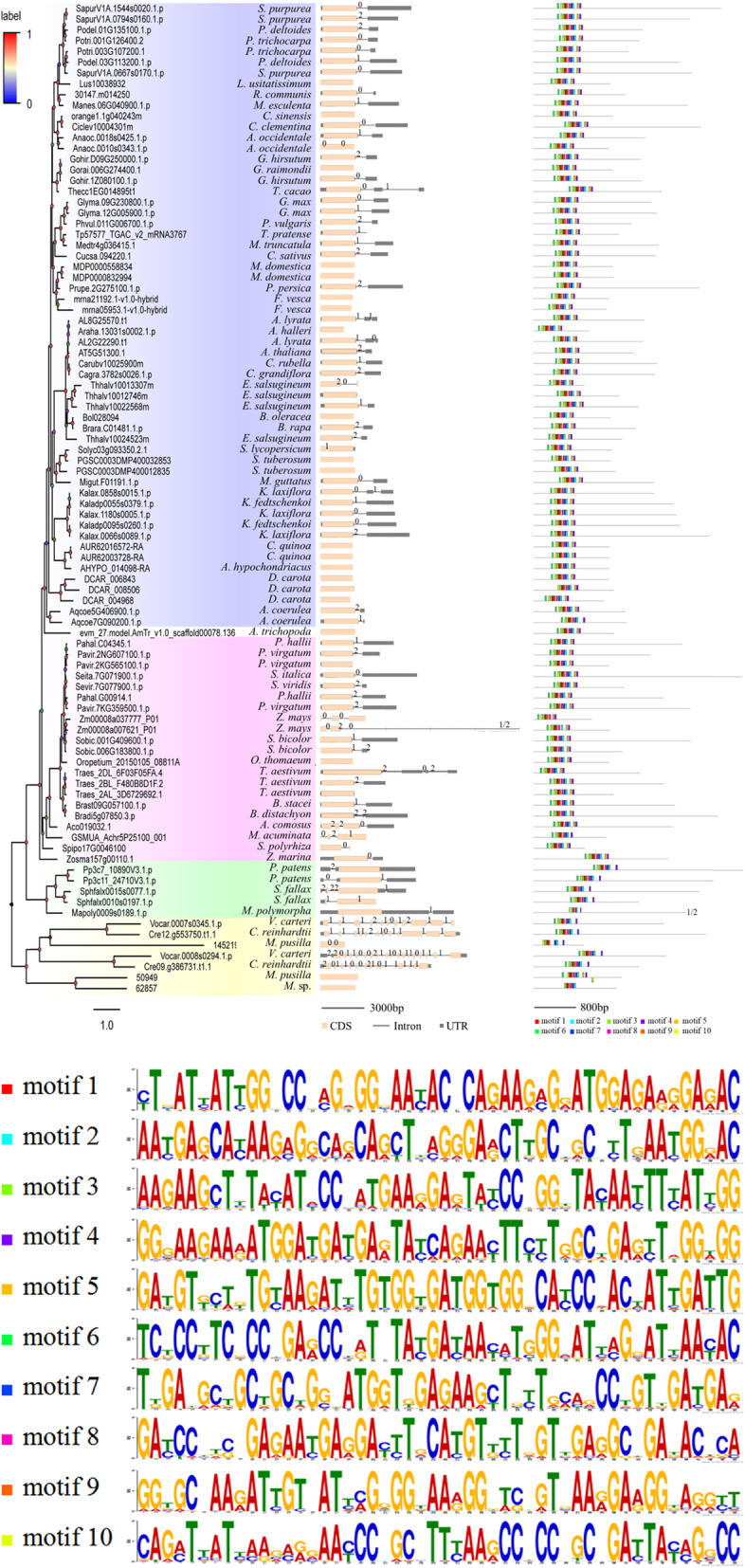
Gene structure comparisons and conserved motif identification among plant *SF1* genes. From left panel to right panel: vertical phylogenetic tree, genomic organization and identified cDNA conserved motifs by MEME analysis. Intron phase 0, 1 and 2 are shown on the gene structure. The conserved sequence of 10 identified motifs represented by different coloured boxes are listed below. Some long genes were reduced to one-half of their original length to fit this picture

To identify *SF1* gene family members in plants, we carried out a BLASTp search using the *Arabidopsis AtSF1* (AT5G51300) amino acid sequence against the Phytozome database (v12.1). After filtering the sequence without *SF1* signature or truncated sequences, a total of 92 sequences from 59 plant species were retrieved, which were roughly classified as 7 algae, 5 bryophyta, 1 basic angiosperm, 21 monocots, and 58 eudicots (Table S1). Specifically, the only species with four copies of plant SF1s was *Eutrema salsugineum* (salt cress) (Table S1). In particular, three copies of *SF1* genes were observed in five species, including *Panicum virgatum* (Switchgrass), *Triticum aestivum* (common wheat), *Daucus carota* (carrot), *Kalanchoe laxiflora* (milky widow’s thrill) and *Salix purpurea* (purple osier willow). Additionally, 20 plant species contained two copies, and 33 species, including the model plant *Arabidopsis*, possessed only one copy of plant *SF1s*, respectively. The relatively larger number of *SF1* genes and higher number of plant species in this work demonstrated the universality and complexity of the *SF1* gene family. The retrieved sequences of 59 plant species provided us with more complete information to analyse the phylogenetic relationship of the *SF1* gene family. Subsequently, a rooted phylogenetic tree was constructed based on the abovementioned 92 protein sequences by using the maximum likelihood method. The tree’s bootstrap (threshold: 0–1) was represented by a colour gradient (Fig. 1). In general, all *SF1* protein sequences were clustered into four major clades including alga (in yellow), other land plants (in green), monocots (in pink) and eudicots (in blue), and one species (*Amborella trichopoda*) belonged to basic angiosperm (shown in colourless). The phylogenetic tree of SF1s (Figs. 1 and 2, left panel) with clear topology and overall high bootstrap values was similar to evolutionary trend from lower plants to higher plants reported in other studies. For example, the genes of algae in the yellow branch were representative members of the lineage that diverged before the evolution of land plants, which was the basal part of the phylogeny. In the blue branch, five sequences from *Kalanchoe* with higher BS values formed a subclade, showing their closer evolutionary relationships. Additionally, Cagra.3782 s0026.1.p from *Capsella grandiflora* and Carubv10025900m from *C. rubella* formed a subclade with the *Arabidopsis* sequences, because they all belong to Brassicaceae, which is consistent with the APG IV system (Fig. 1 and Table S1). Usually, some homologous SF1 sequences from the same species were clustered in the same small branch next to each other; these species included cashew, soybean, apple, woodland strawberry, quinoa, carrot, Colorado blue columbine, maize, common wheat, cereal grass, moss and bog moss (Fig. 1 and Table S1). In contrast, some other homologous SF1 members from the same species were clustered into the different subclades, such as purple osier willow, poplar, eastern cottonwood, salt cress, potato diploid kalanchoe, milky widow’s thrill, hall’s panicgrass, switchgrass, green algae and volvox (Fig. 1 and Table S1).

### Gene structure and conserved motif analysis

**Fig. 3:**
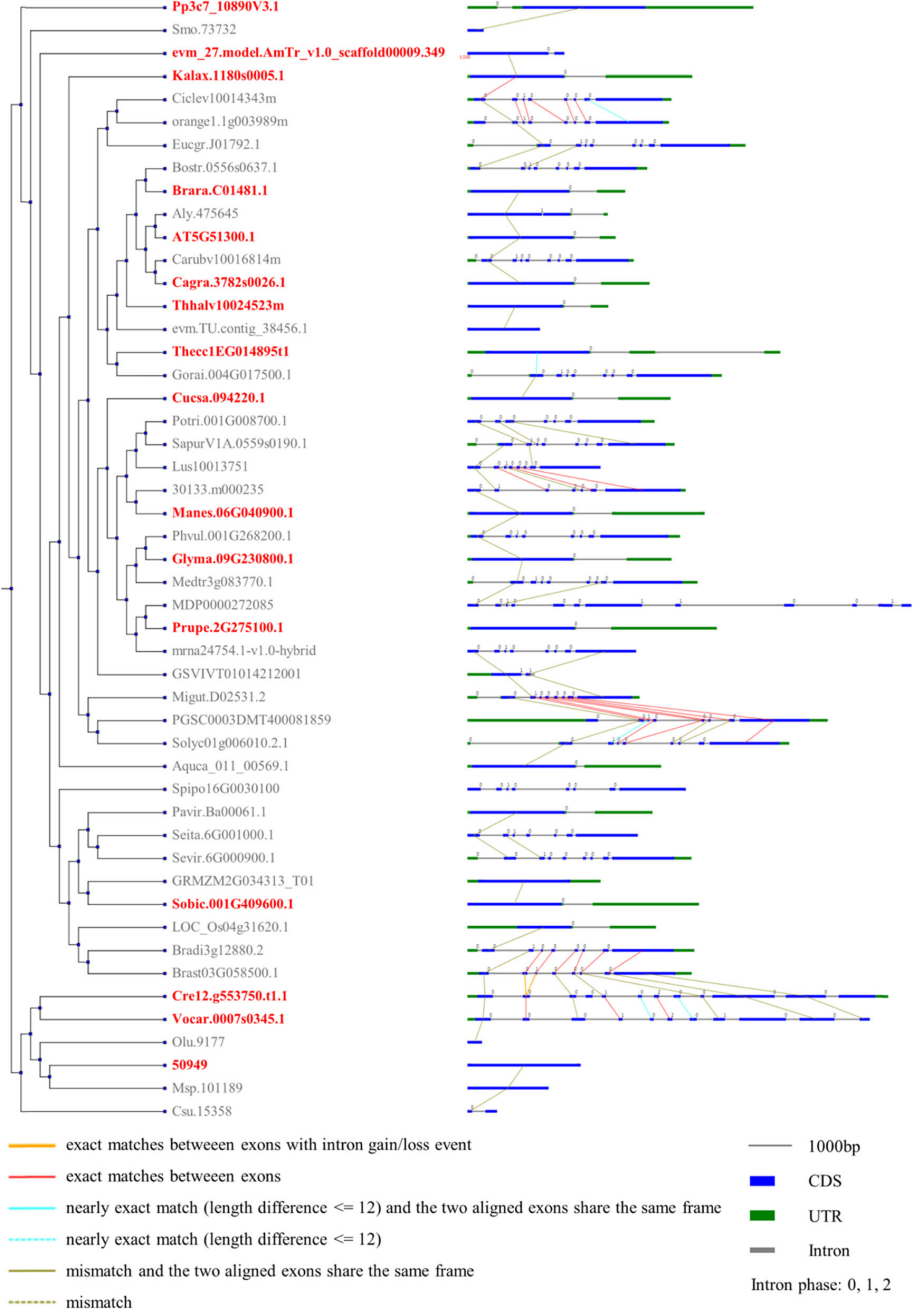
Analysis of gene structure evolution with orthologue group of *SF1* genes. Exon-intron structure and intron phase (right panel) are linked to the plant species tree (left panel). Genes with red colour represent the members of the plant *SF1* genes. Different coloured lines mean different exon comparison results between species

It is necessary to compare the exon-intron organization and conserved motifs of the plant *SF1* gene family to clarify their evolutionary process and potential function. The gene structure models of *SF1* genes were attached to the phylogenetic tree (Fig. 2), and the corresponding intron phase of each was also displayed (Fig. 2, Table S2). Figure 2 (middle, panel) shows that the gene length and structure of each member of the *SF1* family exhibits significant differences. For example, the gene structure of 23 members of 92 *SF1* family genes did not contain intron sequences; this subset accounts for 15.7% of the total number of members. Forty-eight sequences of *SF1* genes had 2 exon-1 intron organizations, accounting for 52.2% of all genes. In particular, some genes from algae had multiple exons, including Vocar.0008 s0294.1.p (*Volvox carteri*) which contained the most exons (19 exons). Moreover, different gene structures were also observed at the same sub-branch. For instance, two sequences from *Zea mays* (maize) (Zm00008a037777_P01, 3 exons and Zm00008a007621_P01, 4 exons) were observed to have distinctive gene structures. Although the dissimilation of gene structure of each member of SF1s was substantial, we found that the length of CDSs did not significantly change (Fig. 2). Thus, whether it influences the differentiation of their gene function needs to be further investigated. Further investigation on conserved motifs by using Multiple Em for Motif Elicitation (MEME) search tool demonstrated that most *SF1* genes (79 sequences) exhibited similar sequence signatures and the same order and all contained the 10 analysed motifs, except one sequence of *Micromonas pusilla* (50949) had a different position (Fig. 2, right panel). Although no obvious differences in identified conserved motifs were found among basal angiosperm, monocots and eudicots, sequences from the same species were found to have different motifs (Fig. 2). For example, Aqcoe5G406900.1.p and Aqcoe7G039300.1.p from the eudicot *Aquilegia coerulea* had 10 motifs and 9 motifs, respectively. The same situation was found in *D. carota*; DCAR_006843, DCAR_008506 and DCAR_004968 had 10 motifs, 9 motifs and 10 motifs, respectively. Intriguingly, the CDS length of DCAR_008506 was the longest. Notably, some sequences from algae and moss had fewer conserved motifs. For example, in bryophyta, the sequences of *Physcomitrella patens* (Pp3c7_10890V3.1.p and Pp3c11_24710V3.1.p), *Sphagnum fallax* (Sphfalx0015s0077.1.p and Sphfalx0010s0197.1.p) and *Marchantia polymorpha* (Mapoly0009s0189.1.p) had nine motifs. In algal plants, the sequences of 145,219 and 62,857 from Micromonas had only 7 motifs and 6 motifs, respectively. Moreover, although the sequences of *Volvox carteri* (Vocar.0007 s0345.1.p and Vocar.0008 s0294.1.p) and *Chlamydomonas reinhardtii* (Cre12.g553750.t1.1 and Cre09.g386731.t1.1) contained multiple exons, they had 9 motifs, indicating their sequence variation had little influence on function classes. Further correlation analysis of the SF1 exon regions were carried out to elucidate the gain/loss of introns. Correlations between transcripts of plant SF1s are shown in Fig. 3, providing additional information for phylogenetic analysis. For example, there is more similarity between PGSC0003DMT400081859 and Migut.D02531.2 because of multiple exact matches between the exons of the two transcripts.

### Analysis of protein domain and conserved motifs in peptides

**Fig. 4:**
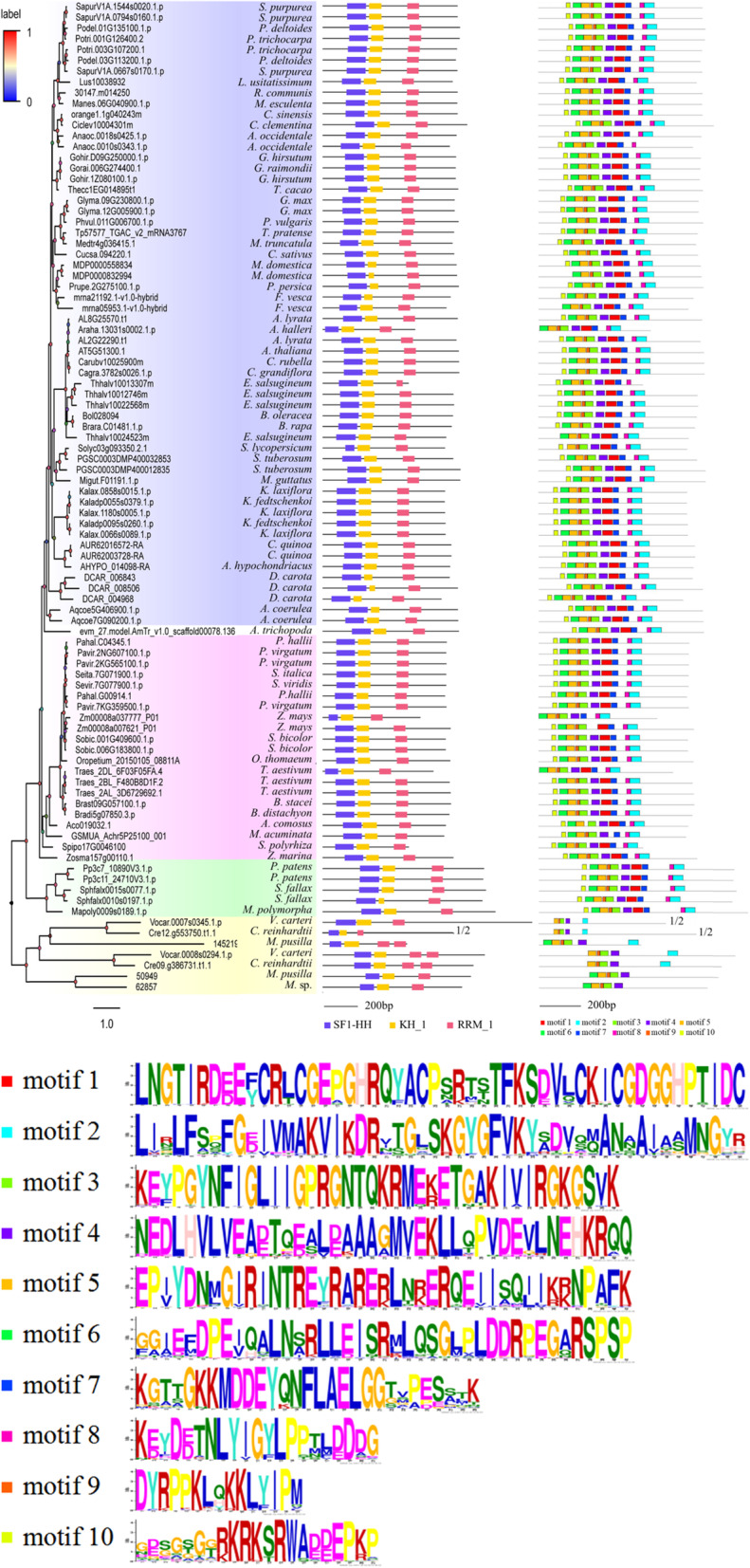
Comparisons of protein domains and conserved motif identification among plant *SF1* genes. Protein domain (middle panel) and identified protein conserved motifs (right panel) identified by MEME analysis are shown against the vertical phylogenetic tree (left panel). The conserved sequence of 10 identified motifs represented by different coloured boxes are listed below

The protein domains were analysed by using the above selected 92 peptide sequences from 59 plant species; the peptides’ annotations were splicing factor-related and conserved protein motifs were predicted according to the retrieved peptide sequences by MEME analysis (Fig. 4). Consequently, all SF1s were found having SF1_HH N-terminal domain on the N-terminal of the peptides followed by a KH domain and a C-terminal domain, namely, an RNA recognition motif (RRM) (Fig. 4, middle panel). Interestingly, in algae, 3 peptides from *M. pusilla* (145219), *V. carteri* (Vocar.0008 s0294.1.p) and *C. reinhardtii* (Cre09.g386731.t1.1) had two RRM domains. The amino acid lengths of SF1 proteins ranged from 499 aa to 1583 aa, and most of them possessed 700 to 800 amino acids (Table S3). Consistently, most of them are approximately 700 to 800 amino acids in length. Subcellular location prediction showed that the majority of SF1 proteins were had nuclear localization (86, 93.4%) (Table S3). Moreover, proteins of 30,147.m014250 (*Ricinus communis*) and Migut.F01191.1.p (*Mimulus guttatus*) were located in the vacuoles; proteins of Traes_2DL_6F03F05FA.4 (*T. aestivum*) and 145,219 (*M. pusilla*) were predicted to be cytoplasmic; proteins of GSMUA_Achr5P25100_001 (*Musa acuminata*) and Cre09.g386731.t1.1 (*C. reinhardtii*) were located in the chloroplast and endoplasmic reticulum, respectively.

MEME analysis for SF1 peptide sequences was used to predict a total of 10 conserved motifs, which are presented as coloured boxes and cover most of the protein (Fig. 4, right panel). Further analysis showed that 77 peptides had all 10 motifs, accounting for approximately 83.7% of all SF1 protein sequences analysed in the study. Interestingly, all sequences from moss have 10 conserved motifs in the analysis, suggesting the conservation of SF1 proteins in bryophyta. Furthermore, almost all eudicots had 10 conserved motifs—except *Anacardium occidentale* (Anaoc.0018 s0425.1.p) and *C. grandiflora* (Cagra.3782 s0026.1.p) which lacked motif 2 and *Malus domestica* (MDP0000558834), *Fragaria vesca* (mrna21192.1-v1.0-hybrid) and *Brassica rapa* (Brara.C01481.1.p) which lacked motif 10—while most monocots had eight conserved motifs. In contrast, algal plants only possess approximately half of the predicted 10 motifs due to their peptides with integrant protein domains, implying the least degree of conservation and divergence of plant SF1 proteins in algae. T motifs that all algae shared were motif 3, motif 4, motif 5 and motif 9.

### Analysis of promoter and tissue-specific expression of *SF1* genes

**Fig. 5:**
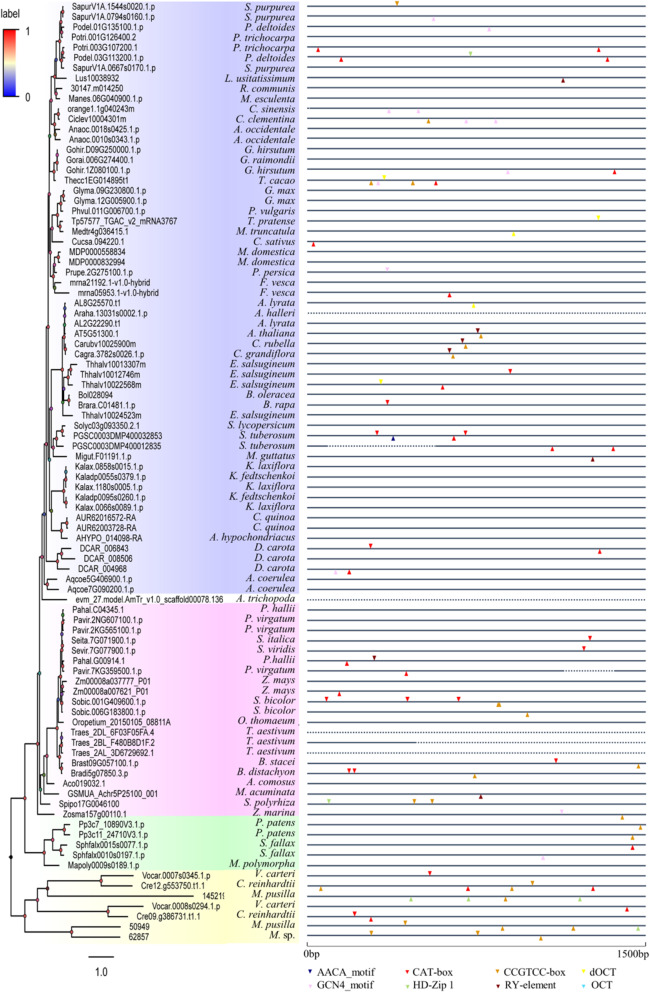
Analysis of motifs related to tissue specificity in the plant *SF1* promoter regions. Eight cis-acting motifs are represented in different color triangles. Positions of these identified motifs are labeelled along the 1.5 kb 5′-flanking regions of each *SF1* gene. The line solid and dotted represents regions with basic pairs and regions of no sequences or annexed base N respectively. Symbols on above the line represent the motifs at the plus strand, whereas symbols on below the line represent the motifs at the minus strand. Function of motifs: AACA-motif, involved in endosperm-specific negative expression; CAT-box, cis-acting regulatory element related to meristem expression; CCGTCC-box, cis-acting regulatory element related to meristem specific activation; dOCT, cis-acting regulatory element related to meristem specific activation; GCN4_motif, cis-regulatory element involved in endosperm expression; HD-Zip1, element involved in differentiation of the palisade mesophyll cells; RY-element, cis-acting regulatory element involved in seed-specific regulation. The black vertical lines represent break at that particular branch; OCT, cis-acting regulatory element related to meristem specific activation

**Fig. 6:**
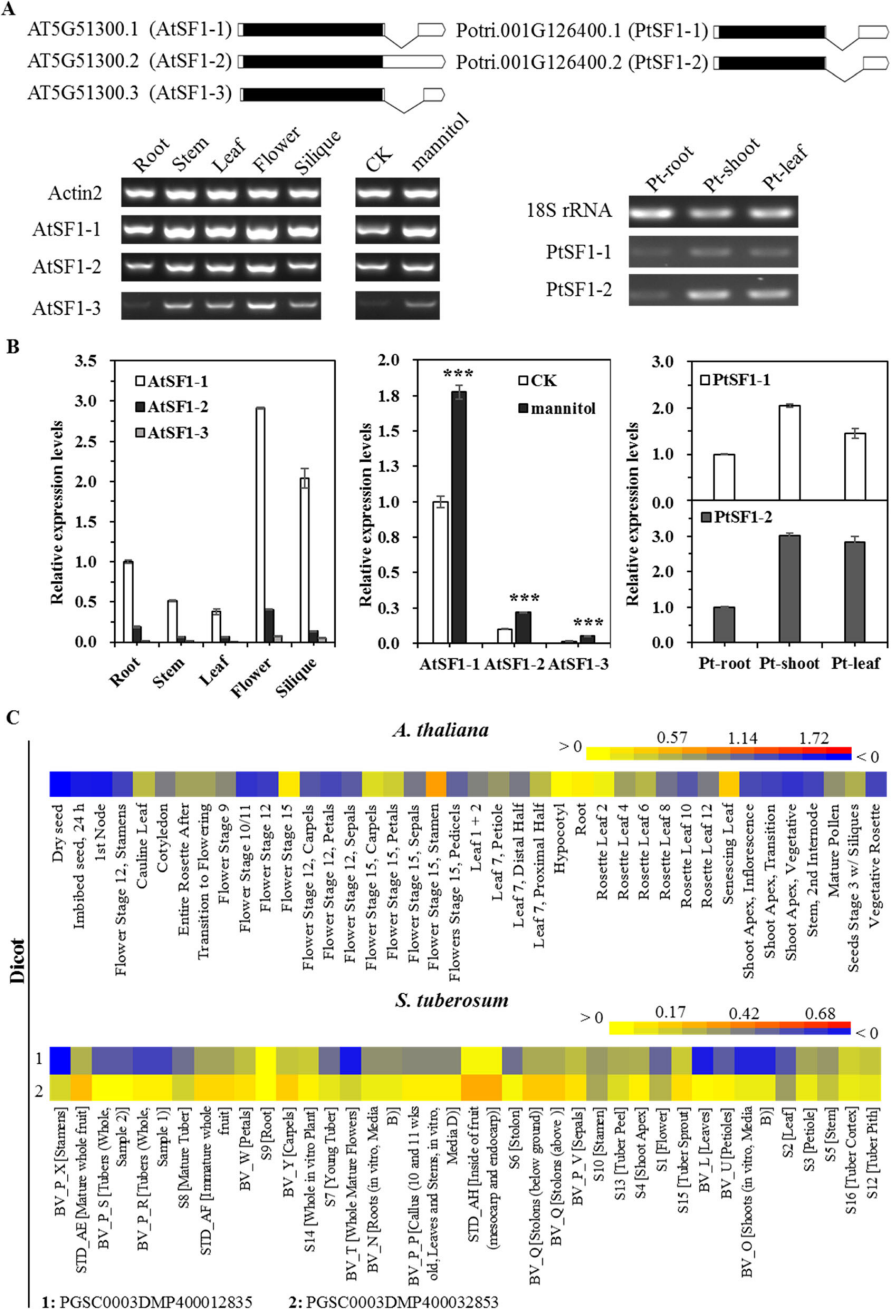
Expression patterns analysis of SF1 genes in several plants. **a** RT-PCR analysis of AtSF1 and PtSF1 associated with their isoforms expression in roots, shoots, leaves, and flowers. Gene models of each isoform (AtSF1 and PtSF1) are indicated (black, coding region; white, non-coding untranslated regions). RNA samples of mannitol treatment were prepared from 7-day-old seedlings at the exposure to 350 mM mannitol. The Arabidopsis actin2 and the poplar 18S rRNA gene were used as the internal expression control. **b** Real-time RT-PCR expression analysis of AtSF1 and PtSF1 associated with their isoforms. **c** Expression patterns of *Arabidopsis* and *Solanum tuberosum* (potato) *SF1* genes. Expression data were obtained from the plant eFP browser microarray datasets, transformed by Lg conversion and presented as a heatmap. Red colour represents high levels of transcript abundance, and blue represents low transcript abundance

To further analyse the regulation of plant *SF1* genes at the transcriptional level, the 1.5-kb upstream sequences of plant *SF1* genes were obtained from the Phytozome database, then the *cis*-elements of each promoter were identified by using the PlantCARE program (Table S4) [37]. Consequently, a total of 108 motifs were predicted. Generally, eight *cis*-elements related to tissue-specific expression among them were selected (Fig. 5 and Table S4), including HD-Zip1 for differentiation of the palisade mesophyll cells, the RY-element which regulates seed-specific expression, the AACA_motif and GCN4_motif involved in endosperm expression, and the CAT-box, CCGTCC-box, dOCT, and OCT for meristem expression. Further analysis showed that there were only 50 promoters of *SF1* genes which had tissue-specific regulatory *cis*-elements. Particularly, the CAT-box and CCGTCC-box turned up at the highest frequency and greatest abundance in the promoters of *SF1* genes. Both of them regulate meristem-specific expression and play key roles during development and growth of plants. Consistently, purple false brome (*Brachypodium distachyon*) of monocots not only had a CAT-box and CCGTCC-box, but was also highly expressed in young leaves, internode, adventitious roots and roots (Fig. 5 and Figure S2). However, no motifs were found to link the high expression of two *SF1*s of *Glycine max* (soybean) in SAM and root-tip (Figure S1). Additionally, the AACA_motif was only detected in *Solanum tuberosum* (PGSC0003DMP400032853) of potato, suggesting its specific role in regulating endosperm-specific negative expression. Further, HD-Zip 1 was present in Podel.03G113200.1.p of *Populus deltoides* (eastern cottonwood) and Spipo17G0046100 of *Spirodela polyrhiza* (greater duckweed). The RY-element was detected in the promoter of the dicot model plant *Arabidopsis*, and low expression was also reported in dry seed in *Arabidopsis* (Fig. 6), suggesting that the RY-element is involved in seed-specific negative expression of *Arabidopsis.* Moreover, expression levels in the same tissue type showed significant differences during different growth stages; for example, the expression level in stamen of flower stage 15 of *Arabidopsis* was obviously higher than that of the other flower development stages. However, the expression levels of different growth stages of *Solanum lycopersicum* were not only similar but lower, and no motifs were found in the promoter in tomato (Figs. 5 and S1). Furthermore, different expression patterns were detected in several *SF1* genes with multiple copies (Figs. 6, S1 and S6). For instance, similar tissue expression profiles were detected in two *SF1* homologues from the dicot *Populus trichocarpa* (Potri.001G126400.1 and Potri.003G107200.1) and the monocot *Zea mays* (Zm00008a007621_P01 and Zm00008a037777_P01) (Figure S1 and S5). In contrast, two *SF1* genes of *S. tuberosum* showed differential expression patterns, similar to in *G. max* (Figs. 6 and S1).

### Analysis of promoter and internal and external hormones expression of *SF1* genes

**Fig. 7:**
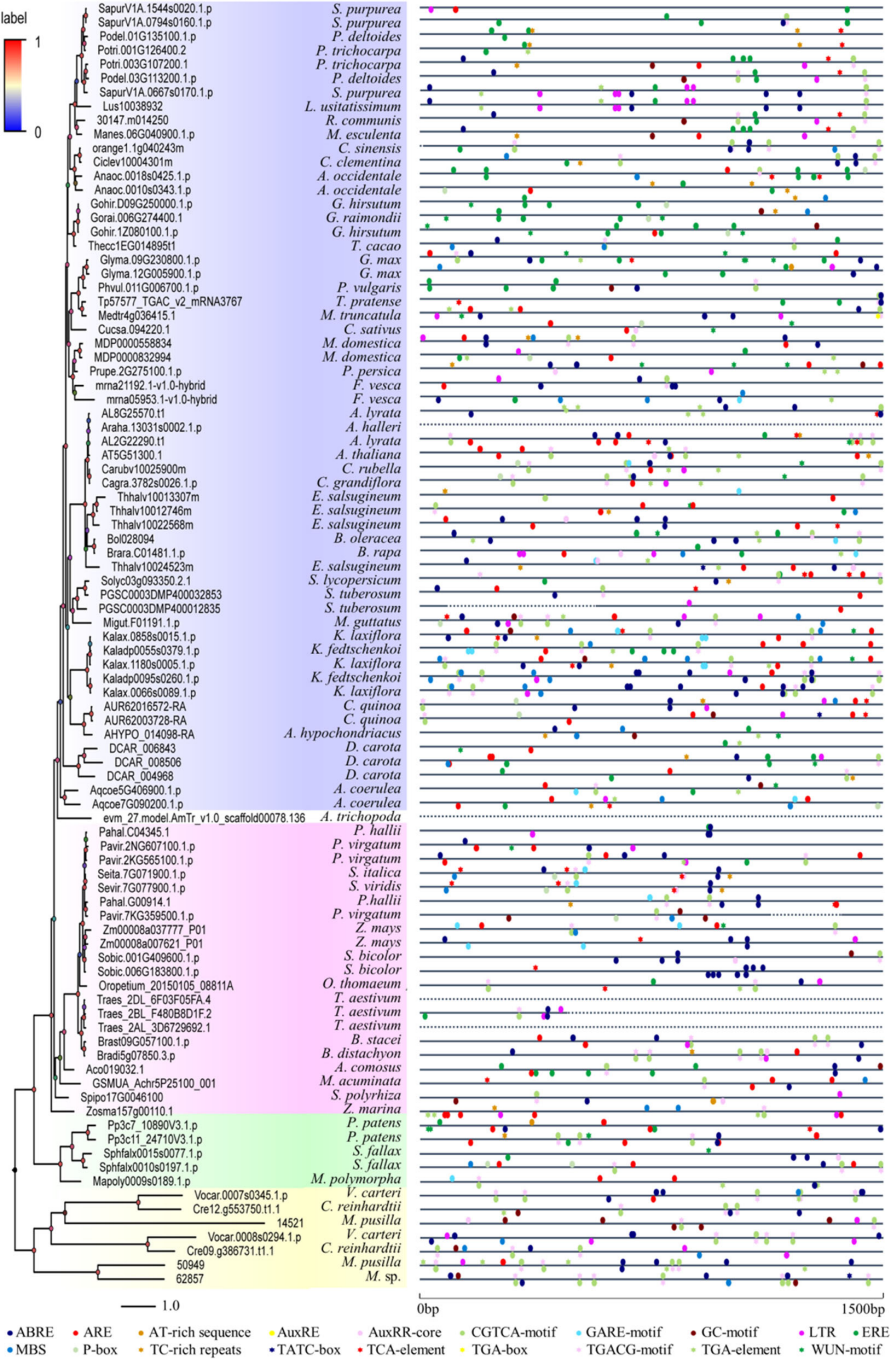
Analysis of motif-related hormone and stresses in the plant *SF1* promoter regions. Nineteen cis-acting elements are represented in different color symbols. Positions of these identified motifs are labeelled along the 1.5 kb 5′-flanking regions of each *SF1* gene. The line solid and dotted represents regions with basic pairs and regions of no sequences or annexed base N respectively. Symbols on above the line represent the motifs at the plus strand, whereas symbols on below the line represent the motifs at the minus strand. Function of motifs: ABRE, cis-acting element involved in the abscisic acid responsiveness; ARE, cis-acting regulatory element essential for the anaerobic induction; AT-rich sequence, element for maximal elicitor-mediated activation (2copies); AuxRE, part of an auxin-responsive element; AuxRR-core, cis-acting regulatory element involved in auxin responsiveness; CGTCA-motif, cis-acting regulatory element involved in the MeJA-responsiveness; ERE, ethylene-responsive element; GARE-motif, gibberellin-responsive element; GC-motif, enhancer-like element involved in anoxic specific inducibility; LTR, cis-acting element involved in low-temperature responsiveness; TATC-box, cis-acting element involved in gibberellin-responsiveness; TCA-element, cis-acting element involved in salicylic acid responsiveness; MBS, MYB binding site involved in drought-inducibility; P-box, gibberellin-responsive element; TC-rich repeats, cis-acting element involved in defence and stress responsiveness; TGA-box, part of an auxin-responsive element; TGACG-motif, cis-acting regulatory element involved in the MeJA-responsiveness; TGA-element, auxin-responsive element; WUN-motif, wound-responsive element

In long-term evolution and development, plants have gradually formed mechanisms of adaptation and resistance to adversity to maintain their life and sustain growth. To understand the regulatory mechanisms of internal and external stimuli on plant *SF1s*, cis-acting elements involved in hormone and stress were studied with the PlantCARE database (Fig. 7, Table S4). Finally, 19 hormone- and stress-related motifs were selected from 92 promoter sequences of plant *SF1s*. There are 12 hormone-related motifs including abscisic acid (ABRE), auxin (AuxRE, AuxRE-core, TGA-box, TGA-element), ethylene (ERE), gibberellin (GARE-motif, P-box, TATC-box), MeJA (CGTCA-motif, TGACG-motif), and salicylic acid (TCA-element) and five stress-related motifs including low-temperature (LTR), drought (MBS), wound (WUN-motif) and anoxic (ARE, GC-motif) motifs. Almost each *SF1* sequence had a great diversity of *cis*-elements in its promoter regions except some sequences such as Araha.13031 s0002.1 and Traes_2AL_3D6729692.1 which did not contain a single motif due to the sequences contain ‘N’ or no promoter, suggesting that multiple hormones-mediated signalling pathways are closely related to *SF1* plants resistance. Analysis showed that more than half of *SF1* promoters contained ABRE, CGTCA-motif, TGACG-motif and ARE, respectively. Moreover, external hormone signals also affect the abundance of *SF1* transcripts (Figure S3). For example, in *Arabidopsis* (AT5G51300.1), MJ (methyl jasmonate) inhibited its expression (Fig. 7), and treatment with other hormones like ACC (a precursor of ethylene), IAA (auxin), ABA and GA (gibberellin) regulates the expression of AT5G51300.1.

### Analysis of protein-protein interaction network and structural conservation

**Fig. 8:**
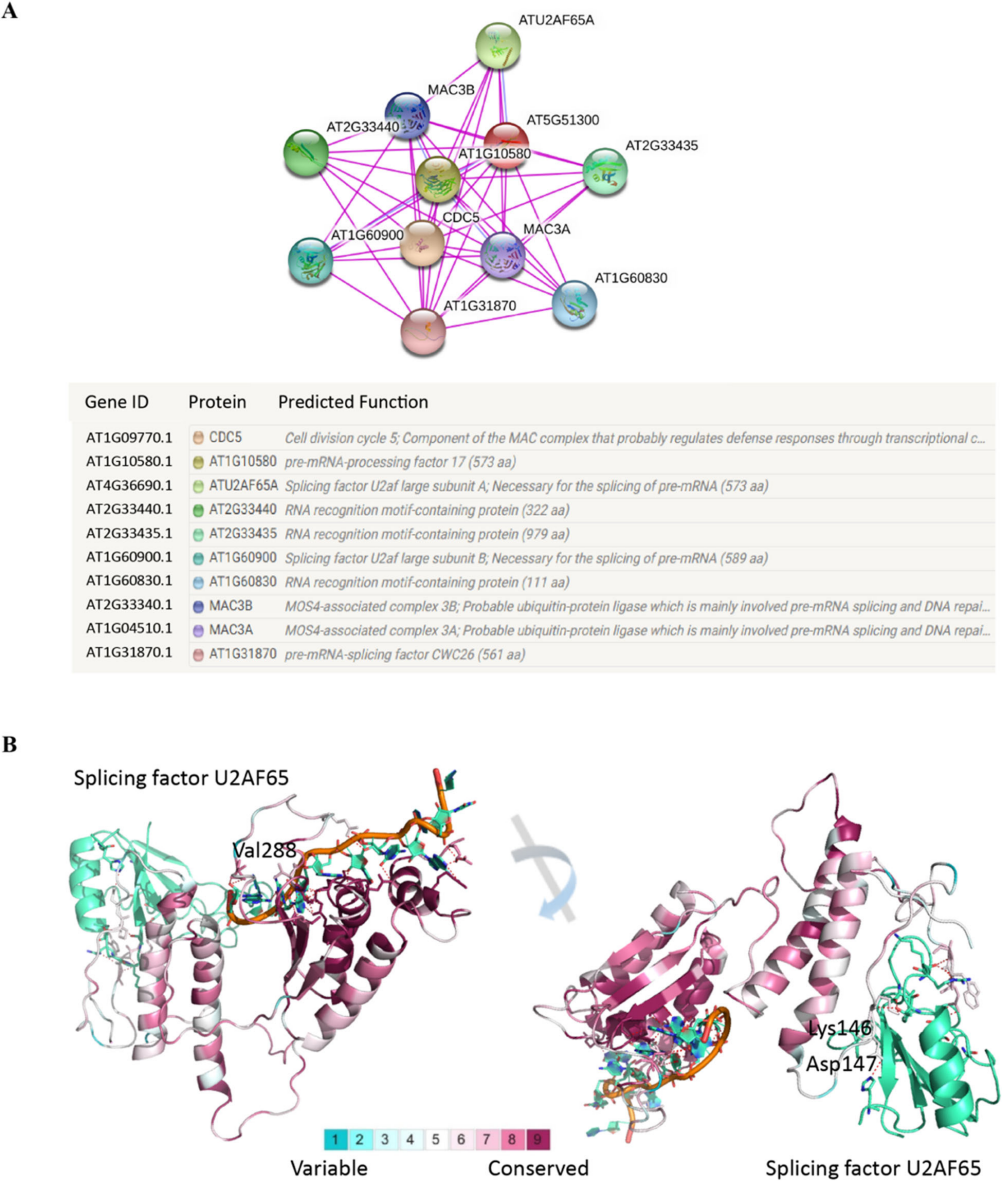
Representative interaction network and conserved amino acid sequence analysis of plant SF1s. **a** Interaction network of *Arabidopsis* (AT5G51300) based on experimental data. Each network node represents all proteins produced by a single, protein-coding gene locus. Different coloured nodes represent query proteins and the first shell of interactors. Filled nodes represent that some 3D structure is known or predicted, while empty nodes represent proteins of unknown 3D structure. Edges represent protein-protein associations in which proteins jointly contribute to a shared function. **b** Conserved domains of plant SF1s. The 3D structure of plant SF1 were generated according to the *Arabidopsis* sequence (AT5G51300) and represented with their target RNA. The ribbon colored by the ConSur Grade (1-blue to 9-purple) represent the conservation grades of the identified peptides of SF1s

Protein-protein interaction (PPI) network analysis can systematically reveal the working principle of proteins in biological systems, the molecular mechanisms of biological signals and energy metabolism, and the functional relationships between proteins. In this study, we generated protein-protein interaction networks of the SF1 protein according to the representative protein sequence of *Arabidopsis* (AT5G51300) using the STRING database based on experiments (Fig. 8a). Finally, 10 predicted functional partners of the SF1 protein were obtained, including CDC5 (AT1G09770.1), AT1G10580 (AT1G10580.1), ATU2AF65A (AT4G36690.1), AT2G33440 (AT2G33440.1), AT2G33435 (AT2G33435.1), AT1G60900 (AT1G60900.1), AT1G60830 (AT1G60830.1), MAC3B (AT2G33340.1), MAC3A (AT1G04510.1), and AT1G31870 (AT1G31870.1) (Fig. 8a). CDC5, MAC3A and MAC3B are components of the MAC complex that probably regulate defence responses through transcriptional control and thereby are essential for plant innate immunity. All of them may be involved in pre-mRNA splicing and DNA repair. AT1G10580 is pre-mRNA-processing factor 17, and AT1G31870 is splicing factor CWC26. Both proteins participate in RNA splicing and pre-mRNA processing. AT2G33440, AT2G33435 and AT1G60830 are RNA recognition motif-containing proteins whose main molecular functions are involved in pre-mRNA splice site binding. ATU2AF65A and AT1G60900 are splicing factor U2af large subunit A and B, respectively, and they are necessary for the splicing of pre-mRNA. AT5G51300 (splicing factor-like protein 1) has already been demonstrated to be necessary for the splicing of pre-mRNA, development, and abscisic acid (ABA) responses. In general, SF1 protein and its functional partners are generally involved in RNA splicing and pre-mRNA processing, and some of them also possess functions in defence response to bacteria (Fig. 8a).

The *A. thaliana* SF1 protein includes three domains: splicing factor 1 helix-hairpin domain (residue: 126–237), KH domain (residue: 244–330) and RNA recognition motif (residue: 482–552). Multiple-sequence alignment revealed that the conservations of these domains are relatively high (Figure S4), suggesting similar functions of these genes. Furthermore, a 3D model of the splicing factor 1 helix-hairpin domain and KH domain were reconstructed according to two crystal structures by using a homology modelling approach (Fig. 8b). The first domain (helix-hairpin domain) forms a secondary, hydrophobic interface with U2AF65 (UHM) [80]. The second one (KH domain) is present in a wide variety of nucleic acid-binding proteins [15]. Therefore, we superimposed the crystal structure of U2AF65 (2M0G) and RNA (1K1G) on the structure from homology modelling to observe the interaction. The residues with higher ConSurf Grade are more conserved. The ConSurf Grade of 198 (74.4%) residues was over 7, and the ConSurf Grade of 111 (41.7%) residues was over 9. More importantly, the binding domain of RNA was highly conserved (Fig. 8b). All of the import residues had a ConSurf Grade higher than 7, except for Val288. The residues at position 288 have similar physiochemical properties, such as Val and Ile. Another domain was not as preserved as splicing factor 1 helix-hairpin domain with a loop interacting with U2AF65. However, the important residues have relatively high ConSurf Grade, and only two residues (Lys146 and Asp147) have ConSurf Grades less than 7. In the lower plants, these two residues are replaced by Ile, Gly, Tyr, Thr, Ala and Gly, Ser, or His. At the same time, they are lost in many species. Therefore, the functions of these domains are conserved. The RNA binding domain is much more conserved than the U2AF65 binding domain, especially in lower plants.

## Discussion

It is well known that mature mRNA is formed by sequentially ligating exons to maintain a particular reading frame for protein translation [60]. In human, nearly all annotated protein-coding genes undergo alternative splicing [56, 75]. In plants, over 80% of intron-containing genes exhibit splicing isoforms [11, 82]. Furthermore, the process of splicing is tightly regulated by initial recognition of the splice site during early spliceosome assembly. Therefore, proteins which are responsible for this recognition are important to study and provide valuable targets for genetic control of splicing in eukaryotes [35, 71]. To this end, the branch point binding protein SF1, which connects both 5′ and 3′ splice site determination complexes, emerges as crucial component for splice site choice.

### Comparison of structural and functional conservation among plant *SF1* genes

In this study, we systematically characterized 92 plant *SF1* genes from 59 different species. Although over 50% (34/59) of these species maintained one copy of *SF1* gene, 26 plant species contained multiple *SF1* members (Table S1), suggesting their functional redundancy. Intriguingly, most of the *SF1* genes had one single exon encoding the target protein product except for several algal sequences (Fig. 2), indicating that an ancient gene transposition duplication event may have influenced the evolution of this gene family across the plant lineage [24]. However, further evidence is needed to confirm this hypothesis. At the molecular level, SF1 is an important component to mediate early spliceosome assembly and splice site recognition. Therefore, substantial investigations have been carried out to elucidate its molecular function in both animals and plants. For example, the primary amino acid sequence and domain architecture of SF1 proteins have been reported to be conserved among eukaryotic organisms such as yeast, human, metazoans and plants [2, 6, 30, 47]. SF1 proteins are normally characterized by three domains: KH/QUA2, zinc finger and RRM [36]. However, plant SF1 proteins have been documented to contain an additional RRM domain while lacking UHM-specific features [36]. A previous study demonstrated that a truncated plant SF1 protein without an RRM domain still has sufficient activity for pre-mRNA splicing in response to ABA treatment [36]. Thus, the potential function of this additional domain in planta needs to be further investigated. Furthermore, post-translational modification such as serine phosphorylation by KIS kinase has been reported to enhance the assembly of the SF1–U2AF65–RNA tri-complex [45, 80] or to recruit other splicing factors during splice site recognition [2, 28].

### Functional diversification of plant *SF1* genes revealed by their expression patterns

SF1 is considered a pivotal component connecting the 5′ and 3′ splice site definition complexes. Furthermore, substantial evidence has demonstrated that SF1 plays crucial roles during splice site recognition among a variety of eukaryotic organisms [46, 52, 68]. However, its role in cell viability remains disputed. Accumulating evidence suggests that SF1 may not be essential for viability and may only control subsets of genes in plants and animals [22, 83], indicating that an alternative mechanism may exist in addition to SF1-mediated splice site recognition [23, 46, 72]. Furthermore, the function of SF1 can be further affected by cell, tissue, or organ specificity. For example, mouse *SF1* transcripts have been detected in the brain and heart, implying their tissue-specific regulation at the transcriptional level [83]. Additionally, SF1 is highly expressed in differentiated villous cells, but it is not observed in adenoma or undifferentiated intestinal crypt cells of the intestinal epithelium [49]. In plants, interestingly, SF1 has been found to be involved in a number of plant developmental processes and stress responses [30, 36]. In particular, SF1 has been observed to influence flowering time and leaf size in *Arabidopsis* and *Populus*, coincident with its relative high expression in flower parts and leaves (Fig. 6a). Importantly, the SF1 splicing isoforms also exhibit similar expression pattern as SF1 by our qRT-PCR and RT-PCR expression analysis, implicating a reciprocal regulation between SF1 expression and splicing differences during flower and leaf development (Fig. 6a and b). Meanwhile, the expression of SF1 associated with their isoforms were strongly induced by mannitol treatment, indicating a potential function involving the drought stress. Furthermore, transcripts of SF1 are unevenly distributed in several monocots and eudicots (Figs. 6c, S1 and S2), suggesting their potential role during plant development in these species.

In comparison to tissue specificity, more *cis*-elements involved in hormone and stress responses were observed within promoter regions of plant *SF1* genes (Fig. 7 and Table S5), indicating their putative role in response to internal and external stimuli. The *Arabidopsis* SF1 has been demonstrated to participate in ABA signalling [30, 36], coinciding with the presence of an ABRE motif at its own 5′-flanking region (Fig. 7). Furthermore, *Arabidopsis SF1* is induced by IAA at 1 h after treatment and repressed by MeJA (MJ). The AuxRR-core and CGTCA-motifs observed in its promoter region may be responsible for this regulation (Fig. 7). However, further intergrated investigation by using both bioinoformatic and experimental data is required to further strengthen this hypothesis in future functional investigations [9, 10].

### Composition of splice site determination complex reveals diverged mechanism to define exon-intron boundary among eukaryotes

In general, eukaryotic SF1s have similar molecular functions to mediate early splice site recognition. Specifically, *Arabidopsis* SF1 has been proposed to have similar function to its yeast or metazoan counterparts [30, 36]. However, different eukaryotic organisms may evolve their own recognition mechanism during early spliceosome assembly through SF1. First, the target BPS of SF1 is distinct in yeast compared to the sequences in animals and plants. In particular, yeast intronic BPS is a conserved seven-nucleotide sequence (UACUAAC), whereas mammalian SF1 has been reported to bind more degenerate sequences (YNCURAY; N, any nucleotide; R, A or G; Y, C or U) [32]. No conserved BPS has been observed in nematodes and plants at this stage [40, 42]. This poses the question of how SF1 recognizes the BPS in these organisms and whether the additional RRM in plants contributes to this recognition [30]. Second, different coordinative mechanisms are present in a variety of organisms. For example, as the interaction partner of SF1 to coordinate 3′ splice site recognition, mammalian U2AF65 interacts with U2AF small subunit (U2AF35). A similar interaction complex has been found in fission yeast, *S. pombe*, except the small U2AF subunit is named U2AF23 [69]. In contrast, budding yeast lacks a U2AF35-like small U2AF factor, and the other two proteins (BBP/SF1 and Mud2p/U2AF65) are proposed to form a stable complex during splicing [55]. Furthermore, splicing reactions in animals requires the binding of U2AF65 to Py sequences downstream of BPS, while neither of these two components are necessary for yeast splicing [1, 59]. Intriguingly, plants show a distinct splicing pattern in comparison to animals. For example, a high proportion of intron-retention events has been observed in plants, whereas exon skipping is the dominant AS type in animals [55]. SF1 has been proposed to enhance splicing efficiency of introns containing weakly conserved 3′ splice sites in *C. elegans* [41]. Therefore, it is tempting to speculate that this difference may result from different SF1-centred splice site recognition between animals and plants.

## Conclusion

In this work, we comprehensively identified 92 *SF1* sequences from 59 plant species, ranging from algae to eudicots. Subsequent phylogenetic and expression analyses have been carried out to elucidate the conservation and functional regulation of this gene family. By considering the connecting role of SF1 during splice site recognition, we hypothesize that plant SF1s may overlap with but also have distinct function from their animal counterparts. Understanding the molecular mechanism of this protein family in plants provides intriguing possibility to manipulate crop traits through genetic control of plant splicing.

## Supplementary information

**Additional file 1:**: **Figure S1.** Expression patterns of *Glycine max* (soybean), *Solanum lycopersicum* (Tomato) and *Populus trichocarpa* (Poplar) *SF1*s. **Figure S2.** Expression pattern of *Brachypodium distachyon* (Purple false brome) *SF1*. **Figure S3.** Expression of *Arabidopsis SF1* gene is affected by multiple phytohormone treatments.

**Additional file 2:** **Figure S4.** Multiple alignment of plant SF1 protein sequences.

**Additional file 3:** **Figure S5.** Expression patterns of *Zea mays* (maize) and *Kalanchoe fedtschenkoi* (diploid Kalanchoe) *SF1*s.

**Additional file 4:** **Figure S6.** The full uncropped gel photos of RT-PCR.

**Additional file 5:** **Table S1.** *SF1* genes identified from 59 plant species. **Table S2.** Characteristics of plant *SF1* gene structures. **Table S3.** Predicted subcellular localization of plant SF1 proteins. **Table S4.** Information of *cis*-elements identified among plant *SF1s*.

**Additional file 6:** **Table S5.** List of motifs identified in the 5′-flanking regions of plant *SF1s*.

**Additional file 7:** **Table S6.** Primers used for RT-PCR and qPCR analysis.

## Data Availability

The data are included within the article and its supporting files.

## Abbreviations

SF1/BBP: Splicing factor 1/branchpoint binding protein
BPS: Branchpoint sequence
RRM: RNA binding motif
AS: Alternative splicing
snRNPs: Small nuclear ribonucleoproteins
U2AF: U2 snRNP auxilliary factor
KH/QUA2: K homology/Quaking 2
hnRNP: Heterogeneous ribonucleoprotein
UHM: U2AF homology motif
ABA: Abscisic acid
SF1-HH: Splicing factor 1 helix-hairpin domain
KH_1: Homology domain
RRM_1: RNA recognition motif
GA: Gibberellin
IAA: Auxin
MJ: Methyl jasmonate
PPI: Protein-protein interaction
ML: Maximum likelihood
MEME: Multiple Em for Motif Elicitation
