# Supplementary material for: Systematic characterization of the branch point binding protein, splicing factor 1, gene family in plant development and stress responses
Source: BMC Plant Biol. 2020 Aug 18;20:379. doi: 10.1186/s12870-020-02570-6 (PMC7433366; doi:10.1186/s12870-020-02570-6)
Supplement: Supplementary file 1 — Additional file 1:: Figure S1. Expression patterns of Glycine max (soybean), Solanum lycopersicum (Tomato) and Populus trichocarpa (Poplar) SF1s. Figure S2. Expression pattern of Brachypodium distachyon (Purple false brome) SF1. Figure S3. Expression of Arabidopsis SF1 gene is affected by multiple phytohormone treatments. [file 12870_2020_2570_MOESM1_ESM.docx]

**
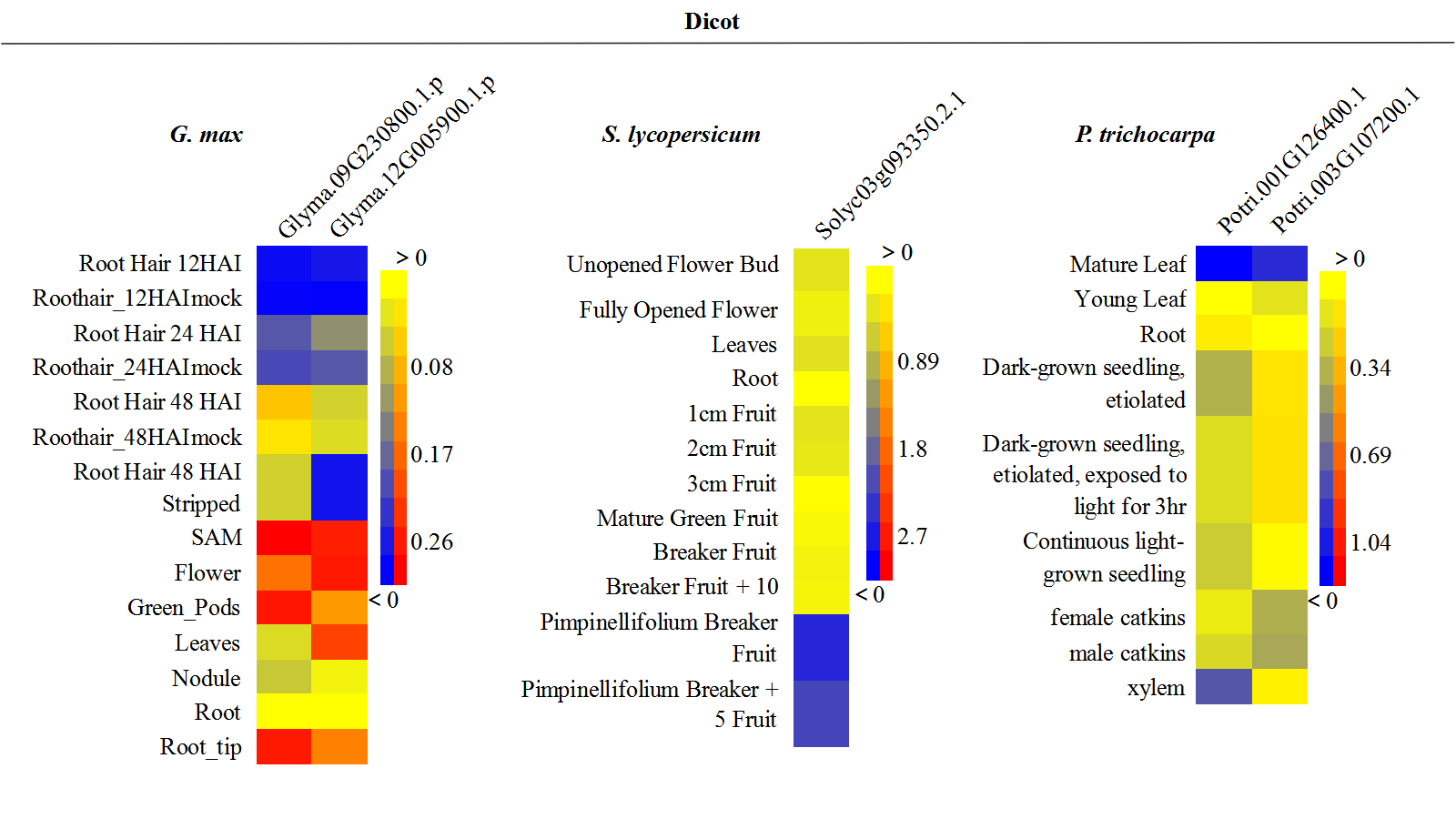
**

**Figure S1 Expressions patterns of *Glycine max* (soybean), *Solanum lycopersicum* (tomato) and *Populus trichocarpa* (poplar) *SF1*s.** Expression data were obtained from plant eFP browser microarray datasets and transformed by lg conversion and presented as heatmap. Red colour indicates high levels of transcript abundance and blue indicates low transcript abundance

**
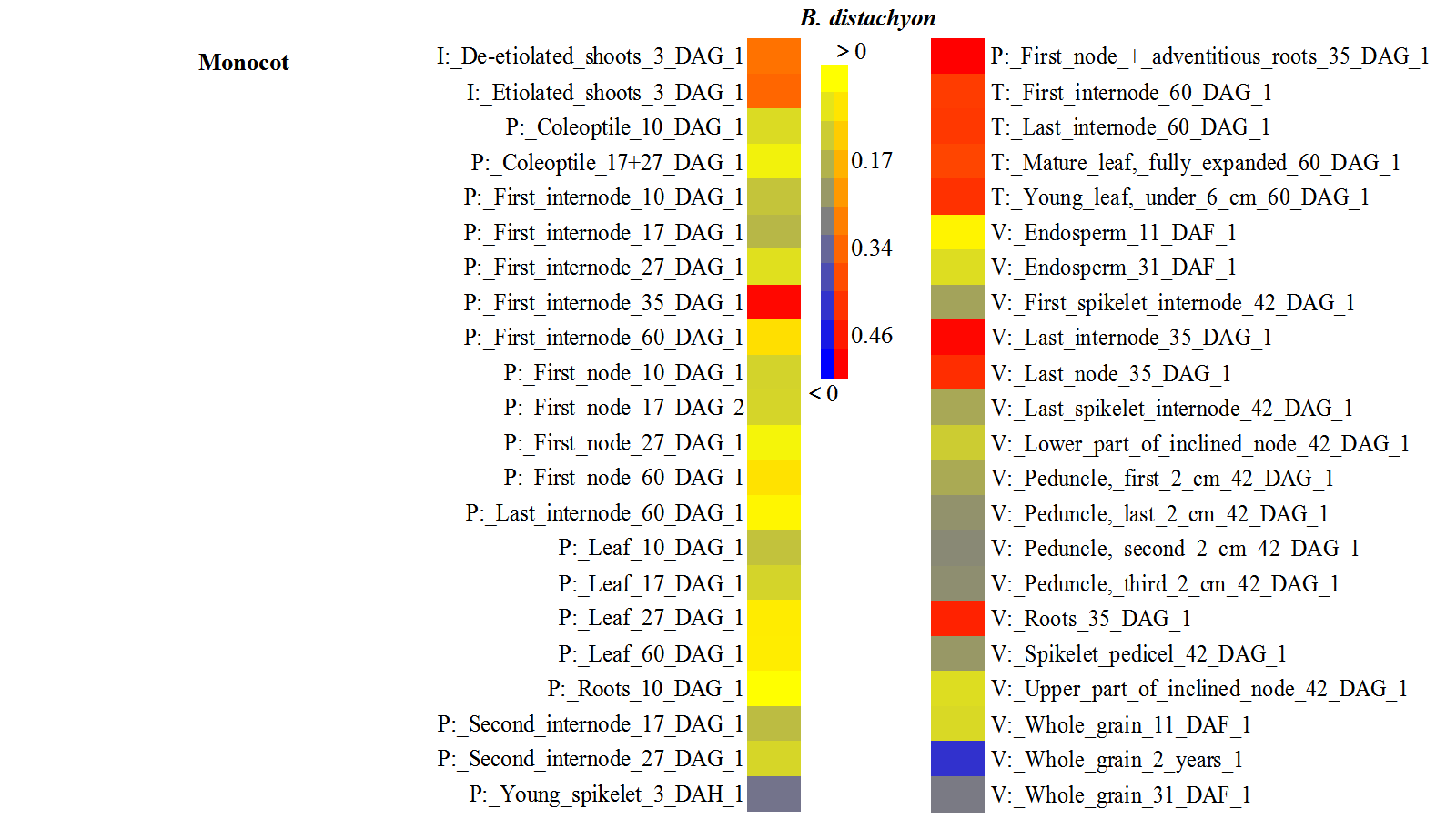
**

**Figure S2 Expressions patterns of *Brachypodium distachyon* (purple false brome) *SF1*.** Expression data were obtained from plant eFP browser microarray datasets and transformed by lg conversion and presented as heatmap. Red colour indicates high levels of transcript abundance and blue indicates low transcript abundance.


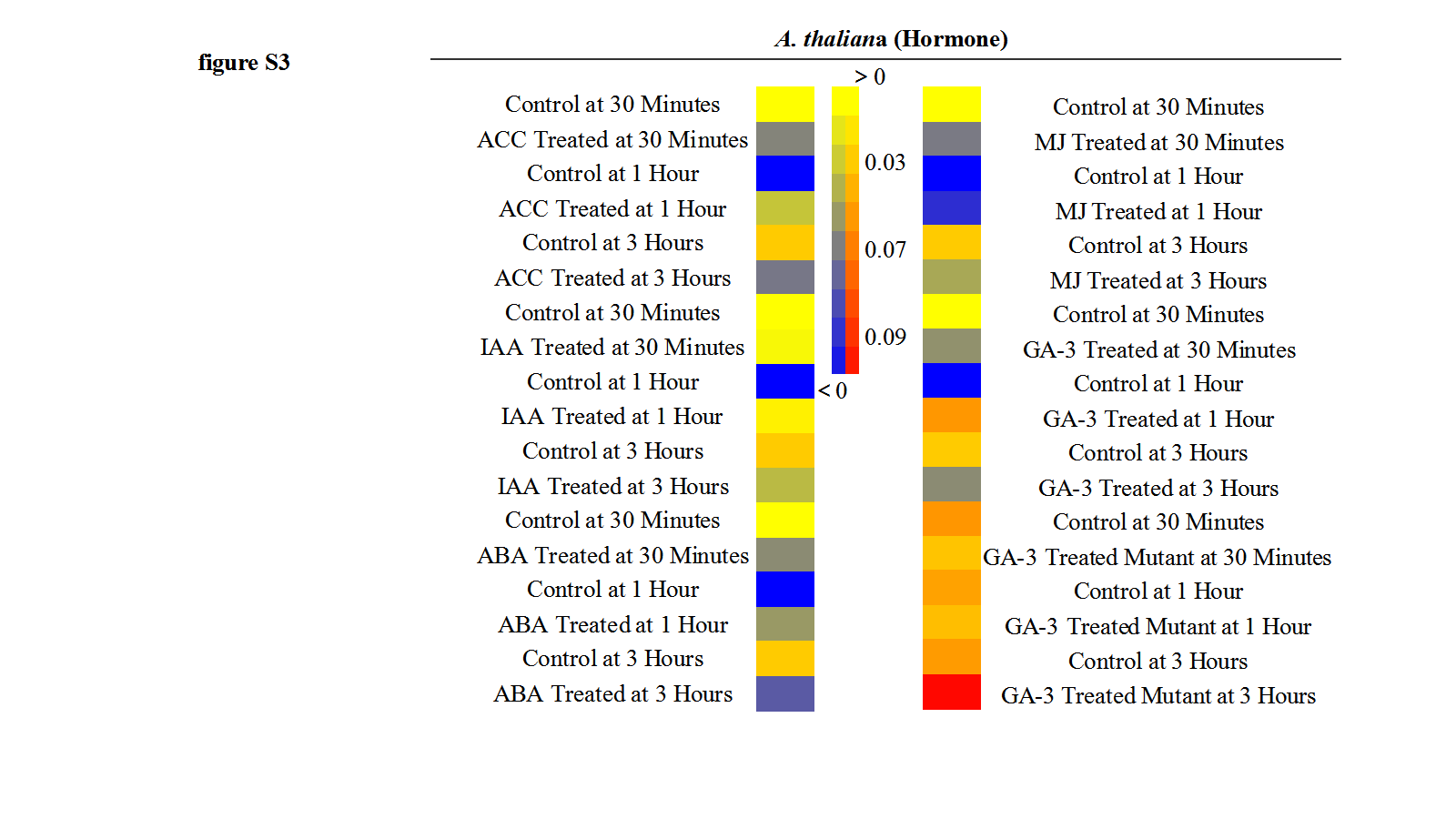


**Figure S3 The expression of *Arabidopsis SF1* gene is affected by multiple phytohormone treatments.** Expression data of Arabidopsis were downloaded from plant eFP browser microarray datasets and transformed by lg conversion and presented as heatmap. Red colour indicates high levels of transcript abundance and blue indicates low transcript abundance.
