# Supplementary figures and images for: Systematic characterization of the branch point binding protein, splicing factor 1, gene family in plant development and stress responses
Source: BMC Plant Biol. 2020 Aug 18;20:379. doi: 10.1186/s12870-020-02570-6 (PMC7433366; doi:10.1186/s12870-020-02570-6)

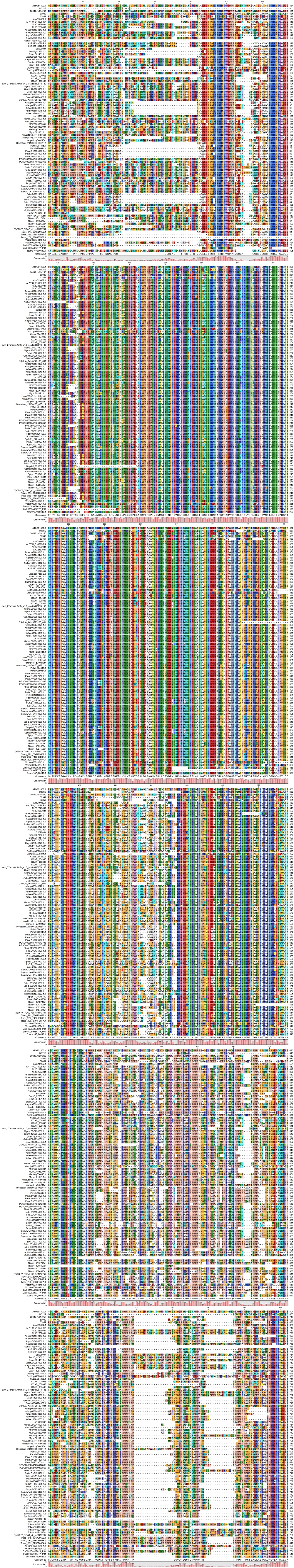

Supplement: Supplementary file 2 — Additional file 2: Figure S4. Multiple alignment of plant SF1 protein sequences. [file 12870_2020_2570_MOESM2_ESM.jpg]

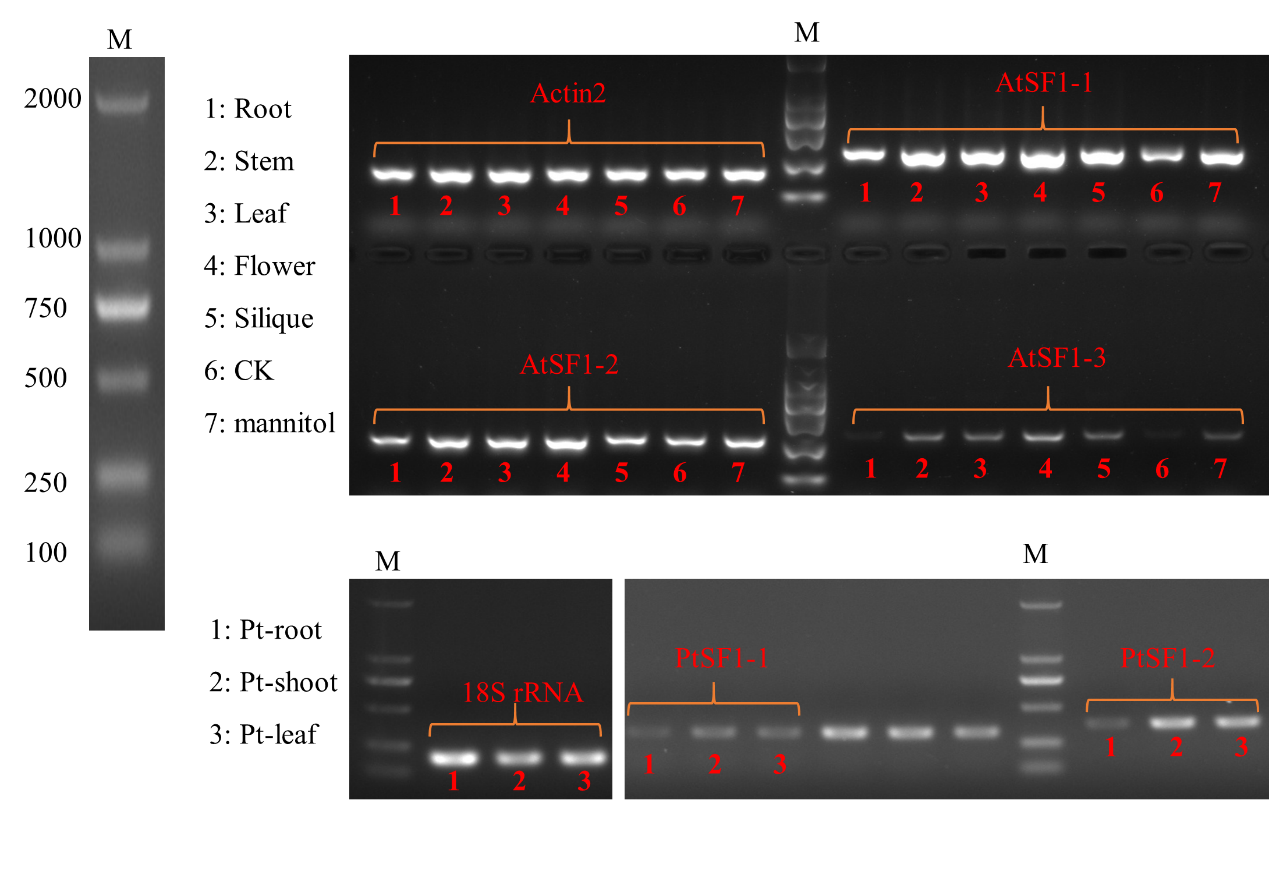
**Figure S6** The full uncropped gel photos of RT-PCR.

Supplement: Supplementary file 4 — Additional file 4: Figure S6. The full uncropped gel photos of RT-PCR. [file 12870_2020_2570_MOESM4_ESM.docx]
