# Supplementary material for: Systematic characterization of the branch point binding protein, splicing factor 1, gene family in plant development and stress responses
Source: BMC Plant Biol. 2020 Aug 18;20:379. doi: 10.1186/s12870-020-02570-6 (PMC7433366; doi:10.1186/s12870-020-02570-6)
Supplement: Supplementary file 3 — Additional file 3: Figure S5. Expression patterns of Zea mays (maize) and Kalanchoe fedtschenkoi (diploid Kalanchoe) SF1s. [file 12870_2020_2570_MOESM3_ESM.docx]

**
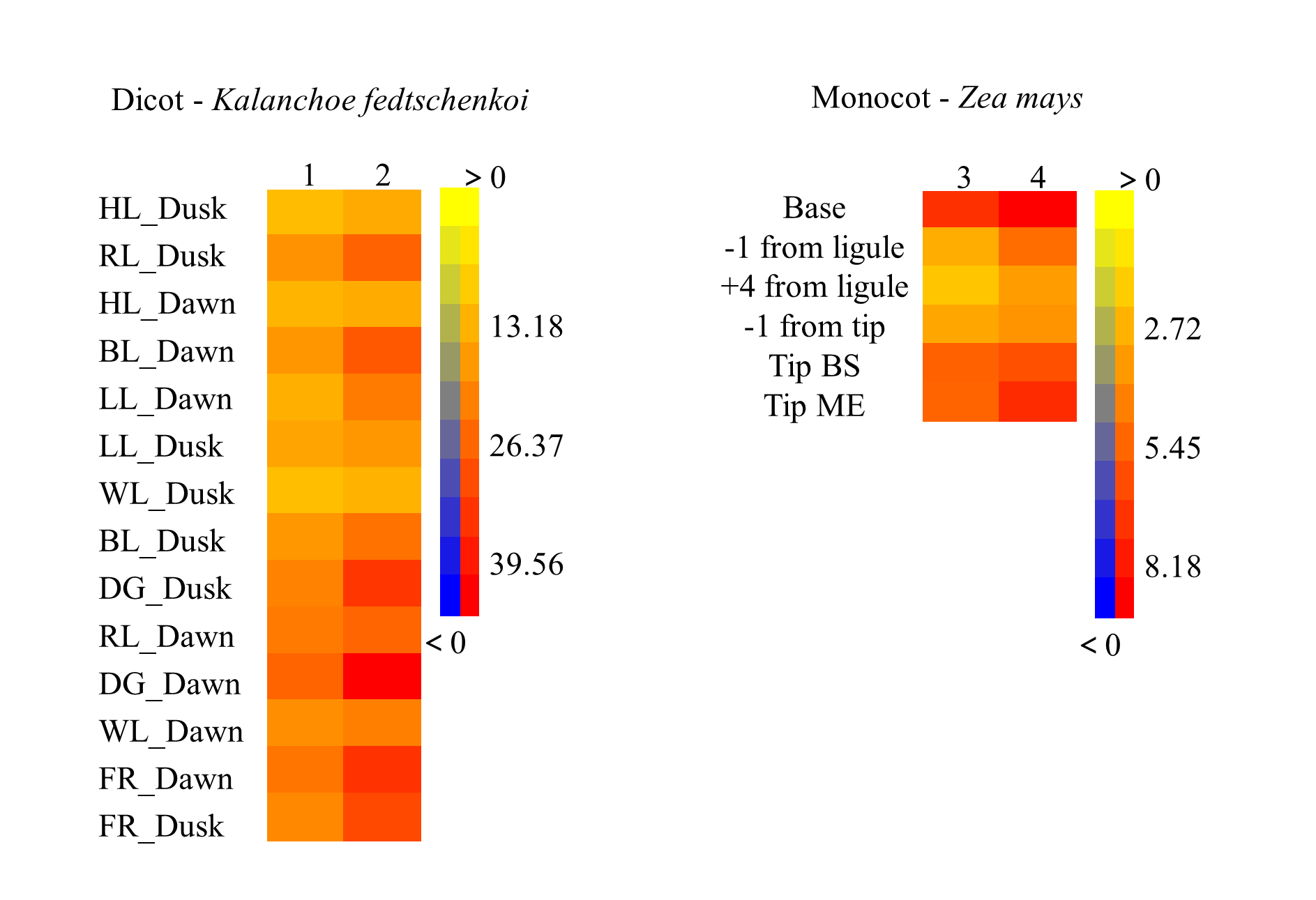
**

**Figure S5** **Expression patterns of *Zea mays* (maize) and *Kalanchoe fedtschenkoi* (diploid Kalanchoe) *SF1*s.** Expression data were obtained from plant eFP browser microarray datasets and presented as heatmap. Red colour indicates high levels of transcript abundance and blue indicates low transcript abundance. No.1-4 represents Kaladp0095s0260.1.p, Kaladp0055s0379.1.p, Zm00008a007621_P01, Zm00008a037777_P01, respectively. No.3-4 are known from previous assembly version as GRMZM2G000980 and GRMZM2G034313 respectively.
