# Supplementary material for: Systematic characterization of the branch point binding protein, splicing factor 1, gene family in plant development and stress responses
Source: BMC Plant Biol. 2020 Aug 18;20:379. doi: 10.1186/s12870-020-02570-6 (PMC7433366; doi:10.1186/s12870-020-02570-6)
Supplement: Supplementary file 6 — Additional file 6: Table S5. List of motifs identified in the 5′-flanking regions of plant SF1s. [file 12870_2020_2570_MOESM6_ESM.pdf]

1 *S. purpurea* SapurV1A.1544s0020.1.p

\+ ARE

Site Name	Organism	Position	Strand	Matrix score.	sequence	function
ARE	Zea mays	109	-	6	AAACCA	cis-acting regulatory element essential for the anaerobic induction

\+ CCGTCC-box

Site Name	Organism	Position	Strand	Matrix score.	sequence	function
CCGTCC-box	Petroselinum hortense	390	+	6	CCGTCC	

\+ ERE

Site Name	Organism	Position	Strand	Matrix score.	sequence	function
ERE	Nicotiana glutinos	1375	-	8	ATTTCATA	

\+ LTR

Site Name	Organism	Position	Strand	Matrix score.	sequence	function
LTR	Hordeum vulgare	28	-	6	CCGAAA	cis-acting element involved in low-temperature responsiveness

2 *S. purpurea* SapurV1A.0794s0160.1.p

\+ ABRE

Site Name	Organism	Position	Strand	Matrix score.	sequence	function
ABRE	Arabidopsis thaliana	1178	-	5	ACGTG	cis-acting element involved in the abscisic acid responsiveness

\+ CGTCA-motif

Site Name	Organism	Position	Strand	Matrix score.	sequence	function
CGTCA-motif	Hordeum vulgare	1180	+	5	CGTCA	cis-acting regulatory element involved in the MeJA-responsiveness

\+ ERE

Site Name	Organism	Position	Strand	Matrix	sequence	function

score.

ERE	Nicotiana glutinos	1377	-	8	ATTTCATA
ERE	Nicotiana glutinos	248	-	8	ATTTTAAA
ERE	Nicotiana glutinos	903	-	8	ATTTTAAA
ERE	Nicotiana glutinos	792	+	8	ATTTTAAA

+ GCN4_motif

Site Name	Organism	Position	Strand	Matrix score.	sequence	function
GCN4_motif	Oryza sativa	551	-	7	TGAGTCA	cis-regulatory element involved in endosperm expression

+ TGACG-motif

Site Name	Organism	Position	Strand	Matrix score.	sequence	function
TGACG-motif	Hordeum vulgare	1180	-	5	TGACG	cis-acting regulatory element involved in the MeJA-responsiveness

3 *P. deltoides* Podel.01G135100.1.p

+ AT-rich element

Site Name	Organism	Position	Strand	Matrix score.	sequence	function
AT-rich element	Glycine max	1444	+	10	ATAGAAATCAA	binding site of AT-rich DNA binding protein (ATBP-1)

+ ERE

Site Name	Organism	Position	Strand	Matrix score.	sequence	function
ERE	Nicotiana glutinos	342	+	8	ATTTTAAA	
ERE	Nicotiana glutinos	266	-	8	ATTTTAAA	
ERE	Nicotiana glutinos	223	+	8	ATTTTAAA	

+ GCN4_motif

Site Name	Organism	Position	Strand	Matrix score.	sequence	function
GCN4_motif	Oryza sativa	798	-	7	TGAGTCA	cis-regulatory element involved in endosperm expression

+ TC-rich repeats

Site Name	Organism	Position	Strand	Matrix score.	sequence	function
TC-rich repeats	Nicotiana tabacum	1217	+	9	GTTTTCTTAC	cis-acting element involved in defense and stress responsiveness
TC-rich repeats	Nicotiana tabacum	356	+	9	GTTTTCTTAC	cis-acting element involved in defense and stress responsiveness

+ TCA-element

Site Name	Organism	Position	Strand	Matrix score.	sequence	function
TCA-element	Nicotiana tabacum	1355	+	9	CCATCTTTTT	cis-acting element involved in salicylic acid responsiveness

4 *P. trichocarpa* Potri.001G126400.2

+ AT-rich element

Site Name	Organism	Position	Strand	Matrix score.	sequence	function
AT-rich element	Glycine max	1449	+	10	ATAGAAATCAA	binding site of AT-rich DNA binding protein (ATBP-1)

+ ERE

Site Name	Organism	Position	Strand	Matrix score.	sequence	function
ERE	Nicotiana glutinos	207	-	8	ATTTTAAA	
ERE	Nicotiana glutinos	332	+	8	ATTTTAAA	

+ TC-rich repeats

Site Name	Organism	Position	Strand	Matrix score.	sequence	function
TC-rich repeats	Nicotiana tabacum	1221	+	9	GTTTTCTTAC	cis-acting element involved in defense and stress responsiveness
TC-rich repeats	Nicotiana tabacum	346	+	9	GTTTTCTTAC	cis-acting element involved in defense and stress responsiveness

+ TCA-element

Site Name	Organism	Position	Strand	Matrix score.	sequence	function
TCA-element	Nicotiana tabacum	1359	+	9	CCATCTTTTT	cis-acting element involved in salicylic acid responsiveness

+ TGA-element

Site Name	Organism	Position	Strand	Matrix score.	sequence	function
TGA-element	Brassica oleracea	343	-	6	AACGAC	auxin-responsive element

5 *P. trichocarpa* Potri.003G107200.1

\+ ABRE

Site Name	Organism	Position	Strand	Matrix score.	sequence	function
ABRE	Arabidopsis thaliana	131	+	5	ACGTG	cis-acting element involved in the abscisic acid responsiveness

]+ ARE

Site Name	Organism	Position	Strand	Matrix score.	sequence	function
ARE	Zea mays	896	-	6	AAACCA	cis-acting regulatory element essential for the anaerobic induction

\+ CAT-box

Site Name	Organism	Position	Strand	Matrix score.	sequence	function
CAT-box	Arabidopsis thaliana	40	-	6	GCCACT	cis-acting regulatory element related to meristem expression
CAT-box	Arabidopsis thaliana	1284	-	6	GCCACT	cis-acting regulatory element related to meristem expression

\+ CGTCA-motif

Site Name	Organism	Position	Strand	Matrix score.	sequence	function
CGTCA-motif	Hordeum vulgare	1397	+	5	CGTCA	cis-acting regulatory element involved in the MeJA-responsiveness

\+ ERE

Site Name	Organism	Position	Strand	Matrix score.	sequence	function
ERE	Nicotiana glutinos	1040	+	8	ATTTTAAA	
ERE	Nicotiana glutinos	1006	+	8	ATTTTAAA	
ERE	Nicotiana glutinos	1062	+	8	ATTTTAAA	

\+ GC-motif

Site Name	Organism	Position	Strand	Matrix score.	sequence	function
GC-motif	Zea mays	745	-	6	CCCCCG	enhancer-like element

involved in anoxic specific inducibility

+ LTR

Site Name	Organism	Position	Strand	Matrix score.	sequence	function
LTR	Hordeum vulgare	1239	-	6	CCGAAA	cis-acting element involved in low-temperature responsiveness

+ TC-rich repeats

Site Name	Organism	Position	Strand	Matrix score.	sequence	function
TC-rich repeats	Nicotiana tabacum	307	-	9	ATTCTCTAAC	cis-acting element involved in defense and stress responsiveness

+ TCA-element

Site Name	Organism	Position	Strand	Matrix score.	sequence	function
TCA-element	Nicotiana tabacum	1315	+	9	CCATCTTTTT	cis-acting element involved in salicylic acid responsiveness

+ TGACG-motif

Site Name	Organism	Position	Strand	Matrix score.	sequence	function
TGACG-motif	Hordeum vulgare	1397	-	5	TGACG	cis-acting regulatory element involved in the MeJA-responsiveness

6 *P. deltoides* Podel.03G113200.1.p

+ ABRE

Site Name	Organism	Position	Strand	Matrix score.	sequence	function
ABRE	Arabidopsis thaliana	232	+	5	ACGTG	cis-acting element involved in the abscisic acid responsiveness

+ CAT-box

Site Name	Organism	Position	Strand	Matrix score.	sequence	function
CAT-box	Arabidopsis thaliana	1321	-	6	GCCACT	cis-acting regulatory element related to meristem expression
CAT-box	Arabidopsis thaliana	141	-	6	GCCACT	cis-acting regulatory element related to meristem expression

+ CGTCA-motif

Site Name	Organism	Position	Strand	Matrix score.	sequence	function
CGTCA-motif	Hordeum vulgare	1023	-	5	CGTCA	cis-acting regulatory element involved in the MeJA-responsiveness
CGTCA-motif	Hordeum vulgare	1434	+	5	CGTCA	cis-acting regulatory element involved in the MeJA-responsiveness

+ ERE

Site Name	Organism	Position	Strand	Matrix score.	sequence	function
ERE	Nicotiana glutinos	1079	+	8	ATTTTAAA	
ERE	Nicotiana glutinos	1081	-	8	ATTTTAAA	

+ GC-motif

Site Name	Organism	Position	Strand	Matrix score.	sequence	function
GC-motif	Zea mays	847	-	6	CCCCCG	enhancer-like element involved in anoxic specific inducibility

+ HD-Zip 1

Site Name	Organism	Position	Strand	Matrix score.	sequence	function

HD-Zip 1	Arabidopsis thaliana	715	+	8.5	CAAT(A/T)ATTG	element involved in differentiation of the palisade mesophyll cells

+ LTR

Site Name	Organism	Position	Strand	Matrix score.	sequence	function
LTR	Hordeum vulgare	1276	-	6	CCGAAA	cis-acting element involved in low-temperature responsiveness

+ TCA-element

Site Name	Organism	Position	Strand	Matrix score.	sequence	function
TCA-element	Nicotiana tabacum	1352	+	9	CCATCTTTTT	cis-acting element involved in salicylic acid responsiveness

+ TGACG-motif

Site Name	Organism	Position	Strand	Matrix score.	sequence	function
TGACG-motif	Hordeum vulgare	1023	+	5	TGACG	cis-acting regulatory element involved in the MeJA-responsiveness
TGACG-motif	Hordeum vulgare	1434	-	5	TGACG	cis-acting regulatory element involved in the MeJA-responsiveness

7 *S. purpurea* SapurV1A.0667s0170.1.p

+ ABRE

Site Name	Organism	Position	Strand	Matrix score.	sequence	function
ABRE	Hordeum vulgare	1222	-	9	GCAACGTGTC	cis-acting element involved in the abscisic acid responsiveness
ABRE	Arabidopsis thaliana	1117	-	5	ACGTG	cis-acting element involved in the abscisic acid responsiveness
ABRE	Hordeum vulgare	1115	-	9	GCAACGTGTC	cis-acting element involved in the abscisic acid responsiveness
ABRE	Arabidopsis thaliana	1224	-	5	ACGTG	cis-acting element involved in the abscisic acid responsiveness
ABRE	Arabidopsis thaliana	676	-	7	AACCCGG	cis-acting element involved in the abscisic acid responsiveness
ABRE	Arabidopsis thaliana	24	+	5	ACGTG	cis-acting element involved in the abscisic acid responsiveness

+ CGTCA-motif

Site Name	Organism	Position	Strand	Matrix score.	sequence	function
CGTCA-motif	Hordeum vulgare	1437	+	5	CGTCA	cis-acting regulatory element involved in the MeJA-responsiveness

+ ERE

Site Name	Organism	Position	Strand	Matrix score.	sequence	function
ERE	Nicotiana glutinos	754	+	8	ATTTTAAA	

+ LTR

Site Name	Organism	Position	Strand	Matrix score.	sequence	function
LTR	Hordeum vulgare	877	+	6	CCGAAA	cis-acting element involved in low-temperature responsiveness
LTR	Hordeum vulgare	635	-	6	CCGAAA	cis-acting element

						involved in low-temperature responsiveness
LTR	Hordeum vulgare	624	-	6	CCGAAA	cis-acting element involved in low-temperature responsiveness
LTR	Hordeum vulgare	378	-	6	CCGAAA	cis-acting element involved in low-temperature responsiveness
LTR	Hordeum vulgare	854	+	6	CCGAAA	cis-acting element involved in low-temperature responsiveness

+ TGA-element

Site Name	Organism	Position	Strand	Matrix score.	sequence	function
TGA-element	Brassica oleracea	577	+	6	AACGAC	auxin-responsive element
TGA-element	Brassica oleracea	188	-	6	AACGAC	auxin-responsive element

+ TGACG-motif

Site Name	Organism	Position	Strand	Matrix score.	sequence	function
TGACG-motif	Hordeum vulgare	1437	-	5	TGACG	cis-acting regulatory element involved in the MeJA-responsiveness

8 *L. usitatissimum* Lus10038932

+ ABRE

Site Name	Organism	Position	Strand	Matrix score.	sequence	function
ABRE	Arabidopsis thaliana	940	-	5	ACGTG	cis-acting element involved in the abscisic acid responsiveness
ABRE	Arabidopsis thaliana	864	+	7	TACGGTC	cis-acting element involved in the abscisic acid responsiveness
ABRE	Arabidopsis thaliana	439	+	5	ACGTG	cis-acting element involved in the abscisic acid responsiveness
ABRE	Arabidopsis thaliana	473	-	5	ACGTG	cis-acting element involved in the abscisic acid responsiveness
ABRE	Arabidopsis thaliana	438	+	6	CACGTG	cis-acting element involved in the abscisic acid responsiveness

+ ARE

Site Name	Organism	Position	Strand	Matrix score.	sequence	function
ARE	Zea mays	362	+	6	AAACCA	cis-acting regulatory element essential for the anaerobic induction
ARE	Zea mays	1274	+	6	AAACCA	cis-acting regulatory element essential for the anaerobic induction

+ CGTCA-motif

Site Name	Organism	Position	Strand	Matrix score.	sequence	function
CGTCA-motif	Hordeum vulgare	122	-	5	CGTCA	cis-acting regulatory element involved in the MeJA-responsiveness

CGTCA-motif	Hordeum vulgare	470	+	5	CGTCA	cis-acting regulatory element involved in the MeJA-responsiveness
CGTCA-motif	Hordeum vulgare	942	+	5	CGTCA	cis-acting regulatory element involved in the MeJA-responsiveness
CGTCA-motif	Hordeum vulgare	1496	+	5	CGTCA	cis-acting regulatory element involved in the MeJA-responsiveness

\+ ERE

Site Name	Organism	Position	Strand	Matrix score.	sequence	function
ERE	Nicotiana glutinos	1166	+	8	ATTTTAAA	

\+ LTR

Site Name	Organism	Position	Strand	Matrix score.	sequence	function
LTR	Hordeum vulgare	592	-	6	CCGAAA	cis-acting element involved in low-temperature responsiveness

\+ RY-element

Site Name	Organism	Position	Strand	Matrix score.	sequence	function
RY-element	Helianthus annuus	1125	-	8	CATGCATG	cis-acting regulatory element involved in seed-specific regulation

\+ TATC-box

Site Name	Organism	Position	Strand	Matrix score.	sequence	function
TATC-box	Oryza sativa	3	-	7	TATCCCA	cis-acting element involved in gibberellin-responsiveness

\+ TGA-element

Site Name	Organism	Position	Strand	Matrix score.	sequence	function
TGA-element	Brassica oleracea	270	-	6	AACGAC	auxin-responsive element
TGA-element	Brassica oleracea	98	+	6	AACGAC	auxin-responsive element

+ TGACG-motif

Site Name	Organism	Position	Strand	Matrix score.	sequence	function
TGACG-motif	Hordeum vulgare	1496	-	5	TGACG	cis-acting regulatory element involved in the MeJA-responsiveness
TGACG-motif	Hordeum vulgare	122	+	5	TGACG	cis-acting regulatory element involved in the MeJA-responsiveness
TGACG-motif	Hordeum vulgare	470	-	5	TGACG	cis-acting regulatory element involved in the MeJA-responsiveness
TGACG-motif	Hordeum vulgare	942	-	5	TGACG	cis-acting regulatory element involved in the MeJA-responsiveness

9 *R. communis* 30147.m014250

+ ARE

Site Name	Organism	Position	Strand	Matrix score.	sequence	function
ARE	Zea mays	54	-	6	AAACCA	cis-acting regulatory element essential for the anaerobic induction
ARE	Zea mays	864	+	6	AAACCA	cis-acting regulatory element essential for the anaerobic induction
ARE	Zea mays	554	-	6	AAACCA	cis-acting regulatory element essential for the anaerobic induction

+ AT-rich sequence

Site Name	Organism	Position	Strand	Matrix score.	sequence	function
AT-rich sequence	Pisum sativum	1078	+	9	TAAAATACT	element for maximal elicitor-mediated activation (2copies)

+ CGTCA-motif

Site Name	Organism	Position	Strand	Matrix score.	sequence	function
CGTCA-motif	Hordeum vulgare	1384	-	5	CGTCA	cis-acting regulatory element involved in the MeJA-responsiveness

\+ ERE

Site Name	Organism	Position	Strand	Matrix score.	sequence	function
ERE	Nicotiana glutinos	950	+	8	ATTTCATA	
ERE	Nicotiana glutinos	1066	+	8	ATTTCATA	

\+ MBS

Site Name	Organism	Position	Strand	Matrix score.	sequence	function
MBS	Arabidopsis thaliana	498	+	6	CAACTG	MYB binding site involved in drought-inducibility
MBS	Arabidopsis thaliana	417	-	6	CAACTG	MYB binding site involved in drought-inducibility

\+ P-box

Site Name	Organism	Position	Strand	Matrix score.	sequence	function
P-box	Oryza sativa	287	+	7	CCTTTTG	gibberellin-responsive element

\+ TCA-element

Site Name	Organism	Position	Strand	Matrix score.	sequence	function
TCA-element	Brassica oleracea	136	+	9	TCAGAAGAGG	cis-acting element involved in salicylic acid responsiveness

\+ TGACG-motif

Site Name	Organism	Position	Strand	Matrix score.	sequence	function
TGACG-motif	Hordeum vulgare	1384	+	5	TGACG	cis-acting regulatory element involved in the MeJA-responsiveness

10 *M. esculenta* **Manes.06G040900.1.p**

\+ ABRE

Site Name	Organism	Position	Strand	Matrix score.	sequence	function
ABRE	Arabidopsis thaliana	39	+	5	ACGTG	cis-acting element involved in the abscisic acid responsiveness
ABRE	Arabidopsis thaliana	38	+	6	CACGTG	cis-acting element involved in the abscisic acid responsiveness

\+ ARE

Site Name	Organism	Position	Strand	Matrix score.	sequence	function
ARE	Zea mays	377	+	6	AAACCA	cis-acting regulatory element essential for the anaerobic induction
ARE	Zea mays	273	+	6	AAACCA	cis-acting regulatory element essential for the anaerobic induction

\+ CGTCA-motif

Site Name	Organism	Position	Strand	Matrix score.	sequence	function
CGTCA-motif	Hordeum vulgare	898	-	5	CGTCA	cis-acting regulatory element involved in the MeJA-responsiveness

\+ ERE

Site Name	Organism	Position	Strand	Matrix score.	sequence	function
ERE	Nicotiana glutinos	1273	+	8	ATTTTAAA	
ERE	Nicotiana glutinos	1266	+	8	ATTTTAAA	
ERE	Nicotiana glutinos	1199	+	8	ATTTTAAA	
ERE	Nicotiana glutinos	1251	+	8	ATTTTAAA	
ERE	Nicotiana glutinos	633	-	8	ATTTTAAA	
ERE	Nicotiana glutinos	484	-	8	ATTTTAAA	
ERE	Nicotiana glutinos	460	-	8	ATTTTAAA	
ERE	Nicotiana glutinos	1253	-	8	ATTTTAAA	

ERE	Nicotiana glutinos	666	+	8	ATTTCATA	

+ MBS

Site Name	Organism	Position	Strand	Matrix score.	sequence	function
MBS	Arabidopsis thaliana	863	-	6	CAACTG	MYB binding site involved in drought-inducibility
MBS	Arabidopsis thaliana	512	+	6	CAACTG	MYB binding site involved in drought-inducibility

+ TCA-element

Site Name	Organism	Position	Strand	Matrix score.	sequence	function
TCA-element	Nicotiana tabacum	408	+	10	CCATCTTTTT	cis-acting element involved in salicylic acid responsiveness

+ TGACG-motif

Site Name	Organism	Position	Strand	Matrix score.	sequence	function
TGACG-motif	Hordeum vulgare	898	+	5	TGACG	cis-acting regulatory element involved in the MeJA-responsiveness

+ WUN-motif

Site Name	Organism	Position	Strand	Matrix score.	sequence	function
WUN-motif	Nicotiana glutinosa	1328	-	8	AAATTACT	

11 *C. sinensis* orange1.1g040243m

+ ABRE

Site Name	Organism	Position	Strand	Matrix score.	sequence	function
ABRE	Arabidopsis thaliana	1060	+	5	ACGTG	cis-acting element involved in the abscisic acid responsiveness
ABRE	Arabidopsis thaliana	1001	-	5	ACGTG	cis-acting element involved in the abscisic acid responsiveness
ABRE	Arabidopsis thaliana	1059	-	6	CACGTG	cis-acting element involved in the abscisic acid responsiveness

+ CGTCA-motif

Site Name	Organism	Position	Strand	Matrix score.	sequence	function
CGTCA-motif	Hordeum vulgare	298	-	5	CGTCA	cis-acting regulatory element involved in the MeJA-responsiveness
CGTCA-motif	Hordeum vulgare	1479	+	5	CGTCA	cis-acting regulatory element involved in the MeJA-responsiveness
CGTCA-motif	Hordeum vulgare	1003	+	5	CGTCA	cis-acting regulatory element involved in the MeJA-responsiveness
CGTCA-motif	Hordeum vulgare	1123	+	5	CGTCA	cis-acting regulatory element involved in the MeJA-responsiveness

+ ERE

Site Name	Organism	Position	Strand	Matrix score.	sequence	function
ERE	Nicotiana glutinos	123	-	8	ATTTCATA	

+ GCN4_motif

Site Name	Organism	Position	Strand	Matrix score.	sequence	function
GCN4_motif	Oryza sativa	483	-	7	TGAGTCA	cis-regulatory element involved in endosperm expression
GCN4_motif	Oryza sativa	353	-	7	TGAGTCA	cis-regulatory element

						involved in endosperm expression

+ MBS

Site Name	Organism	Position	Strand	Matrix score.	sequence	function
MBS	Arabidopsis thaliana	1335	-	6	CAACTG	MYB binding site involved in drought-inducibility

+ TGACG-motif

Site Name	Organism	Position	Strand	Matrix score.	sequence	function
TGACG-motif	Hordeum vulgare	298	+	5	TGACG	cis-acting regulatory element involved in the MeJA-responsiveness
TGACG-motif	Hordeum vulgare	1123	-	5	TGACG	cis-acting regulatory element involved in the MeJA-responsiveness
TGACG-motif	Hordeum vulgare	1479	-	5	TGACG	cis-acting regulatory element involved in the MeJA-responsiveness
TGACG-motif	Hordeum vulgare	1003	-	5	TGACG	cis-acting regulatory element involved in the MeJA-responsiveness

12 *C. clementina* Ciclev10004301m

+ ABRE

Site Name	Organism	Position	Strand	Matrix score.	sequence	function
ABRE	Arabidopsis thaliana	1345	-	5	ACGTG	cis-acting element involved in the abscisic acid responsiveness
ABRE	Arabidopsis thaliana	1404	+	5	ACGTG	cis-acting element involved in the abscisic acid responsiveness
ABRE	Arabidopsis thaliana	1403	-	6	CACGTG	cis-acting element involved in the abscisic acid responsiveness

+ CCGTCC-box

Site Name	Organism	Position	Strand	Matrix score.	sequence	function
CCGTCC-box	Petroselinum hortense	528	-	6	CCGTCC	

+ CGTCA-motif

Site Name	Organism	Position	Strand	Matrix score.	sequence	function
CGTCA-motif	Hordeum vulgare	1467	+	5	CGTCA	cis-acting regulatory element involved in the MeJA-responsiveness
CGTCA-motif	Hordeum vulgare	1347	+	5	CGTCA	cis-acting regulatory element involved in the MeJA-responsiveness
CGTCA-motif	Hordeum vulgare	642	-	5	CGTCA	cis-acting regulatory element involved in the MeJA-responsiveness

+ ERE

Site Name	Organism	Position	Strand	Matrix score.	sequence	function
ERE	Nicotiana glutinos	468	-	8	ATTTCATA	

+ GCN4_motif

Site Name	Organism	Position	Strand	Matrix score.	sequence	function
GCN4_motif	Oryza sativa	697	-	7	TGAGTCA	cis-regulatory element involved in endosperm expression

GCN4_motif	Oryza sativa	827	-	7	TGAGTCA	cis-regulatory element involved in endosperm expression

+ MBS

Site Name	Organism	Position	Strand	Matrix score.	sequence	function
MBS	Arabidopsis thaliana	273	+	6	CAACTG	MYB binding site involved in drought-inducibility

+ TC-rich repeats

Site Name	Organism	Position	Strand	Matrix score.	sequence	function
TC-rich repeats	Nicotiana tabacum	512	-	9	ATTCTCTAAC	cis-acting element involved in defense and stress responsiveness

+ TGACG-motif

Site Name	Organism	Position	Strand	Matrix score.	sequence	function
TGACG-motif	Hordeum vulgare	642	+	5	TGACG	cis-acting regulatory element involved in the MeJA-responsiveness
TGACG-motif	Hordeum vulgare	1347	-	5	TGACG	cis-acting regulatory element involved in the MeJA-responsiveness
TGACG-motif	Hordeum vulgare	1467	-	5	TGACG	cis-acting regulatory element involved in the MeJA-responsiveness

13 *A. occidentale* Anaoc.0018s0425.1.p

+ ARE

Site Name	Organism	Position	Strand	Matrix score.	sequence	function
ARE	Zea mays	989	+	6	AAACCA	cis-acting regulatory element essential for the anaerobic induction

+ CGTCA-motif

Site Name	Organism	Position	Strand	Matrix score.	sequence	function
CGTCA-motif	Hordeum vulgare	1094	+	5	CGTCA	cis-acting regulatory element involved in the MeJA-responsiveness

+ ERE

Site Name	Organism	Position	Strand	Matrix score.	sequence	function
ERE	Nicotiana glutinos	1268	-	8	ATTTTAAA	
ERE	Nicotiana glutinos	102	+	8	ATTTTAAA	
ERE	Nicotiana glutinos	1216	+	8	ATTTTAAA	
ERE	Nicotiana glutinos	209	-	8	ATTTTAAA	
ERE	Nicotiana glutinos	207	+	8	ATTTTAAA	
ERE	Nicotiana glutinos	1376	+	8	ATTTTAAA	
ERE	Nicotiana glutinos	1218	-	8	ATTTTAAA	
ERE	Nicotiana glutinos	1378	-	8	ATTTTAAA	

+ MBS

Site Name	Organism	Position	Strand	Matrix score.	sequence	function
MBS	Arabidopsis thaliana	697	-	6	CAACTG	MYB binding site involved in drought-inducibility

+ P-box

Site Name	Organism	Position	Strand	Matrix score.	sequence	function
P-box	Oryza sativa	1435	+	7	CCTTTTG	gibberellin-responsive element

+ TCA-element

Site Name	Organism	Position	Strand	Matrix score.	sequence	function
TCA-element	Nicotiana tabacum	1286	-	9	CCATCTTTTT	cis-acting element involved in salicylic

						acid responsiveness
TCA-element	Nicotiana tabacum	1133	-	9	CCATCTTTTT	cis-acting element involved in salicylic acid responsiveness

+ TGACG-motif

Site Name	Organism	Position	Strand	Matrix score.	sequence	function
TGACG-motif	Hordeum vulgare	1094	-	5	TGACG	cis-acting regulatory element involved in the MeJA-responsiveness

14 A. occidentale Anaoc.0010s0343.1.p

+ ARE

Site Name	Organism	Position	Strand	Matrix score.	sequence	function
ARE	Zea mays	350	-	6	AAACCA	cis-acting regulatory element essential for the anaerobic induction

+ ERE

Site Name	Organism	Position	Strand	Matrix score.	sequence	function
ERE	Nicotiana glutinos	845	+	8	ATTTCATA	
ERE	Nicotiana glutinos	1133	-	8	ATTTTAAA	

+ P-box

Site Name	Organism	Position	Strand	Matrix score.	sequence	function
P-box	Oryza sativa	74	-	7	CCTTTTG	gibberellin-responsive element

+ TC-rich repeats

Site Name	Organism	Position	Strand	Matrix score.	sequence	function
TC-rich repeats	Nicotiana tabacum	1181	-	9	GTTTTCTTAC	cis-acting element involved in defense and stress responsiveness
TC-rich repeats	Nicotiana tabacum	1067	+	9	ATTCTCTAAC	cis-acting element involved in defense and stress responsiveness
TC-rich repeats	Nicotiana tabacum	745	+	10	ATTCTCTAAC	cis-acting element involved in defense and stress responsiveness

15 *G. hirsutum* Gohir.D09G250000.1.p

+ AT-rich element

Site Name	Organism	Position	Strand	Matrix score.	sequence	function
AT-rich element	Glycine max	1284	-	10	ATAGAAATCAA	binding site of AT-rich DNA binding protein (ATBP-1)

+ ERE

Site Name	Organism	Position	Strand	Matrix score.	sequence	function
ERE	Nicotiana glutinos	700	-	8	ATTTTAAA	
ERE	Nicotiana glutinos	412	-	8	ATTTTAAA	
ERE	Nicotiana glutinos	333	-	8	ATTTTAAA	
ERE	Nicotiana glutinos	331	+	8	ATTTTAAA	

+ MBS

Site Name	Organism	Position	Strand	Matrix score.	sequence	function
MBS	Arabidopsis thaliana	1307	+	6	CAACTG	MYB binding site involved in drought-inducibility

+ P-box

Site Name	Organism	Position	Strand	Matrix score.	sequence	function
P-box	Oryza sativa	791	-	7	CCTTTTG	gibberellin-responsive element

+ TC-rich repeats

Site Name	Organism	Position	Strand	Matrix score.	sequence	function
TC-rich repeats	Nicotiana tabacum	1203	+	9	ATTCTCTAAC	cis-acting element involved in defense and stress responsiveness

+ WUN-motif

Site Name	Organism	Position	Strand	Matrix score.	sequence	function
WUN-motif	Nicotiana glutinosa	159	-	8	AAATTACT	

16 *G. raimondii* Gorai.006G274400.1

+ AT-rich element

Site Name	Organism	Position	Strand	Matrix score.	sequence	function
AT-rich element	Glycine max	1225	-	10	ATAGAAATCAA	binding site of AT-rich DNA binding protein (ATBP-1)

+ ERE

Site Name	Organism	Position	Strand	Matrix score.	sequence	function
ERE	Nicotiana glutinos	248	+	8	ATTTTAAA	
ERE	Nicotiana glutinos	329	-	8	ATTTTAAA	
ERE	Nicotiana glutinos	250	-	8	ATTTTAAA	
ERE	Nicotiana glutinos	640	-	8	ATTTTAAA	

+ GC-motif

Site Name	Organism	Position	Strand	Matrix score.	sequence	function
GC-motif	Zea mays	1098	+	6	CCCCCG	enhancer-like element involved in anoxic specific inducibility

+ MBS

Site Name	Organism	Position	Strand	Matrix score.	sequence	function
MBS	Arabidopsis thaliana	1248	+	6	CAACTG	MYB binding site involved in drought-inducibility

+ P-box

Site Name	Organism	Position	Strand	Matrix score.	sequence	function
P-box	Oryza sativa	731	-	7	CCTTTTG	gibberellin-responsive element

+ TC-rich repeats

Site Name	Organism	Position	Strand	Matrix score.	sequence	function
TC-rich repeats	Nicotiana tabacum	1144	+	9	ATTCTCTAAC	cis-acting element involved in defense and stress responsiveness

+ WUN-motif

Site Name	Organism	Position	Strand	Matrix score.	sequence	function
WUN-motif	Nicotiana glutinosa	78	-	8	AAATTACT	
WUN-motif	Nicotiana glutinosa	776	-	9	AAATTACTA	
WUN-motif	Nicotiana glutinosa	777	-	8	AAATTACT	

17 *G. hirsutum* Gohir.1Z080100.1.p

\+ ARE

Site Name	Organism	Position	Strand	Matrix score.	sequence	function
ARE	Zea mays	754	-	6	AAACCA	cis-acting regulatory element essential for the anaerobic induction

\+ CAT-box

Site Name	Organism	Position	Strand	Matrix score.	sequence	function
CAT-box	Arabidopsis thaliana	1354	-	6	GCCACT	cis-acting regulatory element related to meristem expression

\+ ERE

Site Name	Organism	Position	Strand	Matrix score.	sequence	function
ERE	Nicotiana glutinos	1010	+	8	ATTTTAAA	
ERE	Nicotiana glutinos	887	+	8	ATTTTAAA	
ERE	Nicotiana glutinos	773	-	8	ATTTTAAA	
ERE	Nicotiana glutinos	600	-	8	ATTTTAAA	
ERE	Nicotiana glutinos	628	+	8	ATTTTAAA	

\+ GC-motif

Site Name	Organism	Position	Strand	Matrix score.	sequence	function
GC-motif	Zea mays	1279	+	6	CCCCCG	enhancer-like element involved in anoxic specific inducibility

\+ GCN4_motif

Site Name	Organism	Position	Strand	Matrix score.	sequence	function
GCN4_motif	Oryza sativa	880	-	7	TGAGTCA	cis-regulatory element involved in endosperm expression

\+ P-box

Site Name	Organism	Position	Strand	Matrix score.	sequence	function
P-box	Oryza sativa	990	-	7	CCTTTTG	gibberellin-responsive element

\+ WUN-motif

Site Name	Organism	Position	Strand	Matrix score.	sequence	function
WUN-motif	Nicotiana glutinosa	350	-	8	AAATTACT	
WUN-motif	Nicotiana glutinosa	16	+	9	AAATTTCTT	
WUN-motif	Nicotiana glutinosa	1035	-	9	AAATTACTA	
WUN-motif	Nicotiana glutinosa	1036	-	8	AAATTACT	

18 T. cacao Thecc1EG014895t1

+ ABRE

Site Name	Organism	Position	Strand	Matrix score.	sequence	function
ABRE	Arabidopsis thaliana	244	+	5	ACGTG	cis-acting element involved in the abscisic acid responsiveness

+ CAT-box

Site Name	Organism	Position	Strand	Matrix score.	sequence	function
CAT-box	Arabidopsis thaliana	559	-	6	GCCACT	cis-acting regulatory element related to meristem expression

+ CCGTCC-box

Site Name	Organism	Position	Strand	Matrix score.	sequence	function
CCGTCC-box	Petroselinum hortense	458	-	6	CCGTCC	
CCGTCC-box	Petroselinum hortense	274	-	6	CCGTCC	

+ CGTCA-motif

Site Name	Organism	Position	Strand	Matrix score.	sequence	function
CGTCA-motif	Hordeum vulgare	1205	+	5	CGTCA	cis-acting regulatory element involved in the MeJA-responsiveness
CGTCA-motif	Hordeum vulgare	592	-	5	CGTCA	cis-acting regulatory element involved in the MeJA-responsiveness

+ ERE

Site Name	Organism	Position	Strand	Matrix score.	sequence	function
ERE	Nicotiana glutinos	1345	+	8	ATTTTAAA	

+ GCN4_motif

Site Name	Organism	Position	Strand	Matrix score.	sequence	function
GCN4_motif	Oryza sativa	305	-	7	TGAGTCA	cis-regulatory element involved in endosperm expression

+ MBS

Site Name	Organism	Position	Strand	Matrix score.	sequence	function

MBS	Arabidopsis thaliana	99	-	6	CAACTG	MYB binding site involved in drought-inducibility
MBS	Arabidopsis thaliana	588	-	6	CAACTG	MYB binding site involved in drought-inducibility

+ TATC-box

Site Name	Organism	Position	Strand	Matrix score.	sequence	function
TATC-box	Oryza sativa	641	-	7	TATCCCA	cis-acting element involved in gibberellin-responsiveness

+ TGACG-motif

Site Name	Organism	Position	Strand	Matrix score.	sequence	function
TGACG-motif	Hordeum vulgare	592	+	5	TGACG	cis-acting regulatory element involved in the MeJA-responsiveness
TGACG-motif	Hordeum vulgare	1205	-	5	TGACG	cis-acting regulatory element involved in the MeJA-responsiveness

+ dOCT

Site Name	Organism	Position	Strand	Matrix score.	sequence	function
dOCT	Zea mays	332	+	8	CTCGGATC	

19 *G. max* Glyma.09G230800.1.p

+ ABRE

Site Name	Organism	Position	Strand	Matrix score.	sequence	function
ABRE	Arabidopsis thaliana	159	+	5	ACGTG	cis-acting element involved in the abscisic acid responsiveness
ABRE	Arabidopsis thaliana	1418	-	7	AACCCGG	cis-acting element involved in the abscisic acid responsiveness
ABRE	Arabidopsis thaliana	158	+	6	CACGTG	cis-acting element involved in the abscisic acid responsiveness
ABRE	Arabidopsis thaliana	348	-	5	ACGTG	cis-acting element involved in the abscisic acid responsiveness

+ ARE

Site Name	Organism	Position	Strand	Matrix score.	sequence	function
ARE	Zea mays	1340	+	6	AAACCA	cis-acting regulatory element essential for the anaerobic induction
ARE	Zea mays	25	+	6	AAACCA	cis-acting regulatory element essential for the anaerobic induction

+ AT-rich element

Site Name	Organism	Position	Strand	Matrix score.	sequence	function
AT-rich element	Glycine max	1053	+	10	ATAGAAATCAA	binding site of AT-rich DNA binding protein (ATBP-1)

+ CGTCA-motif

Site Name	Organism	Position	Strand	Matrix score.	sequence	function
CGTCA-motif	Hordeum vulgare	113	-	5	CGTCA	cis-acting regulatory

element involved in the MeJA-responsiveness

+ ERE

Site Name	Organism	Position	Strand	Matrix score.	sequence	function
ERE	Nicotiana glutinos	1301	-	8	ATTTTAAA	
ERE	Nicotiana glutinos	388	-	8	ATTTTAAA	
ERE	Nicotiana glutinos	639	-	8	ATTTTAAA	

+ MBS

Site Name	Organism	Position	Strand	Matrix score.	sequence	function
MBS	Arabidopsis thaliana	382	-	6	CAACTG	MYB binding site involved in drought-inducibility

+ TCA-element

Site Name	Organism	Position	Strand	Matrix score.	sequence	function
TCA-element	Nicotiana tabacum	676	-	9	CCATCTTTTT	cis-acting element involved in salicylic acid responsiveness

+ TGA-element

Site Name	Organism	Position	Strand	Matrix score.	sequence	function
TGA-element	Brassica oleracea	512	-	6	AACGAC	auxin-responsive element

+ TGACG-motif

Site Name	Organism	Position	Strand	Matrix score.	sequence	function
TGACG-motif	Hordeum vulgare	113	+	5	TGACG	cis-acting regulatory element involved in the MeJA-responsiveness

+ WUN-motif

Site Name	Organism	Position	Strand	Matrix score.	sequence	function
WUN-motif	Nicotiana glutinosa	1043	-	8	AAATTACT	
WUN-motif	Nicotiana glutinosa	1042	-	9	AAATTACTA	
WUN-motif	Nicotiana glutinosa	1139	-	9	AAATTTCTT	
WUN-motif	Nicotiana glutinosa	469	+	8	AAATTACT	
WUN-motif	Nicotiana glutinosa	982	+	9	AAATTTCTT	

20 *G. max* Glyma.12G005900.1.p

+ ABRE

Site Name	Organism	Position	Strand	Matrix score.	sequence	function
ABRE	Arabidopsis thaliana	1396	-	7	AACCCGG	cis-acting element involved in the abscisic acid responsiveness
ABRE	Arabidopsis thaliana	1471	+	5	ACGTG	cis-acting element involved in the abscisic acid responsiveness

+ AT-rich sequence

Site Name	Organism	Position	Strand	Matrix score.	sequence	function
AT-rich sequence	Pisum sativum	1195	+	9	TAAAATACT	element for maximal elicitor-mediated activation (2copies)

+ ERE

Site Name	Organism	Position	Strand	Matrix score.	sequence	function
ERE	Nicotiana glutinos	1177	+	8	ATTTCATA	
ERE	Nicotiana glutinos	973	-	8	ATTTCATA	
ERE	Nicotiana glutinos	787	-	8	ATTTCATA	

+ LTR

Site Name	Organism	Position	Strand	Matrix score.	sequence	function
LTR	Hordeum vulgare	1326	+	6	CCGAAA	cis-acting element involved in low-temperature responsiveness

+ MBS

Site Name	Organism	Position	Strand	Matrix score.	sequence	function
MBS	Arabidopsis thaliana	56	+	6	CAACTG	MYB binding site involved in drought-inducibility

21 *P. vulgaris* Phvul.011G006700.1.p

+ ABRE

Site Name	Organism	Position	Strand	Matrix score.	sequence	function
ABRE	Arabidopsis thaliana	1484	+	5	ACGTG	cis-acting element involved in the abscisic acid responsiveness
ABRE	Arabidopsis thaliana	1483	-	6	CACGTG	cis-acting element involved in the abscisic acid responsiveness

+ CGTCA-motif

Site Name	Organism	Position	Strand	Matrix score.	sequence	function
CGTCA-motif	Hordeum vulgare	1480	+	5	CGTCA	cis-acting regulatory element involved in the MeJA-responsiveness

+ ERE

Site Name	Organism	Position	Strand	Matrix score.	sequence	function
ERE	Nicotiana glutinos	1168	-	8	ATTTTAAA	
ERE	Nicotiana glutinos	1350	-	8	ATTTTAAA	

+ TCA-element

Site Name	Organism	Position	Strand	Matrix score.	sequence	function
TCA-element	Nicotiana tabacum	123	-	9	CCATCTTTTT	cis-acting element involved in salicylic acid responsiveness

+ TGACG-motif

Site Name	Organism	Position	Strand	Matrix score.	sequence	function
TGACG-motif	Hordeum vulgare	1480	-	5	TGACG	cis-acting regulatory element involved in the MeJA-responsiveness

22 *T. pratense* Tp57577_TGAC_v2_mRNA3767

+ ABRE

Site Name	Organism	Position	Strand	Matrix score.	sequence	function
ABRE	Arabidopsis thaliana	695	-	5	ACGTG	cis-acting element involved in the abscisic acid responsiveness

+ CGTCA-motif

Site Name	Organism	Position	Strand	Matrix score.	sequence	function
CGTCA-motif	Hordeum vulgare	1080	-	5	CGTCA	cis-acting regulatory element involved in the MeJA-responsiveness

+ ERE

Site Name	Organism	Position	Strand	Matrix score.	sequence	function
ERE	Nicotiana glutinos	433	+	8	ATTTTAAA	
ERE	Nicotiana glutinos	270	-	8	ATTTTAAA	
ERE	Nicotiana glutinos	24	-	8	ATTTTAAA	
ERE	Nicotiana glutinos	22	+	8	ATTTTAAA	
ERE	Nicotiana glutinos	425	-	8	ATTTCATA	

+ GC-motif

Site Name	Organism	Position	Strand	Matrix score.	sequence	function
GC-motif	Zea mays	613	-	6	CCCCCG	enhancer-like element involved in anoxic specific inducibility

+ TGACG-motif

Site Name	Organism	Position	Strand	Matrix score.	sequence	function
TGACG-motif	Hordeum vulgare	1080	+	5	TGACG	cis-acting regulatory element involved in the MeJA-responsiveness

+ dOCT

Site Name	Organism	Position	Strand	Matrix score.	sequence	function
dOCT	Zea mays	1282	+	8	CACGGATC	

23 *M. truncatula* Medtr4g036415.1

+ ABRE

Site Name	Organism	Position	Strand	Matrix score.	sequence	function
ABRE	Arabidopsis thaliana	1413	+	5	ACGTG	cis-acting element involved in the abscisic acid responsiveness
ABRE	Arabidopsis thaliana	1006	-	5	ACGTG	cis-acting element involved in the abscisic acid responsiveness
ABRE	Arabidopsis thaliana	189	-	5	ACGTG	cis-acting element involved in the abscisic acid responsiveness
ABRE	Arabidopsis thaliana	915	-	7	AACCCGG	cis-acting element involved in the abscisic acid responsiveness

+ ARE

Site Name	Organism	Position	Strand	Matrix score.	sequence	function
ARE	Zea mays	157	+	6	AAACCA	cis-acting regulatory element essential for the anaerobic induction
ARE	Zea mays	317	+	6	AAACCA	cis-acting regulatory element essential for the anaerobic induction

+ CGTCA-motif

Site Name	Organism	Position	Strand	Matrix score.	sequence	function
CGTCA-motif	Hordeum vulgare	1481	+	5	CGTCA	cis-acting regulatory element involved in the MeJA-responsiveness

+ LTR

Site Name	Organism	Position	Strand	Matrix score.	sequence	function
LTR	Hordeum vulgare	49	-	6	CCGAAA	cis-acting element

						involved in low-temperature responsiveness
LTR	Hordeum vulgare	1161	+	6	CCGAAA	cis-acting element involved in low-temperature responsiveness

+ TGA-element

Site Name	Organism	Position	Strand	Matrix score.	sequence	function
TGA-element	Brassica oleracea	287	+	6	AACGAC	auxin-responsive element
TGA-element	Brassica oleracea	88	+	6	AACGAC	auxin-responsive element

+ TGACG-motif

Site Name	Organism	Position	Strand	Matrix score.	sequence	function
TGACG-motif	Hordeum vulgare	1481	-	5	TGACG	cis-acting regulatory element involved in the MeJA-responsiveness

+ WUN-motif

Site Name	Organism	Position	Strand	Matrix score.	sequence	function
WUN-motif	Brassica oleracea	127	-	9	AAATTTCCT	wound-responsive element

+ dOCT

Site Name	Organism	Position	Strand	Matrix score.	sequence	function
dOCT	Zea mays	907	-	8	CTCGGATC	

24 *C. sativus* Cucsa.094220.1

\+ ABRE

Site Name	Organism	Position	Strand	Matrix score.	sequence	function
ABRE	Arabidopsis thaliana	664	-	5	ACGTG	cis-acting element involved in the abscisic acid responsiveness

\+ ARE

Site Name	Organism	Position	Strand	Matrix score.	sequence	function
ARE	Zea mays	417	+	6	AAACCA	cis-acting regulatory element essential for the anaerobic induction

\+ AT-rich element

Site Name	Organism	Position	Strand	Matrix score.	sequence	function
AT-rich element	Glycine max	175	-	10	ATAGAAATCAA	binding site of AT-rich DNA binding protein (ATBP-1)

\+ AuxRE

Site Name	Organism	Position	Strand	Matrix score.	sequence	function
AuxRE	Glycine max	693	-	11	TGTCTCAATAAG	part of an auxin-responsive element

\+ CAT-box

Site Name	Organism	Position	Strand	Matrix score.	sequence	function
CAT-box	Arabidopsis thaliana	20	-	6	GCCACT	cis-acting regulatory element related to meristem expression

\+ P-box

Site Name	Organism	Position	Strand	Matrix score.	sequence	function
P-box	Oryza sativa	712	+	7	CCTTTTG	gibberellin-responsive element

\+ WUN-motif

Site Name	Organism	Position	Strand	Matrix score.	sequence	function
WUN-motif	Nicotiana glutinosa	946	-	9	TAATTACTC	
WUN-motif	Nicotiana glutinosa	1196	+	8	AAATTACT	

25 *M. domestica* MDP0000558834

+ ABRE

Site Name	Organism	Position	Strand	Matrix score.	sequence	function
ABRE	Hordeum vulgare	208	+	9	CGCACGTGTC	cis-acting element involved in the abscisic acid responsiveness
ABRE	Arabidopsis thaliana	1175	+	5	ACGTG	cis-acting element involved in the abscisic acid responsiveness
ABRE	Arabidopsis thaliana	210	-	5	ACGTG	cis-acting element involved in the abscisic acid responsiveness
ABRE	Arabidopsis thaliana	1282	-	5	ACGTG	cis-acting element involved in the abscisic acid responsiveness

+ ARE

Site Name	Organism	Position	Strand	Matrix score.	sequence	function
ARE	Zea mays	136	+	6	AAACCA	cis-acting regulatory element essential for the anaerobic induction
ARE	Zea mays	1178	-	6	AAACCA	cis-acting regulatory element essential for the anaerobic induction

+ CGTCA-motif

Site Name	Organism	Position	Strand	Matrix score.	sequence	function
CGTCA-motif	Hordeum vulgare	474	+	5	CGTCA	cis-acting regulatory element involved in the MeJA-responsiveness
CGTCA-motif	Hordeum vulgare	685	+	5	CGTCA	cis-acting regulatory element involved in the MeJA-responsiveness

+ LTR

Site Name	Organism	Position	Strand	Matrix score.	sequence	function
LTR	Hordeum vulgare	5	+	6	CCGAAA	cis-acting element involved in low-temperature responsiveness

+ MBS

Site Name	Organism	Position	Strand	Matrix score.	sequence	function
MBS	Arabidopsis thaliana	360	+	6	CAACTG	MYB binding site involved in drought-inducibility

+ TC-rich repeats

Site Name	Organism	Position	Strand	Matrix score.	sequence	function
TC-rich repeats	Nicotiana tabacum	343	+	9	ATTCTCTAAC	cis-acting element involved in defense and stress responsiveness
TC-rich repeats	Nicotiana tabacum	509	+	9	GTTTTCTTAC	cis-acting element involved in defense and stress responsiveness

+ TGACG-motif

Site Name	Organism	Position	Strand	Matrix score.	sequence	function
TGACG-motif	Hordeum vulgare	685	-	5	TGACG	cis-acting regulatory element involved in the MeJA-responsiveness
TGACG-motif	Hordeum vulgare	474	-	5	TGACG	cis-acting regulatory element involved in the MeJA-responsiveness

26 *M. domestica* MDP0000832994

\+ ARE

Site Name	Organism	Position	Strand	Matrix score.	sequence	function
ARE	Zea mays	612	-	6	AAACCA	cis-acting regulatory element essential for the anaerobic induction

\+ ERE

Site Name	Organism	Position	Strand	Matrix score.	sequence	function
ERE	Nicotiana glutinos	122	-	8	ATTTTAAA	
ERE	Nicotiana glutinos	1047	+	8	ATTTTAAA	

\+ LTR

Site Name	Organism	Position	Strand	Matrix score.	sequence	function
LTR	Hordeum vulgare	311	+	6	CCGAAA	cis-acting element involved in low-temperature responsiveness

\+ P-box

Site Name	Organism	Position	Strand	Matrix score.	sequence	function
P-box	Oryza sativa	348	-	7	CCTTTTG	gibberellin-responsive element

\+ TGA-element

Site Name	Organism	Position	Strand	Matrix score.	sequence	function
TGA-element	Brassica oleracea	145	-	6	AACGAC	auxin-responsive element
TGA-element	Brassica oleracea	1334	-	6	AACGAC	auxin-responsive element

27 *P. persica* Prupe.2G275100.1.p

+ ABRE

Site Name	Organism	Position	Strand	Matrix score.	sequence	function
ABRE	Arabidopsis thaliana	501	+	5	ACGTG	cis-acting element involved in the abscisic acid responsiveness
ABRE	Arabidopsis thaliana	500	+	6	CACGTG	cis-acting element involved in the abscisic acid responsiveness
ABRE	Arabidopsis thaliana	415	+	5	ACGTG	cis-acting element involved in the abscisic acid responsiveness

+ ARE

Site Name	Organism	Position	Strand	Matrix score.	sequence	function
ARE	Zea mays	1252	+	6	AAACCA	cis-acting regulatory element essential for the anaerobic induction
ARE	Zea mays	1492	+	6	AAACCA	cis-acting regulatory element essential for the anaerobic induction

+ AuxRR-core

Site Name	Organism	Position	Strand	Matrix score.	sequence	function
AuxRR-core	Nicotiana tabacum	432	-	7	GGTCCAT	cis-acting regulatory element involved in auxin responsiveness

+ GC-motif

Site Name	Organism	Position	Strand	Matrix score.	sequence	function
GC-motif	Zea mays	1426	+	6	CCCCCG	enhancer-like element involved in anoxic specific inducibility

+ GCN4_motif

Site Name	Organism	Position	Strand	Matrix score.	sequence	function
GCN4_motif	Oryza sativa	346	+	7	TGAGTCA	cis-regulatory element involved in endosperm expression

+ LTR

Site Name	Organism	Position	Strand	Matrix score.	sequence	function
LTR	Hordeum vulgare	937	-	6	CCGAAA	cis-acting element involved in low-temperature responsiveness

+ TC-rich repeats

Site Name	Organism	Position	Strand	Matrix score.	sequence	function
TC-rich repeats	Nicotiana tabacum	96	+	9	GTTTTCTTAC	cis-acting element involved in defense and stress responsiveness

+ TGA-element

Site Name	Organism	Position	Strand	Matrix score.	sequence	function
TGA-element	Brassica oleracea	1303	-	6	AACGAC	auxin-responsive element
TGA-element	Brassica oleracea	1346	-	6	AACGAC	auxin-responsive element

+ WUN-motif

Site Name	Organism	Position	Strand	Matrix score.	sequence	function
WUN-motif	Nicotiana glutinosa	1181	+	9	TTATTACAT	
WUN-motif	Nicotiana glutinosa	1009	+	8	AAATTACT	
WUN-motif	Nicotiana glutinosa	712	+	9	CCATTTCAA	

28 *F. vesca* mrna21192.1-v1.0-hybrid

\+ ABRE

Site Name	Organism	Position	Strand	Matrix score.	sequence	function
ABRE	Arabidopsis thaliana	819	-	5	ACGTG	cis-acting element involved in the abscisic acid responsiveness
ABRE	Arabidopsis thaliana	809	-	5	ACGTG	cis-acting element involved in the abscisic acid responsiveness
ABRE	Arabidopsis thaliana	384	-	5	ACGTG	cis-acting element involved in the abscisic acid responsiveness
ABRE	Arabidopsis thaliana	703	+	5	ACGTG	cis-acting element involved in the abscisic acid responsiveness

\+ ARE

Site Name	Organism	Position	Strand	Matrix score.	sequence	function
ARE	Zea mays	70	-	6	AAACCA	cis-acting regulatory element essential for the anaerobic induction

\+ CGTCA-motif

Site Name	Organism	Position	Strand	Matrix score.	sequence	function
CGTCA-motif	Hordeum vulgare	791	+	5	CGTCA	cis-acting regulatory element involved in the MeJA-responsiveness

\+ LTR

Site Name	Organism	Position	Strand	Matrix score.	sequence	function
LTR	Hordeum vulgare	247	+	6	CCGAAA	cis-acting element involved in low-temperature responsiveness

\+ TGACG-motif

Site Name	Organism	Position	Strand	Matrix score.	sequence	function
TGACG-motif	Hordeum vulgare	791	-	5	TGACG	cis-acting regulatory element involved in the MeJA-responsiveness

29 *F. vesca* mrna05953.1-v1.0-hybrid

\+ ABRE

Site Name	Organism	Position	Strand	Matrix score.	sequence	function
ABRE	Arabidopsis thaliana	51	-	5	ACGTG	cis-acting element involved in the abscisic acid responsiveness
ABRE	Arabidopsis thaliana	961	-	5	ACGTG	cis-acting element involved in the abscisic acid responsiveness

\+ CAT-box

Site Name	Organism	Position	Strand	Matrix score.	sequence	function
CAT-box	Arabidopsis thaliana	622	-	6	GCCACT	cis-acting regulatory element related to meristem expression

\+ ERE

Site Name	Organism	Position	Strand	Matrix score.	sequence	function
ERE	Nicotiana glutinos	300	-	8	ATTTTAAA	

\+ GARE-motif

Site Name	Organism	Position	Strand	Matrix score.	sequence	function
GARE-motif	Brassica oleracea	1026	-	7	TCTGTTG	gibberellin-responsive element

\+ MBS

Site Name	Organism	Position	Strand	Matrix score.	sequence	function
MBS	Arabidopsis thaliana	728	-	6	CAACTG	MYB binding site involved in drought-inducibility
MBS	Arabidopsis thaliana	448	-	6	CAACTG	MYB binding site involved in drought-inducibility
MBS	Arabidopsis thaliana	1039	+	6	CAACTG	MYB binding site involved in drought-inducibility

\+ TGA-element

Site Name	Organism	Position	Strand	Matrix score.	sequence	function
TGA-element	Brassica oleracea	1325	-	6	AACGAC	auxin-responsive element

30 *A. lyrata* AL8G25570.t1

+ ABRE

Site Name	Organism	Position	Strand	Matrix score.	sequence	function
ABRE	Arabidopsis thaliana	1495	-	5	ACGTG	cis-acting element involved in the abscisic acid responsiveness
ABRE	Arabidopsis thaliana	1205	+	7	AACCCGG	cis-acting element involved in the abscisic acid responsiveness
ABRE	Arabidopsis thaliana	982	+	7	AACCCGG	cis-acting element involved in the abscisic acid responsiveness
ABRE	Arabidopsis thaliana	157	-	7	AACCCGG	cis-acting element involved in the abscisic acid responsiveness

+ CGTCA-motif

Site Name	Organism	Position	Strand	Matrix score.	sequence	function
CGTCA-motif	Hordeum vulgare	1488	+	5	CGTCA	cis-acting regulatory element involved in the MeJA-responsiveness

+ P-box

Site Name	Organism	Position	Strand	Matrix score.	sequence	function
P-box	Oryza sativa	641	+	7	CCTTTTG	gibberellin-responsive element

+ TCA-element

Site Name	Organism	Position	Strand	Matrix score.	sequence	function
TCA-element	Brassica oleracea	1472	-	9	TCAGAAGAGG	cis-acting element involved in salicylic acid responsiveness

+ TGA-element

Site Name	Organism	Position	Strand	Matrix score.	sequence	function

TGA-element	Brassica oleracea	905	-	6	AACGAC	auxin-responsive element
TGA-element	Brassica oleracea	899	+	6	AACGAC	auxin-responsive element
TGA-element	Brassica oleracea	860	-	6	AACGAC	auxin-responsive element
TGA-element	Brassica oleracea	503	+	6	AACGAC	auxin-responsive element
TGA-element	Brassica oleracea	464	-	6	AACGAC	auxin-responsive element
TGA-element	Brassica oleracea	458	+	6	AACGAC	auxin-responsive element

\+ TGACG-motif

Site Name	Organism	Position	Strand	Matrix score.	sequence	function
TGACG-motif	Hordeum vulgare	1488	-	5	TGACG	cis-acting regulatory element involved in the MeJA-responsiveness

\+ dOCT

Site Name	Organism	Position	Strand	Matrix score.	sequence	function
dOCT	Zea mays	728	-	8	CTCGGATC	

31 *A. halleri* Araha.13031s0002.1.p

None

32 *A. lyrata* AL2G22290.t1

\+ ABRE

Site Name	Organism	Position	Strand	Matrix score.	sequence	function
ABRE	Arabidopsis thaliana	631	+	5	ACGTG	cis-acting element involved in the abscisic acid responsiveness
ABRE	Arabidopsis thaliana	558	+	5	ACGTG	cis-acting element involved in the abscisic acid responsiveness

ABRE	Arabidopsis thaliana	786	-	5	ACGTG	cis-acting element involved in the abscisic acid responsiveness

+ ARE

Site Name	Organism	Position	Strand	Matrix score.	sequence	function
ARE	Zea mays	625	-	6	AAACCA	cis-acting regulatory element essential for the anaerobic induction
ARE	Zea mays	669	+	6	AAACCA	cis-acting regulatory element essential for the anaerobic induction
ARE	Zea mays	155	-	6	AAACCA	cis-acting regulatory element essential for the anaerobic induction
ARE	Zea mays	570	-	6	AAACCA	cis-acting regulatory element essential for the anaerobic induction

+ CGTCA-motif

Site Name	Organism	Position	Strand	Matrix score.	sequence	function
CGTCA-motif	Hordeum vulgare	1420	-	5	CGTCA	cis-acting regulatory element involved in the MeJA-responsiveness
CGTCA-motif	Hordeum vulgare	1458	-	5	CGTCA	cis-acting regulatory element involved in the MeJA-responsiveness
CGTCA-motif	Hordeum vulgare	1389	-	5	CGTCA	cis-acting regulatory element involved in the MeJA-responsiveness
CGTCA-motif	Hordeum vulgare	92	-	5	CGTCA	cis-acting regulatory element involved in the MeJA-responsiveness

+ ERE

Site Name	Organism	Position	Strand	Matrix score.	sequence	function

ERE	Nicotiana glutinos	1109	+	8	ATTTTAAA	

+ TC-rich repeats

Site Name	Organism	Position	Strand	Matrix score.	sequence	function
TC-rich repeats	Nicotiana tabacum	1221	+	9	GTTTTCTTAC	cis-acting element involved in defense and stress responsiveness

+ TCA-element

Site Name	Organism	Position	Strand	Matrix score.	sequence	function
TCA-element	Nicotiana tabacum	1216	+	9	CCATCTTTTT	cis-acting element involved in salicylic acid responsiveness
TCA-element	Nicotiana tabacum	1405	-	9	CCATCTTTTT	cis-acting element involved in salicylic acid responsiveness

+ TGA-element

Site Name	Organism	Position	Strand	Matrix score.	sequence	function
TGA-element	Brassica oleracea	732	-	6	AACGAC	auxin-responsive element

+ TGACG-motif

Site Name	Organism	Position	Strand	Matrix score.	sequence	function
TGACG-motif	Hordeum vulgare	1458	+	5	TGACG	cis-acting regulatory element involved in the MeJA-responsiveness
TGACG-motif	Hordeum vulgare	1420	+	5	TGACG	cis-acting regulatory element involved in the MeJA-responsiveness
TGACG-motif	Hordeum vulgare	1389	+	5	TGACG	cis-acting regulatory element involved in the MeJA-responsiveness
TGACG-motif	Hordeum vulgare	92	+	5	TGACG	cis-acting regulatory element involved in the MeJA-responsiveness

+ WUN-motif

Site Name	Organism	Position	Strand	Matrix score.	sequence	function
WUN-motif	Nicotiana glutinosa	1381	-	9	AAATTTCTT	

33 *A. thaliana* AT5G51300.1

+ ABRE

Site Name	Organism	Position	Strand	Matrix score.	sequence	function
ABRE	Arabidopsis thaliana	803	+	5	ACGTG	cis-acting element involved in the abscisic acid responsiveness

+ ARE

Site Name	Organism	Position	Strand	Matrix score.	sequence	function
ARE	Zea mays	1409	-	6	AAACCA	cis-acting regulatory element essential for the anaerobic induction
ARE	Zea mays	186	+	6	AAACCA	cis-acting regulatory element essential for the anaerobic induction
ARE	Zea mays	247	-	6	AAACCA	cis-acting regulatory element essential for the anaerobic induction
ARE	Zea mays	806	-	6	AAACCA	cis-acting regulatory element essential for the anaerobic induction
ARE	Zea mays	323	+	6	AAACCA	cis-acting regulatory element essential for the anaerobic induction

+ AuxRR-core

Site Name	Organism	Position	Strand	Matrix score.	sequence	function
AuxRR-core	Nicotiana tabacum	752	-	7	GGTCCAT	cis-acting regulatory element involved in auxin responsiveness

+ CCGTCC-box

Site Name	Organism	Position	Strand	Matrix score.	sequence	function
CCGTCC-box	Petroselinum hortense	762	-	6	CCGTCC	

+ CGTCA-motif

Site Name	Organism	Position	Strand	Matrix score.	sequence	function
CGTCA-motif	Hordeum vulgare	415	-	5	CGTCA	cis-acting regulatory element involved in the MeJA-responsiveness
CGTCA-motif	Hordeum vulgare	851	-	5	CGTCA	cis-acting regulatory element involved in the MeJA-responsiveness

+ RY-element

Site Name	Organism	Position	Strand	Matrix score.	sequence	function
RY-element	Helianthus annuus	747	+	8	CATGCATG	cis-acting regulatory element involved in seed-specific regulation

+ TC-rich repeats

Site Name	Organism	Position	Strand	Matrix score.	sequence	function
TC-rich repeats	Nicotiana tabacum	1445	-	9	GTTTTCTTAC	cis-acting element involved in defense and stress responsiveness

+ TGA-element

Site Name	Organism	Position	Strand	Matrix score.	sequence	function
TGA-element	Brassica oleracea	1024	-	6	AACGAC	auxin-responsive element
TGA-element	Brassica oleracea	799	+	6	AACGAC	auxin-responsive element

+ TGACG-motif

Site Name	Organism	Position	Strand	Matrix score.	sequence	function
TGACG-motif	Hordeum vulgare	415	+	5	TGACG	cis-acting regulatory element involved in the MeJA-responsiveness
TGACG-motif	Hordeum vulgare	851	+	5	TGACG	cis-acting regulatory element involved in the MeJA-responsiveness

34 *C. rubella* Carubv10025900m

\+ ABRE

Site Name	Organism	Position	Strand	Matrix score.	sequence	function
ABRE	Arabidopsis thaliana	735	+	5	ACGTG	cis-acting element involved in the abscisic acid responsiveness

\+ ARE

Site Name	Organism	Position	Strand	Matrix score.	sequence	function
ARE	Zea mays	738	-	6	AAACCA	cis-acting regulatory element essential for the anaerobic induction

\+ CCGTCC-box

Site Name	Organism	Position	Strand	Matrix score.	sequence	function
CCGTCC-box	Petroselinum hortense	694	-	6	CCGTCC	

\+ CGTCA-motif

Site Name	Organism	Position	Strand	Matrix score.	sequence	function
CGTCA-motif	Hordeum vulgare	657	+	5	CGTCA	cis-acting regulatory element involved in the MeJA-responsiveness
CGTCA-motif	Hordeum vulgare	347	-	5	CGTCA	cis-acting regulatory element involved in the MeJA-responsiveness
CGTCA-motif	Hordeum vulgare	1483	+	5	CGTCA	cis-acting regulatory element involved in the MeJA-responsiveness

\+ GARE-motif

Site Name	Organism	Position	Strand	Matrix score.	sequence	function
GARE-motif	Brassica oleracea	672	+	7	TCTGTTG	gibberellin-responsive element

\+ LTR

Site Name	Organism	Position	Strand	Matrix score.	sequence	function

LTR	Hordeum vulgare	847	-	6	CCGAAA	cis-acting element involved in low-temperature responsiveness

+ P-box

Site Name	Organism	Position	Strand	Matrix score.	sequence	function
P-box	Oryza sativa	308	+	7	CCTTTTG	gibberellin-responsive element

+ RY-element

Site Name	Organism	Position	Strand	Matrix score.	sequence	function
RY-element	Helianthus annuus	679	+	8	CATGCATG	cis-acting regulatory element involved in seed-specific regulation

+ TGA-element

Site Name	Organism	Position	Strand	Matrix score.	sequence	function
TGA-element	Brassica oleracea	959	-	6	AACGAC	auxin-responsive element
TGA-element	Brassica oleracea	731	+	6	AACGAC	auxin-responsive element
TGA-element	Brassica oleracea	796	-	6	AACGAC	auxin-responsive element

+ TGACG-motif

Site Name	Organism	Position	Strand	Matrix score.	sequence	function
TGACG-motif	Hordeum vulgare	1483	-	5	TGACG	cis-acting regulatory element involved in the MeJA-responsiveness
TGACG-motif	Hordeum vulgare	657	-	5	TGACG	cis-acting regulatory element involved in the MeJA-responsiveness
TGACG-motif	Hordeum vulgare	347	+	5	TGACG	cis-acting regulatory element involved in the MeJA-responsiveness

+ WUN-motif

Site Name	Organism	Position	Strand	Matrix score.	sequence	function
WUN-motif	Nicotiana glutinosa	1229	+	8	AAATTACT	

35 *C. grandiflora* Cagra.3782s0026.1.p

\+ ABRE

Site Name	Organism	Position	Strand	Matrix score.	sequence	function
ABRE	Arabidopsis thaliana	677	+	5	ACGTG	cis-acting element involved in the abscisic acid responsiveness

\+ ARE

Site Name	Organism	Position	Strand	Matrix score.	sequence	function
ARE	Zea mays	680	-	6	AAACCA	cis-acting regulatory element essential for the anaerobic induction

\+ CCGTCC-box

Site Name	Organism	Position	Strand	Matrix score.	sequence	function
CCGTCC-box	Petroselinum hortense	636	-	6	CCGTCC	

\+ CGTCA-motif

Site Name	Organism	Position	Strand	Matrix score.	sequence	function
CGTCA-motif	Hordeum vulgare	292	-	5	CGTCA	cis-acting regulatory element involved in the MeJA-responsiveness
CGTCA-motif	Hordeum vulgare	599	+	5	CGTCA	cis-acting regulatory element involved in the MeJA-responsiveness
CGTCA-motif	Hordeum vulgare	1425	+	5	CGTCA	cis-acting regulatory element involved in the MeJA-responsiveness

\+ LTR

Site Name	Organism	Position	Strand	Matrix score.	sequence	function
LTR	Hordeum vulgare	789	-	6	CCGAAA	cis-acting element involved in low-temperature responsiveness

\+ P-box

Site Name	Organism	Position	Strand	Matrix	sequence	function

score.

P-box	Oryza sativa	253	+	7	CCTTTTG	gibberellin-responsive element

+ RY-element

Site Name	Organism	Position	Strand	Matrix score.	sequence	function
RY-element	Helianthus annuus	621	+	8	CATGCATG	cis-acting regulatory element involved in seed-specific regulation

+ TGA-element

Site Name	Organism	Position	Strand	Matrix score.	sequence	function
TGA-element	Brassica oleracea	673	+	6	AACGAC	auxin-responsive element
TGA-element	Brassica oleracea	738	-	6	AACGAC	auxin-responsive element
TGA-element	Brassica oleracea	901	-	6	AACGAC	auxin-responsive element

+ TGACG-motif

Site Name	Organism	Position	Strand	Matrix score.	sequence	function
TGACG-motif	Hordeum vulgare	1425	-	5	TGACG	cis-acting regulatory element involved in the MeJA-responsiveness
TGACG-motif	Hordeum vulgare	292	+	5	TGACG	cis-acting regulatory element involved in the MeJA-responsiveness
TGACG-motif	Hordeum vulgare	599	-	5	TGACG	cis-acting regulatory element involved in the MeJA-responsiveness

+ WUN-motif

Site Name	Organism	Position	Strand	Matrix score.	sequence	function
WUN-motif	Nicotiana glutinosa	1172	+	8	AAATTACT	

36 *E. salsugineum* Thhalv10013307m

\+ ARE

Site Name	Organism	Position	Strand	Matrix score.	sequence	function
ARE	Zea mays	1325	-	6	AAACCA	cis-acting regulatory element essential for the anaerobic induction

\+ GARE-motif

Site Name	Organism	Position	Strand	Matrix score.	sequence	function
GARE-motif	Brassica oleracea	1025	+	7	TCTGTTG	gibberellin-responsive element

\+ TC-rich repeats

Site Name	Organism	Position	Strand	Matrix score.	sequence	function
TC-rich repeats	Nicotiana tabacum	73	+	9	GTTTTCTTAC	cis-acting element involved in defense and stress responsiveness

\+ TGA-element

Site Name	Organism	Position	Strand	Matrix score.	sequence	function
TGA-element	Brassica oleracea	396	-	6	AACGAC	auxin-responsive element

37 *E. salsugineum* Thhalv10012746m

\+ ARE

Site Name	Organism	Position	Strand	Matrix score.	sequence	function
ARE	Zea mays	713	+	6	AAACCA	cis-acting regulatory element essential for the anaerobic induction
ARE	Zea mays	876	+	6	AAACCA	cis-acting regulatory element essential for the anaerobic induction
ARE	Zea mays	577	-	6	AAACCA	cis-acting regulatory element essential for the anaerobic induction

\+ CAT-box

Site Name	Organism	Position	Strand	Matrix	sequence	function

score.

CAT-box	Arabidopsis thaliana	891	+	6	GCCACT	cis-acting regulatory element related to meristem expression

+ CGTCA-motif

Site Name	Organism	Position	Strand	Matrix score.	sequence	function
CGTCA-motif	Hordeum vulgare	59	+	5	CGTCA	cis-acting regulatory element involved in the MeJA-responsiveness

+ ERE

Site Name	Organism	Position	Strand	Matrix score.	sequence	function
ERE	Nicotiana glutinos	1365	-	8	ATTTCATA	

+ MBS

Site Name	Organism	Position	Strand	Matrix score.	sequence	function
MBS	Arabidopsis thaliana	1399	-	6	CAACTG	MYB binding site involved in drought-inducibility

+ TC-rich repeats

Site Name	Organism	Position	Strand	Matrix score.	sequence	function
TC-rich repeats	Nicotiana tabacum	604	-	9	ATTCTCTAAC	cis-acting element involved in defense and stress responsiveness

+ TGACG-motif

Site Name	Organism	Position	Strand	Matrix score.	sequence	function
TGACG-motif	Hordeum vulgare	59	-	5	TGACG	cis-acting regulatory element involved in the MeJA-responsiveness

+ WUN-motif

Site Name	Organism	Position	Strand	Matrix score.	sequence	function
WUN-motif	Nicotiana glutinosa	866	+	9	CCATTTCAA	

38 *E. salsugineum* Thhalv10022568m

+ ABRE

Site Name	Organism	Position	Strand	Matrix score.	sequence	function
ABRE	Arabidopsis thaliana	256	+	5	ACGTG	cis-acting element involved in the abscisic acid responsiveness
ABRE	Arabidopsis thaliana	785	+	5	ACGTG	cis-acting element involved in the abscisic acid responsiveness
ABRE	Arabidopsis thaliana	784	-	6	CACGTG	cis-acting element involved in the abscisic acid responsiveness
ABRE	Arabidopsis thaliana	693	+	5	ACGTG	cis-acting element involved in the abscisic acid responsiveness

+ ARE

Site Name	Organism	Position	Strand	Matrix score.	sequence	function
ARE	Zea mays	74	+	6	AAACCA	cis-acting regulatory element essential for the anaerobic induction
ARE	Zea mays	1212	-	6	AAACCA	cis-acting regulatory element essential for the anaerobic induction
ARE	Zea mays	355	-	6	AAACCA	cis-acting regulatory element essential for the anaerobic induction

+ AuxRR-core

Site Name	Organism	Position	Strand	Matrix score.	sequence	function
AuxRR-core	Nicotiana tabacum	465	+	7	GGTCCAT	cis-acting regulatory element involved in auxin responsiveness

+ CAT-box

Site Name	Organism	Position	Strand	Matrix score.	sequence	function
CAT-box	Arabidopsis thaliana	591	-	6	GCCACT	cis-acting regulatory element related to meristem expression

+ TCA-element

Site Name	Organism	Position	Strand	Matrix score.	sequence	function
TCA-element	Nicotiana tabacum	1340	-	9	CCATCTTTTT	cis-acting element involved in salicylic acid responsiveness

+ dOCT

Site Name	Organism	Position	Strand	Matrix score.	sequence	function
dOCT	Zea mays	319	+	8	CTCGGATC	

39 *B. oleracea capitata* Bol028094

+ ABRE

Site Name	Organism	Position	Strand	Matrix score.	sequence	function
ABRE	Arabidopsis thaliana	1019	-	7	AACCCGG	cis-acting element involved in the abscisic acid responsiveness
ABRE	Arabidopsis thaliana	944	-	5	ACGTG	cis-acting element involved in the abscisic acid responsiveness
ABRE	Arabidopsis thaliana	221	-	5	ACGTG	cis-acting element involved in the abscisic acid responsiveness
ABRE	Arabidopsis thaliana	102	+	5	ACGTG	cis-acting element involved in the abscisic acid responsiveness

+ CGTCA-motif

Site Name	Organism	Position	Strand	Matrix score.	sequence	function
CGTCA-motif	Hordeum vulgare	1262	+	5	CGTCA	cis-acting regulatory element involved in the MeJA-responsiveness
CGTCA-motif	Hordeum vulgare	1413	-	5	CGTCA	cis-acting regulatory element involved in the MeJA-responsiveness

+ ERE

Site Name	Organism	Position	Strand	Matrix score.	sequence	function
ERE	Nicotiana glutinos	1150	+	8	ATTTTAAA	
ERE	Nicotiana glutinos	698	+	8	ATTTTAAA	

+ GARE-motif

Site Name	Organism	Position	Strand	Matrix score.	sequence	function
GARE-motif	Brassica oleracea	1339	-	7	TCTGTTG	gibberellin-responsive element

+ TGA-element

Site Name	Organism	Position	Strand	Matrix score.	sequence	function
TGA-element	Brassica oleracea	1090	-	6	AACGAC	auxin-responsive element
TGA-element	Brassica oleracea	1084	+	6	AACGAC	auxin-responsive element
TGA-element	Brassica oleracea	164	-	6	AACGAC	auxin-responsive element

+ TGACG-motif

Site Name	Organism	Position	Strand	Matrix score.	sequence	function
TGACG-motif	Hordeum vulgare	1262	-	5	TGACG	cis-acting regulatory element involved in the

						MeJA-responsiveness
TGACG-motif	Hordeum vulgare	1413	+	5	TGACG	cis-acting regulatory element involved in the MeJA-responsiveness

+ WUN-motif

Site Name	Organism	Position	Strand	Matrix score.	sequence	function
WUN-motif	Nicotiana glutinosa	768	+	8	AAATTACT	

40 *B. rapa* Brara.C01481.1.p

+ ARE

Site Name	Organism	Position	Strand	Matrix score.	sequence	function
ARE	Zea mays	459	-	6	AAACCA	cis-acting regulatory element essential for the anaerobic induction

+ AuxRR-core

Site Name	Organism	Position	Strand	Matrix score.	sequence	function
AuxRR-core	Nicotiana tabacum	642	-	7	GGTCCAT	cis-acting regulatory element involved in auxin responsiveness

+ CAT-box

Site Name	Organism	Position	Strand	Matrix score.	sequence	function
CAT-box	Arabidopsis thaliana	346	+	6	GCCACT	cis-acting regulatory element related to meristem expression

+ ERE

Site Name	Organism	Position	Strand	Matrix score.	sequence	function
ERE	Nicotiana glutinos	1034	-	8	ATTTTAAA	
ERE	Nicotiana glutinos	892	+	8	ATTTTAAA	

+ GARE-motif

Site Name	Organism	Position	Strand	Matrix score.	sequence	function
GARE-motif	Brassica oleracea	1406	-	7	TCTGTTG	gibberellin-responsive element

+ LTR

Site Name	Organism	Position	Strand	Matrix score.	sequence	function
LTR	Hordeum vulgare	748	-	6	CCGAAA	cis-acting element involved in low-temperature responsiveness
LTR	Hordeum vulgare	327	-	6	CCGAAA	cis-acting element involved in low-temperature responsiveness
LTR	Hordeum vulgare	313	-	6	CCGAAA	cis-acting element involved in low-temperature responsiveness

+ MBS

Site Name	Organism	Position	Strand	Matrix score.	sequence	function
MBS	Arabidopsis thaliana	843	-	6	CAACTG	MYB binding site involved in drought-inducibility

+ TGA-element

Site Name	Organism	Position	Strand	Matrix score.	sequence	function
TGA-element	Brassica oleracea	1355	-	6	AACGAC	auxin-responsive element

41 *E. salsugineum* Thhalv10024523m

\+ ABRE

Site Name	Organism	Position	Strand	Matrix score.	sequence	function
ABRE	Arabidopsis thaliana	141	+	7	TACGGTC	cis-acting element involved in the abscisic acid responsiveness
ABRE	Arabidopsis thaliana	1333	-	5	ACGTG	cis-acting element involved in the abscisic acid responsiveness

\+ ARE

Site Name	Organism	Position	Strand	Matrix score.	sequence	function
ARE	Zea mays	1040	-	6	AAACCA	cis-acting regulatory element essential for the anaerobic induction
ARE	Zea mays	1200	-	6	AAACCA	cis-acting regulatory element essential for the anaerobic induction

\+ AuxRR-core

Site Name	Organism	Position	Strand	Matrix score.	sequence	function
AuxRR-core	Nicotiana tabacum	1395	-	7	GGTCCAT	cis-acting regulatory element involved in auxin responsiveness
AuxRR-core	Nicotiana tabacum	567	+	7	GGTCCAT	cis-acting regulatory element involved in auxin responsiveness

\+ GARE-motif

Site Name	Organism	Position	Strand	Matrix score.	sequence	function
GARE-motif	Brassica oleracea	561	+	7	TCTGTTG	gibberellin-responsive element

+ TATC-box

Site Name	Organism	Position	Strand	Matrix score.	sequence	function
TATC-box	Oryza sativa	909	-	7	TATCCCA	cis-acting element involved in gibberellin-responsiveness

+ TC-rich repeats

Site Name	Organism	Position	Strand	Matrix score.	sequence	function
TC-rich repeats	Nicotiana tabacum	316	-	9	ATTCTCTAAC	cis-acting element involved in defense and stress responsiveness

+ TGA-element

Site Name	Organism	Position	Strand	Matrix score.	sequence	function
TGA-element	Brassica oleracea	730	+	6	AACGAC	auxin-responsive element
TGA-element	Brassica oleracea	958	+	6	AACGAC	auxin-responsive element

42 *S. lycopersicum* Solyc03g093350.2.1

\+ ABRE

Site Name	Organism	Position	Strand	Matrix score.	sequence	function
ABRE	Arabidopsis thaliana	951	-	5	ACGTG	cis-acting element involved in the abscisic acid responsiveness

\+ ARE

Site Name	Organism	Position	Strand	Matrix score.	sequence	function
ARE	Zea mays	1426	+	6	AAACCA	cis-acting regulatory element essential for the anaerobic induction
ARE	Zea mays	1260	+	6	AAACCA	cis-acting regulatory element essential for the anaerobic induction

\+ CGTCA-motif

Site Name	Organism	Position	Strand	Matrix score.	sequence	function
CGTCA-motif	Hordeum vulgare	189	-	5	CGTCA	cis-acting regulatory element involved in the MeJA-responsiveness
CGTCA-motif	Hordeum vulgare	1486	-	5	CGTCA	cis-acting regulatory element involved in the MeJA-responsiveness
CGTCA-motif	Hordeum vulgare	1155	+	5	CGTCA	cis-acting regulatory element involved in the MeJA-responsiveness

\+ ERE

Site Name	Organism	Position	Strand	Matrix score.	sequence	function
ERE	Nicotiana glutinos	1160	+	8	ATTTTAAA	
ERE	Nicotiana glutinos	399	-	8	ATTTTAAA	

\+ TC-rich repeats

Site Name	Organism	Position	Strand	Matrix score.	sequence	function
TC-rich repeats	Nicotiana tabacum	993	-	10	GTTTTCTTAC	cis-acting element

involved in defense and stress responsiveness

+ TCA-element

Site Name	Organism	Position	Strand	Matrix score.	sequence	function
TCA-element	Nicotiana tabacum	1239	+	9	CCATCTTTTT	cis-acting element involved in salicylic acid responsiveness
TCA-element	Nicotiana tabacum	1384	+	9	CCATCTTTTT	cis-acting element involved in salicylic acid responsiveness

+ TGACG-motif

Site Name	Organism	Position	Strand	Matrix score.	sequence	function
TGACG-motif	Hordeum vulgare	1486	+	5	TGACG	cis-acting regulatory element involved in the MeJA-responsiveness
TGACG-motif	Hordeum vulgare	189	+	5	TGACG	cis-acting regulatory element involved in the MeJA-responsiveness
TGACG-motif	Hordeum vulgare	1155	-	5	TGACG	cis-acting regulatory element involved in the MeJA-responsiveness

43 *S. tuberosum* PGSC0003DMP400032853

+ ABRE

Site Name	Organism	Position	Strand	Matrix score.	sequence	function
ABRE	Arabidopsis thaliana	431	-	5	ACGTG	cis-acting element involved in the abscisic acid responsiveness
ABRE	Arabidopsis thaliana	50	+	5	ACGTG	cis-acting element involved in the abscisic acid responsiveness

+ ARE

Site Name	Organism	Position	Strand	Matrix score.	sequence	function
ARE	Zea mays	242	+	6	AAACCA	cis-acting regulatory element essential for the anaerobic induction
ARE	Zea mays	1418	-	6	AAACCA	cis-acting regulatory element essential for the anaerobic induction

+ CAT-box

Site Name	Organism	Position	Strand	Matrix score.	sequence	function
CAT-box	Arabidopsis thaliana	693	+	6	GCCACT	cis-acting regulatory element related to meristem expression
CAT-box	Arabidopsis thaliana	302	+	6	GCCACT	cis-acting regulatory element related to meristem expression
CAT-box	Arabidopsis thaliana	642	-	6	GCCACT	cis-acting regulatory element related to meristem expression

+ CGTCA-motif

Site Name	Organism	Position	Strand	Matrix score.	sequence	function
CGTCA-motif	Hordeum vulgare	589	+	5	CGTCA	cis-acting regulatory element involved in the MeJA-responsiveness

+ TCA-element

Site Name	Organism	Position	Strand	Matrix score.	sequence	function
TCA-element	Nicotiana tabacum	886	+	9	CCATCTTTTT	cis-acting element involved in salicylic acid responsiveness

+ TGACG-motif

Site Name	Organism	Position	Strand	Matrix score.	sequence	function
TGACG-motif	Hordeum vulgare	589	-	5	TGACG	cis-acting regulatory element involved in the MeJA-responsiveness

44 *S. tuberosum* PGSC0003DMP400012835

+ ARE

Site Name	Organism	Position	Strand	Matrix score.	sequence	function
ARE	Zea mays	1357	-	6	AAACCA	cis-acting regulatory element essential for the anaerobic induction

+ CAT-box

Site Name	Organism	Position	Strand	Matrix score.	sequence	function
CAT-box	Arabidopsis thaliana	1346	-	6	GCCACT	cis-acting regulatory element related to meristem expression
CAT-box	Arabidopsis thaliana	1076	-	6	GCCACT	cis-acting regulatory element related to meristem expression

+ LTR

Site Name	Organism	Position	Strand	Matrix score.	sequence	function
LTR	Hordeum vulgare	865	+	6	CCGAAA	cis-acting element involved in low-temperature responsiveness

45 *M. guttatus* Migut.F01191.1.p

\+ ABRE

Site Name	Organism	Position	Strand	Matrix score.	sequence	function
ABRE	Arabidopsis thaliana	829	-	5	ACGTG	cis-acting element involved in the abscisic acid responsiveness
ABRE	Hordeum vulgare	130	+	9	GCAACGTGTC	cis-acting element involved in the abscisic acid responsiveness
ABRE	Arabidopsis thaliana	243	-	5	ACGTG	cis-acting element involved in the abscisic acid responsiveness

\+ ARE

Site Name	Organism	Position	Strand	Matrix score.	sequence	function
ARE	Zea mays	623	+	6	AAACCA	cis-acting regulatory element essential for the anaerobic induction

\+ CCGTCC-box

Site Name	Organism	Position	Strand	Matrix score.	sequence	function
CCGTCC-box	Petroselinum hortense	412	+	6	CCGTCC	

\+ CGTCA-motif

Site Name	Organism	Position	Strand	Matrix score.	sequence	function
CGTCA-motif	Hordeum vulgare	320	-	5	CGTCA	cis-acting regulatory element involved in the MeJA-responsiveness
CGTCA-motif	Hordeum vulgare	952	+	5	CGTCA	cis-acting regulatory element involved in the MeJA-responsiveness
CGTCA-motif	Hordeum vulgare	406	-	5	CGTCA	cis-acting regulatory element involved in the MeJA-responsiveness

+ GC-motif

Site Name	Organism	Position	Strand	Matrix score.	sequence	function
GC-motif	Zea mays	299	+	6	CCCCCG	enhancer-like element involved in anoxic specific inducibility

+ LTR

Site Name	Organism	Position	Strand	Matrix score.	sequence	function
LTR	Hordeum vulgare	1093	+	6	CCGAAA	cis-acting element involved in low-temperature responsiveness
LTR	Hordeum vulgare	849	+	6	CCGAAA	cis-acting element involved in low-temperature responsiveness
LTR	Hordeum vulgare	279	-	6	CCGAAA	cis-acting element involved in low-temperature responsiveness

+ MBS

Site Name	Organism	Position	Strand	Matrix score.	sequence	function
MBS	Arabidopsis thaliana	1219	+	6	CAACTG	MYB binding site involved in drought-inducibility
MBS	Arabidopsis thaliana	1299	-	6	CAACTG	MYB binding site involved in drought-inducibility

+ P-box

Site Name	Organism	Position	Strand	Matrix score.	sequence	function
P-box	Oryza sativa	58	-	7	CCTTTTG	gibberellin-responsive element

+ RY-element

Site Name	Organism	Position	Strand	Matrix score.	sequence	function
RY-element	Helianthus annuus	1256	-	8	CATGCATG	cis-acting regulatory element involved in seed-specific regulation

+ TCA-element

Site Name	Organism	Position	Strand	Matrix score.	sequence	function
TCA-element	Brassica oleracea	82	+	9	TCAGAAGAGG	cis-acting element involved in salicylic acid responsiveness

+ TGA-element

Site Name	Organism	Position	Strand	Matrix score.	sequence	function
TGA-element	Brassica oleracea	457	+	6	AACGAC	auxin-responsive element

+ TGACG-motif

Site Name	Organism	Position	Strand	Matrix score.	sequence	function
TGACG-motif	Hordeum vulgare	952	-	5	TGACG	cis-acting regulatory element involved in the MeJA-responsiveness
TGACG-motif	Hordeum vulgare	320	+	5	TGACG	cis-acting regulatory element involved in the MeJA-responsiveness
TGACG-motif	Hordeum vulgare	406	+	5	TGACG	cis-acting regulatory element involved in the MeJA-responsiveness

46 *K. laxiflora* Kalax.0858s0015.1.p

+ ABRE

Site Name	Organism	Position	Strand	Matrix score.	sequence	function
ABRE	Arabidopsis thaliana	85	-	5	ACGTG	cis-acting element involved in the abscisic acid responsiveness
ABRE	Arabidopsis thaliana	268	-	5	ACGTG	cis-acting element involved in the abscisic acid responsiveness

+ ARE

Site Name	Organism	Position	Strand	Matrix score.	sequence	function
ARE	Zea mays	143	+	6	AAACCA	cis-acting regulatory element essential for the anaerobic induction
ARE	Zea mays	1151	-	6	AAACCA	cis-acting regulatory element essential for the anaerobic induction
ARE	Zea mays	1455	+	6	AAACCA	cis-acting regulatory element essential for the anaerobic induction

+ CGTCA-motif

Site Name	Organism	Position	Strand	Matrix score.	sequence	function
CGTCA-motif	Hordeum vulgare	1261	+	5	CGTCA	cis-acting regulatory element involved in the MeJA-responsiveness
CGTCA-motif	Hordeum vulgare	739	+	5	CGTCA	cis-acting regulatory element involved in the MeJA-responsiveness

+ GARE-motif

Site Name	Organism	Position	Strand	Matrix score.	sequence	function
GARE-motif	Brassica oleracea	633	+	7	TCTGTTG	gibberellin-responsive element
GARE-motif	Brassica oleracea	907	-	7	TCTGTTG	gibberellin-responsive element
GARE-motif	Brassica oleracea	919	-	7	TCTGTTG	gibberellin-responsive element

+ GC-motif

Site Name	Organism	Position	Strand	Matrix	sequence	function

				score.		
GC-motif	Zea mays	285	+	6	CCCCCG	enhancer-like element involved in anoxic specific inducibility

+ MBS

Site Name	Organism	Position	Strand	Matrix score.	sequence	function
MBS	Arabidopsis thaliana	480	+	6	CAACTG	MYB binding site involved in drought-inducibility
MBS	Arabidopsis thaliana	1289	+	6	CAACTG	MYB binding site involved in drought-inducibility

+ TC-rich repeats

Site Name	Organism	Position	Strand	Matrix score.	sequence	function
TC-rich repeats	Nicotiana tabacum	372	-	9	GTTTTCTTAC	cis-acting element involved in defense and stress responsiveness

+ TCA-element

Site Name	Organism	Position	Strand	Matrix score.	sequence	function
TCA-element	Nicotiana tabacum	649	+	9	CCATCTTTTT	cis-acting element involved in salicylic acid responsiveness
TCA-element	Nicotiana tabacum	244	-	9	CCATCTTTTT	cis-acting element involved in salicylic acid responsiveness

+ TGACG-motif

Site Name	Organism	Position	Strand	Matrix score.	sequence	function
TGACG-motif	Hordeum vulgare	1261	-	5	TGACG	cis-acting regulatory element involved in the MeJA-responsiveness
TGACG-motif	Hordeum vulgare	739	-	5	TGACG	cis-acting regulatory element involved in the MeJA-responsiveness

+ WUN-motif

Site Name	Organism	Position	Strand	Matrix score.	sequence	function
WUN-motif	Nicotiana glutinosa	1396	+	9	AAATTACTA	

47 ***K. fedtschenkoi*** **Kaladp0055s0379.1.p**

+ ARE

Site Name	Organism	Position	Strand	Matrix score.	sequence	function
ARE	Zea mays	1174	+	6	AAACCA	cis-acting regulatory element essential for the anaerobic induction
ARE	Zea mays	1451	+	6	AAACCA	cis-acting regulatory element essential for the anaerobic induction
ARE	Zea mays	400	-	6	AAACCA	cis-acting regulatory element essential for the anaerobic induction
ARE	Zea mays	1101	+	6	AAACCA	cis-acting regulatory element essential for the anaerobic induction

+ CGTCA-motif

Site Name	Organism	Position	Strand	Matrix score.	sequence	function
CGTCA-motif	Hordeum vulgare	893	+	5	CGTCA	cis-acting regulatory element involved in the MeJA-responsiveness
CGTCA-motif	Hordeum vulgare	239	+	5	CGTCA	cis-acting regulatory element involved in the MeJA-responsiveness
CGTCA-motif	Hordeum vulgare	515	+	5	CGTCA	cis-acting regulatory element involved in the MeJA-responsiveness

+ ERE

Site Name	Organism	Position	Strand	Matrix score.	sequence	function
ERE	Nicotiana glutinos	678	-	8	ATTTTAAA	
ERE	Nicotiana glutinos	813	-	8	ATTTTAAA	
ERE	Nicotiana glutinos	781	-	8	ATTTTAAA	

+ GARE-motif

Site Name	Organism	Position	Strand	Matrix score.	sequence	function
GARE-motif	Brassica oleracea	162	-	7	TCTGTTG	gibberellin-responsive element
GARE-motif	Brassica oleracea	150	-	7	TCTGTTG	gibberellin-responsive element

+ MBS

Site Name	Organism	Position	Strand	Matrix score.	sequence	function
MBS	Arabidopsis thaliana	544	+	6	CAACTG	MYB binding site involved in drought-inducibility

+ TGA-element

Site Name	Organism	Position	Strand	Matrix score.	sequence	function
TGA-element	Brassica oleracea	617	-	6	AACGAC	auxin-responsive element

+ TGACG-motif

Site Name	Organism	Position	Strand	Matrix score.	sequence	function
TGACG-motif	Hordeum vulgare	239	-	5	TGACG	cis-acting regulatory element involved in the MeJA-responsiveness
TGACG-motif	Hordeum vulgare	893	-	5	TGACG	cis-acting regulatory element involved in the MeJA-responsiveness
TGACG-motif	Hordeum vulgare	515	-	5	TGACG	cis-acting regulatory element involved in the MeJA-responsiveness

48 *K. laxiflora* Kalax.1180s0005.1.p

+ ABRE

Site Name	Organism	Position	Strand	Matrix score.	sequence	function
ABRE	Arabidopsis thaliana	327	-	5	ACGTG	cis-acting element involved in the abscisic acid responsiveness
ABRE	Arabidopsis thaliana	510	-	5	ACGTG	cis-acting element involved in the abscisic acid responsiveness

+ ARE

Site Name	Organism	Position	Strand	Matrix score.	sequence	function
ARE	Zea mays	385	+	6	AAACCA	cis-acting regulatory element essential for the anaerobic induction
ARE	Zea mays	1452	+	6	AAACCA	cis-acting regulatory element essential for the anaerobic induction
ARE	Zea mays	1151	-	6	AAACCA	cis-acting regulatory element essential for the anaerobic induction

+ CGTCA-motif

Site Name	Organism	Position	Strand	Matrix score.	sequence	function
CGTCA-motif	Hordeum vulgare	73	-	5	CGTCA	cis-acting regulatory element involved in the MeJA-responsiveness
CGTCA-motif	Hordeum vulgare	1260	+	5	CGTCA	cis-acting regulatory element involved in the MeJA-responsiveness
CGTCA-motif	Hordeum vulgare	1000	+	5	CGTCA	cis-acting regulatory element involved in the MeJA-responsiveness

+ GARE-motif

Site Name	Organism	Position	Strand	Matrix	sequence	function

score.

GARE-motif	Brassica oleracea	922	-	7	TCTGTTG	gibberellin-responsive element
GARE-motif	Brassica oleracea	910	-	7	TCTGTTG	gibberellin-responsive element

+ GC-motif

Site Name	Organism	Position	Strand	Matrix score.	sequence	function
GC-motif	Zea mays	527	+	6	CCCCCG	enhancer-like element involved in anoxic specific inducibility

+ MBS

Site Name	Organism	Position	Strand	Matrix score.	sequence	function
MBS	Arabidopsis thaliana	57	+	6	CAACTG	MYB binding site involved in drought-inducibility
MBS	Arabidopsis thaliana	721	+	6	CAACTG	MYB binding site involved in drought-inducibility
MBS	Arabidopsis thaliana	859	+	6	CAACTG	MYB binding site involved in drought-inducibility
MBS	Arabidopsis thaliana	1288	+	6	CAACTG	MYB binding site involved in drought-inducibility

+ TC-rich repeats

Site Name	Organism	Position	Strand	Matrix score.	sequence	function
TC-rich repeats	Nicotiana tabacum	614	-	9	GTTTTCTTAC	cis-acting element involved in defense and stress responsiveness

+ TCA-element

Site Name	Organism	Position	Strand	Matrix score.	sequence	function
TCA-element	Nicotiana tabacum	486	-	9	CCATCTTTTT	cis-acting element involved in salicylic acid responsiveness

+ TGA-element

Site Name	Organism	Position	Strand	Matrix score.	sequence	function
TGA-element	Brassica oleracea	1121	-	6	AACGAC	auxin-responsive element

+ TGACG-motif

Site Name	Organism	Position	Strand	Matrix score.	sequence	function
TGACG-motif	Hordeum vulgare	73	+	5	TGACG	cis-acting regulatory element involved in the MeJA-responsiveness
TGACG-motif	Hordeum vulgare	1260	-	5	TGACG	cis-acting regulatory element involved in the MeJA-responsiveness
TGACG-motif	Hordeum vulgare	1000	-	5	TGACG	cis-acting regulatory element involved in the MeJA-responsiveness

+ WUN-motif

Site Name	Organism	Position	Strand	Matrix score.	sequence	function
WUN-motif	Nicotiana glutinosa	1393	+	9	AAATTACTA	

49 *K. fedtschenkoi* Kaladp0095s0260.1.p

+ ABRE

Site Name	Organism	Position	Strand	Matrix score.	sequence	function
ABRE	Arabidopsis thaliana	1264	+	5	ACGTG	cis-acting element involved in the abscisic acid responsiveness
ABRE	Arabidopsis thaliana	1047	-	7	AACCCGG	cis-acting element involved in the abscisic acid responsiveness
ABRE	Arabidopsis thaliana	867	+	5	ACGTG	cis-acting element involved in the abscisic acid responsiveness
ABRE	Arabidopsis thaliana	693	+	5	ACGTG	cis-acting element involved in the abscisic acid responsiveness
ABRE	Arabidopsis thaliana	1263	-	6	CACGTG	cis-acting element involved in the abscisic acid responsiveness
ABRE	Arabidopsis thaliana	702	+	5	ACGTG	cis-acting element involved in the abscisic acid responsiveness

+ ARE

Site Name	Organism	Position	Strand	Matrix score.	sequence	function
ARE	Zea mays	1134	+	6	AAACCA	cis-acting regulatory element essential for the anaerobic induction

+ CGTCA-motif

Site Name	Organism	Position	Strand	Matrix score.	sequence	function
CGTCA-motif	Hordeum vulgare	1480	-	5	CGTCA	cis-acting regulatory element involved in the MeJA-responsiveness
CGTCA-motif	Hordeum vulgare	118	+	5	CGTCA	cis-acting regulatory element involved in the MeJA-responsiveness
CGTCA-motif	Hordeum vulgare	1239	-	5	CGTCA	cis-acting regulatory element involved in the MeJA-responsiveness
CGTCA-motif	Hordeum vulgare	1267	-	5	CGTCA	cis-acting regulatory element involved in the MeJA-responsiveness
CGTCA-motif	Hordeum vulgare	193	+	5	CGTCA	cis-acting regulatory element involved in the MeJA-responsiveness

+ LTR

Site Name	Organism	Position	Strand	Matrix score.	sequence	function
LTR	Hordeum vulgare	996	+	6	CCGAAA	cis-acting element involved in low-temperature responsiveness
LTR	Hordeum vulgare	371	-	6	CCGAAA	cis-acting element involved in low-temperature responsiveness

+ MBS

Site Name	Organism	Position	Strand	Matrix score.	sequence	function
MBS	Arabidopsis thaliana	361	+	6	CAACTG	MYB binding site involved in drought-inducibility
MBS	Arabidopsis thaliana	27	-	6	CAACTG	MYB binding site involved in drought-inducibility
MBS	Arabidopsis thaliana	121	+	6	CAACTG	MYB binding site involved in drought-inducibility
MBS	Arabidopsis thaliana	413	-	6	CAACTG	MYB binding site involved in drought-inducibility

+ TGACG-motif

Site Name	Organism	Position	Strand	Matrix score.	sequence	function
TGACG-motif	Hordeum vulgare	1480	+	5	TGACG	cis-acting regulatory element involved in the MeJA-responsiveness
TGACG-motif	Hordeum vulgare	193	-	5	TGACG	cis-acting regulatory element involved in the MeJA-responsiveness
TGACG-motif	Hordeum vulgare	118	-	5	TGACG	cis-acting regulatory element involved in the MeJA-responsiveness
TGACG-motif	Hordeum vulgare	1267	+	5	TGACG	cis-acting regulatory element involved in the MeJA-responsiveness
TGACG-motif	Hordeum vulgare	1239	+	5	TGACG	cis-acting regulatory element involved in the MeJA-responsiveness

50 *K. laxiflora* Kalax.0066s0089.1.p

+ ABRE

Site Name	Organism	Position	Strand	Matrix score.	sequence	function
ABRE	Arabidopsis thaliana	1234	+	5	ACGTG	cis-acting element involved in the abscisic acid responsiveness
ABRE	Arabidopsis thaliana	1017	-	7	AACCCGG	cis-acting element involved in the abscisic acid responsiveness
ABRE	Arabidopsis thaliana	1171	-	6	CACGTG	cis-acting element involved in the abscisic acid responsiveness
ABRE	Arabidopsis thaliana	1172	+	5	ACGTG	cis-acting element involved in the abscisic acid responsiveness
ABRE	Arabidopsis thaliana	837	+	5	ACGTG	cis-acting element involved in the abscisic acid responsiveness
ABRE	Arabidopsis thaliana	663	+	5	ACGTG	cis-acting element involved in the abscisic acid responsiveness
ABRE	Arabidopsis thaliana	672	+	5	ACGTG	cis-acting element involved in the abscisic acid responsiveness

+ ARE

Site Name	Organism	Position	Strand	Matrix score.	sequence	function
ARE	Zea mays	1104	+	6	AAACCA	cis-acting regulatory element essential for the anaerobic induction

+ CGTCA-motif

Site Name	Organism	Position	Strand	Matrix score.	sequence	function
CGTCA-motif	Hordeum vulgare	88	+	5	CGTCA	cis-acting regulatory element involved in the MeJA-responsiveness
CGTCA-motif	Hordeum vulgare	1459	-	5	CGTCA	cis-acting regulatory element involved in the MeJA-responsiveness
CGTCA-motif	Hordeum vulgare	1175	-	5	CGTCA	cis-acting regulatory element involved in the MeJA-responsiveness
CGTCA-motif	Hordeum vulgare	1209	-	5	CGTCA	cis-acting regulatory element involved in the MeJA-responsiveness

CGTCA-motif	Hordeum vulgare	163	+	5	CGTCA	cis-acting regulatory element involved in the MeJA-responsiveness

+ LTR

Site Name	Organism	Position	Strand	Matrix score.	sequence	function
LTR	Hordeum vulgare	341	-	6	CCGAAA	cis-acting element involved in low-temperature responsiveness

+ MBS

Site Name	Organism	Position	Strand	Matrix score.	sequence	function
MBS	Arabidopsis thaliana	91	+	6	CAACTG	MYB binding site involved in drought-inducibility
MBS	Arabidopsis thaliana	331	+	6	CAACTG	MYB binding site involved in drought-inducibility
MBS	Arabidopsis thaliana	383	-	6	CAACTG	MYB binding site involved in drought-inducibility

+ TGACG-motif

Site Name	Organism	Position	Strand	Matrix score.	sequence	function
TGACG-motif	Hordeum vulgare	1459	+	5	TGACG	cis-acting regulatory element involved in the MeJA-responsiveness
TGACG-motif	Hordeum vulgare	163	-	5	TGACG	cis-acting regulatory element involved in the MeJA-responsiveness
TGACG-motif	Hordeum vulgare	88	-	5	TGACG	cis-acting regulatory element involved in the MeJA-responsiveness
TGACG-motif	Hordeum vulgare	1175	+	5	TGACG	cis-acting regulatory element involved in the MeJA-responsiveness
TGACG-motif	Hordeum vulgare	1209	+	5	TGACG	cis-acting regulatory element involved in the MeJA-responsiveness

51 *C. quinoa* AUR62016572-RA

+ ABRE

Site Name	Organism	Position	Strand	Matrix score.	sequence	function
ABRE	Arabidopsis thaliana	803	+	5	ACGTG	cis-acting element involved in the abscisic acid responsiveness
ABRE	Arabidopsis thaliana	493	-	5	ACGTG	cis-acting element involved in the abscisic acid responsiveness
ABRE	Arabidopsis thaliana	802	-	6	CACGTG	cis-acting element involved in the abscisic acid responsiveness

+ ARE

Site Name	Organism	Position	Strand	Matrix score.	sequence	function
ARE	Zea mays	1402	+	6	AAACCA	cis-acting regulatory element essential for the anaerobic induction

+ CGTCA-motif

Site Name	Organism	Position	Strand	Matrix score.	sequence	function
CGTCA-motif	Hordeum vulgare	2	-	5	CGTCA	cis-acting regulatory element involved in the MeJA-responsiveness

+ LTR

Site Name	Organism	Position	Strand	Matrix score.	sequence	function
LTR	Hordeum vulgare	921	-	6	CCGAAA	cis-acting element involved in low-temperature responsiveness
LTR	Hordeum vulgare	1301	+	6	CCGAAA	cis-acting element involved in low-temperature responsiveness

+ MBS

Site Name	Organism	Position	Strand	Matrix score.	sequence	function
MBS	Arabidopsis thaliana	1336	+	6	CAACTG	MYB binding site involved in drought-inducibility

+ P-box

Site Name	Organism	Position	Strand	Matrix score.	sequence	function
P-box	Oryza sativa	305	+	7	CCTTTTG	gibberellin-responsive element

+ TC-rich repeats

Site Name	Organism	Position	Strand	Matrix score.	sequence	function
TC-rich repeats	Nicotiana tabacum	910	+	9	ATTCTCTAAC	cis-acting element involved in defense and stress responsiveness

+ TCA-element

Site Name	Organism	Position	Strand	Matrix score.	sequence	function
TCA-element	Nicotiana tabacum	1439	+	9	CCATCTTTTT	cis-acting element involved in salicylic acid responsiveness

+ TGACG-motif

Site Name	Organism	Position	Strand	Matrix score.	sequence	function
TGACG-motif	Hordeum vulgare	2	+	5	TGACG	cis-acting regulatory element involved in the MeJA-responsiveness

52 *C. quinoa* AUR62003728-RA

+ ARE

Site Name	Organism	Position	Strand	Matrix score.	sequence	function
ARE	Zea mays	1393	+	6	AAACCA	cis-acting regulatory element essential for the anaerobic induction
ARE	Zea mays	477	-	6	AAACCA	cis-acting regulatory element essential for the anaerobic induction
ARE	Zea mays	878	+	6	AAACCA	cis-acting regulatory element essential for the anaerobic induction

+ CGTCA-motif

Site Name	Organism	Position	Strand	Matrix score.	sequence	function
CGTCA-motif	Hordeum vulgare	10	-	5	CGTCA	cis-acting regulatory element involved in the MeJA-responsiveness

+ GC-motif

Site Name	Organism	Position	Strand	Matrix score.	sequence	function
GC-motif	Zea mays	943	+	6	CCCCCG	enhancer-like element involved in anoxic specific inducibility

+ LTR

Site Name	Organism	Position	Strand	Matrix score.	sequence	function
LTR	Hordeum vulgare	1169	+	6	CCGAAA	cis-acting element involved in low-temperature responsiveness

+ P-box

Site Name	Organism	Position	Strand	Matrix score.	sequence	function
P-box	Oryza sativa	315	+	7	CCTTTTG	gibberellin-responsive element

+ TATC-box

Site Name	Organism	Position	Strand	Matrix score.	sequence	function
TATC-box	Oryza sativa	1289	+	7	TATCCCA	cis-acting element involved in gibberellin-responsiveness

+ TCA-element

Site Name	Organism	Position	Strand	Matrix score.	sequence	function
TCA-element	Nicotiana tabacum	1438	+	9	CCATCTTTTT	cis-acting element involved in salicylic acid responsiveness

+ TGACG-motif

Site Name	Organism	Position	Strand	Matrix score.	sequence	function
TGACG-motif	Hordeum vulgare	10	+	5	TGACG	cis-acting regulatory element involved in the MeJA-responsiveness

53 *A. hypochondriacus* AHYPO_014098-RA

+ ABRE

Site Name	Organism	Position	Strand	Matrix score.	sequence	function
ABRE	Arabidopsis thaliana	428	+	5	ACGTG	cis-acting element involved in the abscisic acid responsiveness

+ ERE

Site Name	Organism	Position	Strand	Matrix score.	sequence	function
ERE	Nicotiana glutinos	1056	+	8	ATTTTAAA	

+ GC-motif

Site Name	Organism	Position	Strand	Matrix score.	sequence	function
GC-motif	Zea mays	685	-	6	CCCCCG	enhancer-like element involved in anoxic specific inducibility

+ MBS

Site Name	Organism	Position	Strand	Matrix score.	sequence	function
MBS	Arabidopsis thaliana	499	-	6	CAACTG	MYB binding site involved in drought-inducibility
MBS	Arabidopsis thaliana	646	+	6	CAACTG	MYB binding site involved in drought-inducibility

+ TC-rich repeats

Site Name	Organism	Position	Strand	Matrix score.	sequence	function
TC-rich repeats	Nicotiana tabacum	399	-	9	GTTTTCTTAC	cis-acting element involved in defense and stress responsiveness

+ TGA-element

Site Name	Organism	Position	Strand	Matrix score.	sequence	function
TGA-element	Brassica oleracea	1087	-	6	AACGAC	auxin-responsive element

+ WUN-motif

Site Name	Organism	Position	Strand	Matrix score.	sequence	function
WUN-motif	Nicotiana glutinosa	1152	-	9	AAATTACTA	
WUN-motif	Nicotiana glutinosa	1153	-	8	AAATTACT	

54 *D. carota* DCAR_006843

+ ARE

Site Name	Organism	Position	Strand	Matrix score.	sequence	function
ARE	Zea mays	351	-	6	AAACCA	cis-acting regulatory element essential for the anaerobic induction

+ CAT-box

Site Name	Organism	Position	Strand	Matrix score.	sequence	function
CAT-box	Arabidopsis thaliana	274	+	6	GCCACT	cis-acting regulatory element related to meristem expression
CAT-box	Arabidopsis thaliana	1288	-	6	GCCACT	cis-acting regulatory element related to meristem expression

+ ERE

Site Name	Organism	Position	Strand	Matrix score.	sequence	function
ERE	Nicotiana glutinos	980	+	8	ATTTTAAA	

+ TGA-element

Site Name	Organism	Position	Strand	Matrix score.	sequence	function
TGA-element	Brassica oleracea	1027	+	6	AACGAC	auxin-responsive element

+ WUN-motif

Site Name	Organism	Position	Strand	Matrix score.	sequence	function
WUN-motif	Nicotiana glutinosa	123	-	9	AAATTTCTT	

55 *D. carota* DCAR_008506

+ ABRE

Site Name	Organism	Position	Strand	Matrix score.	sequence	function
ABRE	Arabidopsis thaliana	1019	+	7	AACCCGG	cis-acting element involved in the abscisic acid responsiveness

+ ARE

Site Name	Organism	Position	Strand	Matrix score.	sequence	function
ARE	Zea mays	225	+	6	AAACCA	cis-acting regulatory element essential for the anaerobic induction
ARE	Zea mays	88	-	6	AAACCA	cis-acting regulatory element essential for the anaerobic induction
ARE	Zea mays	218	+	6	AAACCA	cis-acting regulatory element essential for the anaerobic induction

+ CGTCA-motif

Site Name	Organism	Position	Strand	Matrix score.	sequence	function
CGTCA-motif	Hordeum vulgare	1477	+	5	CGTCA	cis-acting regulatory element involved in the MeJA-responsiveness

+ ERE

Site Name	Organism	Position	Strand	Matrix score.	sequence	function
ERE	Nicotiana glutinos	1121	-	8	ATTTTAAA	
ERE	Nicotiana glutinos	1119	+	8	ATTTTAAA	
ERE	Nicotiana glutinos	274	-	8	ATTTTAAA	
ERE	Nicotiana glutinos	1266	+	8	ATTTTAAA	

+ LTR

Site Name	Organism	Position	Strand	Matrix score.	sequence	function
LTR	Hordeum vulgare	1291	+	6	CCGAAA	cis-acting element involved in low-temperature responsiveness

+ MBS

Site Name	Organism	Position	Strand	Matrix score.	sequence	function
MBS	Arabidopsis thaliana	84	-	6	CAACTG	MYB binding site involved in drought-inducibility

+ TC-rich repeats

Site Name	Organism	Position	Strand	Matrix score.	sequence	function
TC-rich repeats	Nicotiana tabacum	855	+	9	GTTTTCTTAC	cis-acting element involved in defense and stress responsiveness

+ TGACG-motif

Site Name	Organism	Position	Strand	Matrix score.	sequence	function
TGACG-motif	Hordeum vulgare	1477	-	5	TGACG	cis-acting regulatory element involved in the MeJA-responsiveness

+ WUN-motif

Site Name	Organism	Position	Strand	Matrix score.	sequence	function
WUN-motif	Nicotiana glutinosa	1272	-	9	AAATTTCTT	

56 *D. carota* DCAR_004968

+ ABRE

Site Name	Organism	Position	Strand	Matrix score.	sequence	function
ABRE	Arabidopsis thaliana	1056	-	7	TACGGTC	cis-acting element involved in the abscisic acid responsiveness

+ ARE

Site Name	Organism	Position	Strand	Matrix score.	sequence	function
ARE	Zea mays	801	+	6	AAACCA	cis-acting regulatory element essential for the anaerobic induction

+ CAT-box

Site Name	Organism	Position	Strand	Matrix score.	sequence	function
CAT-box	Arabidopsis thaliana	178	-	6	GCCACT	cis-acting regulatory element related to meristem expression

+ CGTCA-motif

Site Name	Organism	Position	Strand	Matrix score.	sequence	function
CGTCA-motif	Hordeum vulgare	1374	-	5	CGTCA	cis-acting regulatory element involved in the MeJA-responsiveness

+ ERE

Site Name	Organism	Position	Strand	Matrix score.	sequence	function
ERE	Nicotiana glutinos	611	-	8	ATTTTAAA	
ERE	Nicotiana glutinos	259	+	8	ATTTTAAA	

+ GCN4_motif

Site Name	Organism	Position	Strand	Matrix score.	sequence	function
GCN4_motif	Oryza sativa	120	-	7	TGAGTCA	cis-regulatory element involved in endosperm expression

+ TC-rich repeats

Site Name	Organism	Position	Strand	Matrix score.	sequence	function
TC-rich repeats	Nicotiana tabacum	1393	-	9	GTTTTCTTAC	cis-acting element involved in defense and stress responsiveness

+ TGACG-motif

Site Name	Organism	Position	Strand	Matrix score.	sequence	function
TGACG-motif	Hordeum vulgare	1374	+	5	TGACG	cis-acting regulatory element involved in the MeJA-responsiveness

57 *A. coerulea* Aqcoe5G406900.1.p

+ ARE

Site Name	Organism	Position	Strand	Matrix score.	sequence	function
ARE	Zea mays	398	+	6	AAACCA	cis-acting regulatory element essential for the anaerobic induction

+ CGTCA-motif

Site Name	Organism	Position	Strand	Matrix score.	sequence	function
CGTCA-motif	Hordeum vulgare	561	-	5	CGTCA	cis-acting regulatory element involved in the MeJA-responsiveness

+ ERE

Site Name	Organism	Position	Strand	Matrix score.	sequence	function
ERE	Nicotiana glutinos	876	-	8	ATTTTAAA	

+ GARE-motif

Site Name	Organism	Position	Strand	Matrix score.	sequence	function
GARE-motif	Brassica oleracea	499	-	7	TCTGTTG	gibberellin-responsive element

+ GC-motif

Site Name	Organism	Position	Strand	Matrix score.	sequence	function
GC-motif	Zea mays	1095	+	6	CCCCCG	enhancer-like element involved in anoxic specific inducibility

+ TGA-element

Site Name	Organism	Position	Strand	Matrix score.	sequence	function
TGA-element	Brassica oleracea	1023	-	6	AACGAC	auxin-responsive element

+ TGACG-motif

Site Name	Organism	Position	Strand	Matrix score.	sequence	function
TGACG-motif	Hordeum vulgare	561	+	5	TGACG	cis-acting regulatory element involved in the MeJA-responsiveness

+ WUN-motif

Site Name	Organism	Position	Strand	Matrix score.	sequence	function
WUN-motif	Nicotiana glutinosa	1145	+	9	AAATTTCTT	

58 *A. coerulea* Aqcoe7G090200.1.p

+ ARE

Site Name	Organism	Position	Strand	Matrix score.	sequence	function
ARE	Zea mays	1248	+	6	AAACCA	cis-acting regulatory element essential for the anaerobic induction
ARE	Zea mays	419	+	6	AAACCA	cis-acting regulatory element essential for the anaerobic induction
ARE	Zea mays	116	-	6	AAACCA	cis-acting regulatory element essential for the anaerobic induction

+ ERE

Site Name	Organism	Position	Strand	Matrix score.	sequence	function
ERE	Nicotiana glutinos	268	-	8	ATTTTAAA	

+ GARE-motif

Site Name	Organism	Position	Strand	Matrix score.	sequence	function
GARE-motif	Brassica oleracea	1206	+	7	TCTGTTG	gibberellin-responsive element

+ LTR

Site Name	Organism	Position	Strand	Matrix score.	sequence	function
LTR	Hordeum vulgare	594	+	6	CCGAAA	cis-acting element involved in low-temperature responsiveness

+ MBS

Site Name	Organism	Position	Strand	Matrix score.	sequence	function
MBS	Arabidopsis thaliana	777	+	6	CAACTG	MYB binding site involved in drought-inducibility
MBS	Arabidopsis thaliana	1191	-	6	CAACTG	MYB binding site involved in drought-inducibility

+ P-box

Site Name	Organism	Position	Strand	Matrix score.	sequence	function
P-box	Petroselinum crispum	1475	-	12	CAACAAACCCCTT	gibberellin-responsive element and part of a light responsive element

+ TC-rich repeats

Site Name	Organism	Position	Strand	Matrix score.	sequence	function
TC-rich repeats	Nicotiana tabacum	547	-	9	GTTTTCTTAC	cis-acting element involved in defense and stress responsiveness

+ TCA-element

Site Name	Organism	Position	Strand	Matrix score.	sequence	function
TCA-element	Nicotiana tabacum	607	-	9	CCATCTTTTT	cis-acting element involved in salicylic acid responsiveness

+ TGA-element

Site Name	Organism	Position	Strand	Matrix score.	sequence	function
TGA-element	Brassica oleracea	623	+	6	AACGAC	auxin-responsive element

59 *A. trichopoda* evm_27.model.AmTr_v1.0_scaffold00078.136

None

60 *P. hallii* Pahal.C04345.1

+ ABRE

Site Name	Organism	Position	Strand	Matrix score.	sequence	function
ABRE	Arabidopsis thaliana	932	+	5	ACGTG	cis-acting element involved in the abscisic acid responsiveness
ABRE	Arabidopsis thaliana	925	-	5	ACGTG	cis-acting element involved in the abscisic acid responsiveness
ABRE	Arabidopsis thaliana	931	-	6	CACGTG	cis-acting element involved in the abscisic acid responsiveness

+ ERE

Site Name	Organism	Position	Strand	Matrix score.	sequence	function
ERE	Nicotiana glutinos	1093	+	8	ATTTTAAA	

+ LTR

Site Name	Organism	Position	Strand	Matrix score.	sequence	function
LTR	Hordeum vulgare	357	-	6	CCGAAA	cis-acting element involved in low-temperature responsiveness

61 *P. virgatum* Pavir.2NG607100.1.p

+ ABRE

Site Name	Organism	Position	Strand	Matrix score.	sequence	function
ABRE	Arabidopsis thaliana	540	-	5	ACGTG	cis-acting element involved in the abscisic acid responsiveness
ABRE	Arabidopsis thaliana	139	+	5	ACGTG	cis-acting element involved in the abscisic acid responsiveness

+ ARE

Site Name	Organism	Position	Strand	Matrix score.	sequence	function
ARE	Zea mays	360	+	6	AAACCA	cis-acting regulatory element essential for the anaerobic induction
ARE	Zea mays	175	-	6	AAACCA	cis-acting regulatory element essential for the anaerobic induction

+ LTR

Site Name	Organism	Position	Strand	Matrix score.	sequence	function
LTR	Hordeum vulgare	682	-	6	CCGAAA	cis-acting element involved in low-temperature responsiveness

+ WUN-motif

Site Name	Organism	Position	Strand	Matrix score.	sequence	function
WUN-motif	Brassica oleracea	289	+	9	AAATTTCCT	wound-responsive element

62 *P. virgatum* Pavir.2KG565100.1.p

\+ ABRE

Site Name	Organism	Position	Strand	Matrix score.	sequence	function
ABRE	Arabidopsis thaliana	655	+	5	ACGTG	cis-acting element involved in the abscisic acid responsiveness
ABRE	Arabidopsis thaliana	57	+	5	ACGTG	cis-acting element involved in the abscisic acid responsiveness
ABRE	Arabidopsis thaliana	783	+	7	AACCCGG	cis-acting element involved in the abscisic acid responsiveness

\+ ARE

Site Name	Organism	Position	Strand	Matrix score.	sequence	function
ARE	Zea mays	1416	+	6	AAACCA	cis-acting regulatory element essential for the anaerobic induction
ARE	Zea mays	595	+	6	AAACCA	cis-acting regulatory element essential for the anaerobic induction
ARE	Zea mays	73	-	6	AAACCA	cis-acting regulatory element essential for the anaerobic induction

\+ CGTCA-motif

Site Name	Organism	Position	Strand	Matrix score.	sequence	function
CGTCA-motif	Hordeum vulgare	1242	-	5	CGTCA	cis-acting regulatory element involved in the MeJA-responsiveness
CGTCA-motif	Hordeum vulgare	539	+	5	CGTCA	cis-acting regulatory element involved in the MeJA-responsiveness

+ ERE

Site Name	Organism	Position	Strand	Matrix score.	sequence	function
ERE	Nicotiana glutinos	1050	-	8	ATTTTAAA	

+ LTR

Site Name	Organism	Position	Strand	Matrix score.	sequence	function
LTR	Hordeum vulgare	853	-	6	CCGAAA	cis-acting element involved in low-temperature responsiveness

+ TGA-element

Site Name	Organism	Position	Strand	Matrix score.	sequence	function
TGA-element	Brassica oleracea	849	-	6	AACGAC	auxin-responsive element

+ TGACG-motif

Site Name	Organism	Position	Strand	Matrix score.	sequence	function
TGACG-motif	Hordeum vulgare	1242	+	5	TGACG	cis-acting regulatory element involved in the MeJA-responsiveness
TGACG-motif	Hordeum vulgare	539	-	5	TGACG	cis-acting regulatory element involved in the MeJA-responsiveness

63 *S. Italica* Seita.7G071900.1.p

\+ ABRE

Site Name	Organism	Position	Strand	Matrix score.	sequence	function
ABRE	Arabidopsis thaliana	958	+	5	ACGTG	cis-acting element involved in the abscisic acid responsiveness
ABRE	Arabidopsis thaliana	957	-	6	CACGTG	cis-acting element involved in the abscisic acid responsiveness
ABRE	Arabidopsis thaliana	394	+	5	ACGTG	cis-acting element involved in the abscisic acid responsiveness

\+ AuxRR-core

Site Name	Organism	Position	Strand	Matrix score.	sequence	function
AuxRR-core	Nicotiana tabacum	846	-	7	GGTCCAT	cis-acting regulatory element involved in auxin responsiveness

\+ CAT-box

Site Name	Organism	Position	Strand	Matrix score.	sequence	function
CAT-box	Arabidopsis thaliana	1244	+	6	GCCACT	cis-acting regulatory element related to meristem expression

\+ CGTCA-motif

Site Name	Organism	Position	Strand	Matrix score.	sequence	function
CGTCA-motif	Hordeum vulgare	498	-	5	CGTCA	cis-acting regulatory element involved in the MeJA-responsiveness

\+ GARE-motif

Site Name	Organism	Position	Strand	Matrix score.	sequence	function
GARE-motif	Brassica oleracea	527	+	7	TCTGTTG	gibberellin-responsive element

+ MBS

Site Name	Organism	Position	Strand	Matrix score.	sequence	function
MBS	Arabidopsis thaliana	104	-	6	CAACTG	MYB binding site involved in drought-inducibility

+ P-box

Site Name	Organism	Position	Strand	Matrix score.	sequence	function
P-box	Oryza sativa	429	-	7	CCTTTTG	gibberellin-responsive element

+ TC-rich repeats

Site Name	Organism	Position	Strand	Matrix score.	sequence	function
TC-rich repeats	Nicotiana tabacum	995	-	9	GTTTTCTTAC	cis-acting element involved in defense and stress responsiveness

+ TCA-element

Site Name	Organism	Position	Strand	Matrix score.	sequence	function
TCA-element	Nicotiana tabacum	840	-	9	CCATCTTTTT	cis-acting element involved in salicylic acid responsiveness
TCA-element	Nicotiana tabacum	468	+	9	CCATCTTTTT	cis-acting element involved in salicylic acid responsiveness
TCA-element	Nicotiana tabacum	123	+	9	CCATCTTTTT	cis-acting element involved in salicylic acid responsiveness

+ TGACG-motif

Site Name	Organism	Position	Strand	Matrix score.	sequence	function
TGACG-motif	Hordeum vulgare	498	+	5	TGACG	cis-acting regulatory element involved in the MeJA-responsiveness

64 *S. viridis* Sevir.7G077900.1.p

\+ ABRE

Site Name	Organism	Position	Strand	Matrix score.	sequence	function
ABRE	Arabidopsis thaliana	933	+	5	ACGTG	cis-acting element involved in the abscisic acid responsiveness
ABRE	Arabidopsis thaliana	932	-	6	CACGTG	cis-acting element involved in the abscisic acid responsiveness

\+ AuxRR-core

Site Name	Organism	Position	Strand	Matrix score.	sequence	function
AuxRR-core	Nicotiana tabacum	821	-	7	GGTCCAT	cis-acting regulatory element involved in auxin responsiveness

\+ CAT-box

Site Name	Organism	Position	Strand	Matrix score.	sequence	function
CAT-box	Arabidopsis thaliana	1219	+	6	GCCACT	cis-acting regulatory element related to meristem expression

\+ CGTCA-motif

Site Name	Organism	Position	Strand	Matrix score.	sequence	function
CGTCA-motif	Hordeum vulgare	470	-	5	CGTCA	cis-acting regulatory element involved in the MeJA-responsiveness

\+ GARE-motif

Site Name	Organism	Position	Strand	Matrix score.	sequence	function
GARE-motif	Brassica oleracea	499	+	7	TCTGTTG	gibberellin-responsive element

+ MBS

Site Name	Organism	Position	Strand	Matrix score.	sequence	function
MBS	Arabidopsis thaliana	76	-	6	CAACTG	MYB binding site involved in drought-inducibility

+ P-box

Site Name	Organism	Position	Strand	Matrix score.	sequence	function
P-box	Oryza sativa	401	-	7	CCTTTTG	gibberellin-responsive element

+ TC-rich repeats

Site Name	Organism	Position	Strand	Matrix score.	sequence	function
TC-rich repeats	Nicotiana tabacum	970	-	9	GTTTTCTTAC	cis-acting element involved in defense and stress responsiveness

+ TCA-element

Site Name	Organism	Position	Strand	Matrix score.	sequence	function
TCA-element	Nicotiana tabacum	95	+	9	CCATCTTTTT	cis-acting element involved in salicylic acid responsiveness
TCA-element	Nicotiana tabacum	815	-	9	CCATCTTTTT	cis-acting element involved in salicylic acid responsiveness
TCA-element	Nicotiana tabacum	440	+	9	CCATCTTTTT	cis-acting element involved in salicylic acid responsiveness

+ TGACG-motif

Site Name	Organism	Position	Strand	Matrix score.	sequence	function
TGACG-motif	Hordeum vulgare	470	+	5	TGACG	cis-acting regulatory element involved in the MeJA-responsiveness

65 *P. hallii* Pahal.G00914.1

\+ ABRE

Site Name	Organism	Position	Strand	Matrix score.	sequence	function
ABRE	Arabidopsis thaliana	1090	+	5	ACGTG	cis-acting element involved in the abscisic acid responsiveness
ABRE	Hordeum vulgare	970	-	9	GCAACGTGTC	cis-acting element involved in the abscisic acid responsiveness
ABRE	Arabidopsis thaliana	1089	-	6	CACGTG	cis-acting element involved in the abscisic acid responsiveness

\+ ARE

Site Name	Organism	Position	Strand	Matrix score.	sequence	function
ARE	Zea mays	1342	-	6	AAACCA	cis-acting regulatory element essential for the anaerobic induction

\+ CAT-box

Site Name	Organism	Position	Strand	Matrix score.	sequence	function
CAT-box	Arabidopsis thaliana	167	-	6	GCCACT	cis-acting regulatory element related to meristem expression

\+ CGTCA-motif

Site Name	Organism	Position	Strand	Matrix score.	sequence	function
CGTCA-motif	Hordeum vulgare	930	+	5	CGTCA	cis-acting regulatory element involved in the MeJA-responsiveness
CGTCA-motif	Hordeum vulgare	792	+	5	CGTCA	cis-acting regulatory element involved in the MeJA-responsiveness

+ GARE-motif

Site Name	Organism	Position	Strand	Matrix score.	sequence	function
GARE-motif	Brassica oleracea	737	+	7	TCTGTTG	gibberellin-responsive element

+ RY-element

Site Name	Organism	Position	Strand	Matrix score.	sequence	function
RY-element	Helianthus annuus	289	+	8	CATGCATG	cis-acting regulatory element involved in seed-specific regulation

+ TC-rich repeats

Site Name	Organism	Position	Strand	Matrix score.	sequence	function
TC-rich repeats	Nicotiana tabacum	480	-	9	GTTTTCTTAC	cis-acting element involved in defense and stress responsiveness

+ TCA-element

Site Name	Organism	Position	Strand	Matrix score.	sequence	function
TCA-element	Nicotiana tabacum	1335	-	9	CCATCTTTTT	cis-acting element involved in salicylic acid responsiveness

+ TGACG-motif

Site Name	Organism	Position	Strand	Matrix score.	sequence	function
TGACG-motif	Hordeum vulgare	792	-	5	TGACG	cis-acting regulatory element involved in the MeJA-responsiveness
TGACG-motif	Hordeum vulgare	930	-	5	TGACG	cis-acting regulatory element involved in the MeJA-responsiveness

66 *P. virgatum* Pavir.7KG359500.1.p

+ CAT-box

Site Name	Organism	Position	Strand	Matrix score.	sequence	function
CAT-box	Arabidopsis thaliana	430	-	6	GCCACT	cis-acting regulatory element related to meristem expression

+ CGTCA-motif

Site Name	Organism	Position	Strand	Matrix score.	sequence	function
CGTCA-motif	Hordeum vulgare	758	+	5	CGTCA	cis-acting regulatory element involved in the MeJA-responsiveness

+ GC-motif

Site Name	Organism	Position	Strand	Matrix score.	sequence	function
GC-motif	Zea mays	835	+	6	CCCCCG	enhancer-like element involved in anoxic specific inducibility
GC-motif	Zea mays	280	-	6	CCCCCG	enhancer-like element involved in anoxic specific inducibility
GC-motif	Zea mays	916	-	6	CCCCCG	enhancer-like element involved in anoxic specific inducibility

+ MBS

Site Name	Organism	Position	Strand	Matrix score.	sequence	function
MBS	Arabidopsis thaliana	809	-	6	CAACTG	MYB binding site involved in drought-inducibility

+ P-box

Site Name	Organism	Position	Strand	Matrix score.	sequence	function
P-box	Oryza sativa	626	-	7	CCTTTTG	gibberellin-responsive element

+ TGACG-motif

Site Name	Organism	Position	Strand	Matrix score.	sequence	function
TGACG-motif	Hordeum vulgare	758	-	5	TGACG	cis-acting regulatory element involved in the MeJA-responsiveness

+ TGACG-motif

Site Name	Organism	Position	Strand	Matrix score.	sequence	function
TGACG-motif	Hordeum vulgare	758	-	5	TGACG	cis-acting regulatory element involved in the MeJA-responsiveness

67 *Z. mays* Zm00008a037777_P01

+ ARE

Site Name	Organism	Position	Strand	Matrix score.	sequence	function
ARE	Zea mays	192	+	6	AAACCA	cis-acting regulatory element essential for the anaerobic induction
ARE	Zea mays	954	+	6	AAACCA	cis-acting regulatory element essential for the anaerobic induction

+ CGTCA-motif

Site Name	Organism	Position	Strand	Matrix score.	sequence	function
CGTCA-motif	Hordeum vulgare	1337	+	5	CGTCA	cis-acting regulatory element involved in the MeJA-responsiveness
CGTCA-motif	Hordeum vulgare	484	-	5	CGTCA	cis-acting regulatory element involved in the MeJA-responsiveness

+ GARE-motif

Site Name	Organism	Position	Strand	Matrix score.	sequence	function
GARE-motif	Brassica oleracea	834	+	7	TCTGTTG	gibberellin-responsive element
GARE-motif	Brassica oleracea	113	+	7	TCTGTTG	gibberellin-responsive element

+ MBS

Site Name	Organism	Position	Strand	Matrix score.	sequence	function
MBS	Arabidopsis thaliana	496	-	6	CAACTG	MYB binding site involved in drought-inducibility

+ TGACG-motif

Site Name	Organism	Position	Strand	Matrix score.	sequence	function
TGACG-motif	Hordeum vulgare	484	+	5	TGACG	cis-acting regulatory element involved in the MeJA-responsiveness
TGACG-motif	Hordeum vulgare	1337	-	5	TGACG	cis-acting regulatory element involved in the MeJA-responsiveness

+ WUN-motif

Site Name	Organism	Position	Strand	Matrix score.	sequence	function
WUN-motif	Brassica oleracea	975	+	9	AAATTTCCT	wound-responsive element

68 *Z. mays* Zm00008a007621_P01

+ ABRE

Site Name	Organism	Position	Strand	Matrix score.	sequence	function
ABRE	Arabidopsis thaliana	71	+	5	ACGTG	cis-acting element involved in the abscisic acid responsiveness
ABRE	Arabidopsis thaliana	1052	+	5	ACGTG	cis-acting element involved in the abscisic acid responsiveness
ABRE	Arabidopsis thaliana	1051	-	6	CACGTG	cis-acting element involved in the abscisic acid responsiveness
ABRE	Arabidopsis thaliana	999	+	5	ACGTG	cis-acting element involved in the abscisic acid responsiveness

+ CAT-box

Site Name	Organism	Position	Strand	Matrix score.	sequence	function
CAT-box	Arabidopsis thaliana	133	-	6	GCCACT	cis-acting regulatory element related to meristem expression

+ GARE-motif

Site Name	Organism	Position	Strand	Matrix score.	sequence	function
GARE-motif	Brassica oleracea	148	-	7	TCTGTTG	gibberellin-responsive element
GARE-motif	Brassica oleracea	522	-	7	TCTGTTG	gibberellin-responsive element

+ LTR

Site Name	Organism	Position	Strand	Matrix score.	sequence	function
LTR	Hordeum vulgare	1402	+	6	CCGAAA	cis-acting element involved in low-temperature responsiveness

+ TCA-element

Site Name	Organism	Position	Strand	Matrix score.	sequence	function
TCA-element	Nicotiana tabacum	706	-	9	CCATCTTTTT	cis-acting element involved in salicylic acid responsiveness

69 *S. bicolor* Sobic.001G409600.1.p

+ ABRE

Site Name	Organism	Position	Strand	Matrix score.	sequence	function
ABRE	Arabidopsis thaliana	627	-	5	ACGTG	cis-acting element involved in the abscisic acid responsiveness
ABRE	Arabidopsis thaliana	777	-	7	AACCCGG	cis-acting element involved in the abscisic acid responsiveness
ABRE	Arabidopsis thaliana	826	-	6	CACGTG	cis-acting element involved in the abscisic acid responsiveness
ABRE	Arabidopsis thaliana	827	+	5	ACGTG	cis-acting element involved in the abscisic acid responsiveness

+ AuxRR-core

Site Name	Organism	Position	Strand	Matrix score.	sequence	function
AuxRR-core	Nicotiana tabacum	1014	-	7	GGTCCAT	cis-acting regulatory element involved in auxin responsiveness

+ CAT-box

Site Name	Organism	Position	Strand	Matrix score.	sequence	function
CAT-box	Arabidopsis thaliana	77	+	6	GCCACT	cis-acting regulatory element related to meristem expression
CAT-box	Arabidopsis thaliana	661	+	6	GCCACT	cis-acting regulatory element related to meristem expression
CAT-box	Arabidopsis thaliana	435	+	6	GCCACT	cis-acting regulatory element related to meristem expression

+ CCGTCC-box

Site Name	Organism	Position	Strand	Matrix	sequence	function

				score.		
CCGTCC-box	Petroselinum hortense	837	-	6	CCGTCC	
CCGTCC-box	Petroselinum hortense	842	-	6	CCGTCC	

+ CGTCA-motif

Site Name	Organism	Position	Strand	Matrix score.	sequence	function
CGTCA-motif	Hordeum vulgare	1481	+	5	CGTCA	cis-acting regulatory element involved in the MeJA-responsiveness

+ GC-motif

Site Name	Organism	Position	Strand	Matrix score.	sequence	function
GC-motif	Zea mays	1389	+	6	CCCCCG	enhancer-like element involved in anoxic specific inducibility

+ TGACG-motif

Site Name	Organism	Position	Strand	Matrix score.	sequence	function
TGACG-motif	Hordeum vulgare	1481	-	5	TGACG	cis-acting regulatory element involved in the MeJA-responsiveness

70 *S. bicolor* Sobic.006G183800.1.p

+ ABRE

Site Name	Organism	Position	Strand	Matrix score.	sequence	function
ABRE	Arabidopsis thaliana	1048	-	6	CACGTG	cis-acting element involved in the abscisic acid responsiveness
ABRE	Arabidopsis thaliana	1103	+	5	ACGTG	cis-acting element involved in the abscisic acid responsiveness
ABRE	Arabidopsis thaliana	960	-	5	ACGTG	cis-acting element involved in the abscisic acid responsiveness
ABRE	Arabidopsis thaliana	1072	+	5	ACGTG	cis-acting element involved in the abscisic acid responsiveness
ABRE	Arabidopsis thaliana	1020	-	5	ACGTG	cis-acting element involved in the abscisic acid responsiveness
ABRE	Arabidopsis thaliana	1049	+	5	ACGTG	cis-acting element involved in the abscisic acid responsiveness
ABRE	Arabidopsis thaliana	924	-	5	ACGTG	cis-acting element involved in the abscisic acid responsiveness
ABRE	Arabidopsis thaliana	942	-	5	ACGTG	cis-acting element involved in the abscisic acid responsiveness

+ CCGTCC-box

Site Name	Organism	Position	Strand	Matrix score.	sequence	function
CCGTCC-box	Petroselinum hortense	966	-	6	CCGTCC	

+ TCA-element

Site Name	Organism	Position	Strand	Matrix score.	sequence	function
TCA-element	Nicotiana tabacum	368	+	9	CCATCTTTTT	cis-acting element involved in salicylic acid responsiveness

71 *O. Thomaeum* Oropetium_20150105_08811A

+ ABRE

Site Name	Organism	Position	Strand	Matrix score.	sequence	function
ABRE	Arabidopsis thaliana	1227	+	5	ACGTG	cis-acting element involved in the abscisic acid responsiveness

+ CGTCA-motif

Site Name	Organism	Position	Strand	Matrix score.	sequence	function
CGTCA-motif	Hordeum vulgare	1078	-	5	CGTCA	cis-acting regulatory element involved in the MeJA-responsiveness
CGTCA-motif	Hordeum vulgare	213	-	5	CGTCA	cis-acting regulatory element involved in the MeJA-responsiveness

+ ERE

Site Name	Organism	Position	Strand	Matrix score.	sequence	function
ERE	Nicotiana glutinos	749	+	8	ATTTTAAA	

+ LTR

Site Name	Organism	Position	Strand	Matrix score.	sequence	function
LTR	Hordeum vulgare	1287	+	6	CCGAAA	cis-acting element involved in low-temperature responsiveness

+ TCA-element

Site Name	Organism	Position	Strand	Matrix score.	sequence	function
TCA-element	Nicotiana tabacum	592	-	9	CCATCTTTTT	cis-acting element involved in salicylic acid responsiveness

+ TGA-element

Site Name	Organism	Position	Strand	Matrix score.	sequence	function
TGA-element	Brassica oleracea	1029	-	6	AACGAC	auxin-responsive element

+ TGACG-motif

Site Name	Organism	Position	Strand	Matrix score.	sequence	function
TGACG-motif	Hordeum vulgare	1078	+	5	TGACG	cis-acting regulatory element involved in the MeJA-responsiveness
TGACG-motif	Hordeum vulgare	213	+	5	TGACG	cis-acting regulatory element involved in the MeJA-responsiveness

72 *T. aestivum* Traes_2DL_6F03F05FA.4
None

73 *T. aestivum* Traes_2BL_F480B8D1F.2

+ ABRE

Site Name	Organism	Position	Strand	Matrix score.	sequence	function
ABRE	Arabidopsis thaliana	406	+	5	ACGTG	cis-acting element involved in the abscisic acid responsiveness
ABRE	Arabidopsis thaliana	405	-	6	CACGTG	cis-acting element involved in the abscisic acid responsiveness

+ CGTCA-motif

Site Name	Organism	Position	Strand	Matrix score.	sequence	function
CGTCA-motif	Hordeum vulgare	313	-	5	CGTCA	cis-acting regulatory element involved in the MeJA-responsiveness

+ ERE

Site Name	Organism	Position	Strand	Matrix score.	sequence	function
ERE	Nicotiana glutinos	9	-	8	ATTTTAAA	

+ LTR

Site Name	Organism	Position	Strand	Matrix score.	sequence	function
LTR	Hordeum vulgare	448	+	6	CCGAAA	cis-acting element involved in low-temperature responsiveness
LTR	Hordeum vulgare	394	-	6	CCGAAA	cis-acting element involved in low-temperature responsiveness

+ TGACG-motif

Site Name	Organism	Position	Strand	Matrix score.	sequence	function
TGACG-motif	Hordeum vulgare	313	+	5	TGACG	cis-acting regulatory element involved in the MeJA-responsiveness

74 *T. aestivum* Traes_2AL_3D6729692.1

None

75 ***B. stacei*** **Brast09G057100.1.p**

+ ABRE

Site Name	Organism	Position	Strand	Matrix score.	sequence	function
ABRE	Arabidopsis thaliana	489	+	5	ACGTG	cis-acting element involved in the abscisic acid responsiveness
ABRE	Arabidopsis thaliana	1421	-	5	ACGTG	cis-acting element involved in the abscisic acid responsiveness
ABRE	Arabidopsis thaliana	759	+	5	ACGTG	cis-acting element involved in the abscisic acid responsiveness

+ ARE

Site Name	Organism	Position	Strand	Matrix score.	sequence	function
ARE	Zea mays	529	-	6	AAACCA	cis-acting regulatory element essential for the anaerobic induction
ARE	Zea mays	382	+	6	AAACCA	cis-acting regulatory element essential for the anaerobic induction

+ CCGTCC-box

Site Name	Organism	Position	Strand	Matrix score.	sequence	function
CCGTCC-box	Petroselinum hortense	1458	-	6	CCGTCC	

+ CGTCA-motif

Site Name	Organism	Position	Strand	Matrix score.	sequence	function
CGTCA-motif	Hordeum vulgare	1272	+	5	CGTCA	cis-acting regulatory element involved in the MeJA-responsiveness
CGTCA-motif	Hordeum vulgare	870	-	5	CGTCA	cis-acting regulatory element involved in the MeJA-responsiveness

CGTCA-motif	Hordeum vulgare	1314	+	5	CGTCA	cis-acting regulatory element involved in the MeJA-responsiveness

+ LTR

Site Name	Organism	Position	Strand	Matrix score.	sequence	function
LTR	Hordeum vulgare	856	-	6	CCGAAA	cis-acting element involved in low-temperature responsiveness

+ TGACG-motif

Site Name	Organism	Position	Strand	Matrix score.	sequence	function
TGACG-motif	Hordeum vulgare	1272	-	5	TGACG	cis-acting regulatory element involved in the MeJA-responsiveness
TGACG-motif	Hordeum vulgare	1314	-	5	TGACG	cis-acting regulatory element involved in the MeJA-responsiveness
TGACG-motif	Hordeum vulgare	870	+	5	TGACG	cis-acting regulatory element involved in the MeJA-responsiveness

76 *B. distachyon* Bradi5g07850.3.p

+ ABRE

Site Name	Organism	Position	Strand	Matrix score.	sequence	function
ABRE	Arabidopsis thaliana	291	+	5	ACGTG	cis-acting element involved in the abscisic acid responsiveness
ABRE	Arabidopsis thaliana	1414	-	5	ACGTG	cis-acting element involved in the abscisic acid responsiveness

+ ARE

Site Name	Organism	Position	Strand	Matrix score.	sequence	function
ARE	Zea mays	1214	+	6	AAACCA	cis-acting regulatory element essential for the anaerobic induction

+ AuxRR-core

Site Name	Organism	Position	Strand	Matrix score.	sequence	function
AuxRR-core	Nicotiana tabacum	1117	+	7	GGTCCAT	cis-acting regulatory element involved in auxin responsiveness

+ CAT-box

Site Name	Organism	Position	Strand	Matrix score.	sequence	function
CAT-box	Arabidopsis thaliana	178	+	6	GCCACT	cis-acting regulatory element related to meristem expression
CAT-box	Arabidopsis thaliana	201	+	6	GCCACT	cis-acting regulatory element related to meristem expression

+ CCGTCC-box

Site Name	Organism	Position	Strand	Matrix score.	sequence	function
CCGTCC-box	Petroselinum hortense	739	-	6	CCGTCC	

+ CGTCA-motif

Site Name	Organism	Position	Strand	Matrix	sequence	function

				score.		
CGTCA-motif	Hordeum vulgare	1030	+	5	CGTCA	cis-acting regulatory element involved in the MeJA-responsiveness
CGTCA-motif	Hordeum vulgare	692	-	5	CGTCA	cis-acting regulatory element involved in the MeJA-responsiveness
CGTCA-motif	Hordeum vulgare	735	-	5	CGTCA	cis-acting regulatory element involved in the MeJA-responsiveness
CGTCA-motif	Hordeum vulgare	1097	+	5	CGTCA	cis-acting regulatory element involved in the MeJA-responsiveness

+ LTR

Site Name	Organism	Position	Strand	Matrix score.	sequence	function
LTR	Hordeum vulgare	1113	-	6	CCGAAA	cis-acting element involved in low-temperature responsiveness

+ TC-rich repeats

Site Name	Organism	Position	Strand	Matrix score.	sequence	function
TC-rich repeats	Nicotiana tabacum	873	+	9	GTTTTCTTAC	cis-acting element involved in defense and stress responsiveness

+ TGACG-motif

Site Name	Organism	Position	Strand	Matrix score.	sequence	function
TGACG-motif	Hordeum vulgare	1030	-	5	TGACG	cis-acting regulatory element involved in the MeJA-responsiveness
TGACG-motif	Hordeum vulgare	1097	-	5	TGACG	cis-acting regulatory element involved in the MeJA-responsiveness
TGACG-motif	Hordeum vulgare	692	+	5	TGACG	cis-acting regulatory element involved in the MeJA-responsiveness
TGACG-motif	Hordeum vulgare	735	+	5	TGACG	cis-acting regulatory element involved in the MeJA-responsiveness

77 *A. comosus* Aco019032.1

+ ABRE

Site Name	Organism	Position	Strand	Matrix score.	sequence	function
ABRE	Arabidopsis thaliana	1337	+	5	ACGTG	cis-acting element involved in the abscisic acid responsiveness
ABRE	Arabidopsis thaliana	1336	-	6	CACGTG	cis-acting element involved in the abscisic acid responsiveness

+ ARE

Site Name	Organism	Position	Strand	Matrix score.	sequence	function
ARE	Zea mays	353	-	6	AAACCA	cis-acting regulatory element essential for the anaerobic induction

+ CGTCA-motif

Site Name	Organism	Position	Strand	Matrix score.	sequence	function
CGTCA-motif	Hordeum vulgare	596	+	5	CGTCA	cis-acting regulatory element involved in the MeJA-responsiveness

+ ERE

Site Name	Organism	Position	Strand	Matrix score.	sequence	function
ERE	Nicotiana glutinos	257	+	8	ATTTTAAA	
ERE	Nicotiana glutinos	745	-	8	ATTTTAAA	
ERE	Nicotiana glutinos	420	-	8	ATTTTAAA	
ERE	Nicotiana glutinos	885	+	8	ATTTCATA	
ERE	Nicotiana glutinos	368	-	8	ATTTCATA	
ERE	Nicotiana glutinos	538	-	8	ATTTTAAA	

+ GC-motif

Site Name	Organism	Position	Strand	Matrix score.	sequence	function
GC-motif	Zea mays	1493	+	6	CCCCCG	enhancer-like element involved in anoxic specific inducibility

+ TGACG-motif

Site Name	Organism	Position	Strand	Matrix score.	sequence	function
TGACG-motif	Hordeum vulgare	596	-	5	TGACG	cis-acting regulatory element involved in the MeJA-responsiveness

78 *M. acuminata* GSMUA_Achr5P25100_001

+ ABRE

Site Name	Organism	Position	Strand	Matrix score.	sequence	function
ABRE	Arabidopsis thaliana	1188	-	5	ACGTG	cis-acting element involved in the abscisic acid responsiveness

+ ARE

Site Name	Organism	Position	Strand	Matrix score.	sequence	function
ARE	Zea mays	1327	+	6	AAACCA	cis-acting regulatory element essential for the anaerobic induction
ARE	Zea mays	1068	-	6	AAACCA	cis-acting regulatory element essential for the anaerobic induction
ARE	Zea mays	1133	+	6	AAACCA	cis-acting regulatory element essential for the anaerobic induction

+ CGTCA-motif

Site Name	Organism	Position	Strand	Matrix score.	sequence	function
CGTCA-motif	Hordeum vulgare	913	+	5	CGTCA	cis-acting regulatory element involved in the MeJA-responsiveness

+ MBS

Site Name	Organism	Position	Strand	Matrix score.	sequence	function
MBS	Arabidopsis thaliana	1433	+	6	CAACTG	MYB binding site involved in drought-inducibility

+ RY-element

Site Name	Organism	Position	Strand	Matrix score.	sequence	function
RY-element	Helianthus annuus	760	-	8	CATGCATG	cis-acting regulatory element involved in seed-specific regulation

+ TCA-element

Site Name	Organism	Position	Strand	Matrix score.	sequence	function
TCA-element	Brassica oleracea	210	+	9	TCAGAAGAGG	cis-acting element involved in salicylic acid responsiveness

+ TGACG-motif

Site Name	Organism	Position	Strand	Matrix score.	sequence	function
TGACG-motif	Hordeum vulgare	913	-	5	TGACG	cis-acting regulatory element involved in the MeJA-responsiveness

79 *S. Polyrhiza* Spipo17G0046100

+ ABRE

Site Name	Organism	Position	Strand	Matrix score.	sequence	function
ABRE	Arabidopsis thaliana	534	-	7	AACCCGG	cis-acting element involved in the abscisic acid responsiveness

+ AuxRR-core

Site Name	Organism	Position	Strand	Matrix score.	sequence	function
AuxRR-core	Nicotiana tabacum	973	-	7	GGTCCAT	cis-acting regulatory element involved in auxin responsiveness

+ CCGTCC-box

Site Name	Organism	Position	Strand	Matrix score.	sequence	function
CCGTCC-box	Petroselinum hortense	545	+	6	CCGTCC	
CCGTCC-box	Petroselinum hortense	465	+	6	CCGTCC	

+ CGTCA-motif

Site Name	Organism	Position	Strand	Matrix score.	sequence	function
CGTCA-motif	Hordeum vulgare	370	-	5	CGTCA	cis-acting regulatory element involved in the MeJA-responsiveness

+ GC-motif

Site Name	Organism	Position	Strand	Matrix score.	sequence	function
GC-motif	Zea mays	109	-	6	CCCCCG	enhancer-like element involved in anoxic specific inducibility

+ HD-Zip 1

Site Name	Organism	Position	Strand	Matrix score.	sequence	function
HD-Zip 1	Arabidopsis thaliana	87	+	8	CAAT(A/T)ATTG	element involved in differentiation of the palisade mesophyll cells

+ LTR

Site Name	Organism	Position	Strand	Matrix score.	sequence	function
LTR	Hordeum vulgare	1352	+	6	CCGAAA	cis-acting element involved in low-temperature responsiveness

80 *Z. Marina* Zosma157g00110.1

+ ARE

Site Name	Organism	Position	Strand	Matrix score.	sequence	function
ARE	Zea mays	74	-	6	AAACCA	cis-acting regulatory element essential for the anaerobic induction
ARE	Zea mays	1356	-	6	AAACCA	cis-acting regulatory element essential for the anaerobic induction
ARE	Zea mays	82	-	6	AAACCA	cis-acting regulatory element essential for the anaerobic induction
ARE	Zea mays	124	-	6	AAACCA	cis-acting regulatory element essential for the anaerobic induction
ARE	Zea mays	363	-	6	AAACCA	cis-acting regulatory element essential for the anaerobic induction

+ CCGTCC-box

Site Name	Organism	Position	Strand	Matrix score.	sequence	function
CCGTCC-box	Petroselinum hortense	1387	-	6	CCGTCC	

+ CGTCA-motif

Site Name	Organism	Position	Strand	Matrix score.	sequence	function
CGTCA-motif	Hordeum vulgare	1107	-	5	CGTCA	cis-acting regulatory element involved in the MeJA-responsiveness

+ GCN4_motif

Site Name	Organism	Position	Strand	Matrix score.	sequence	function
GCN4_motif	Oryza sativa	1119	+	7	TGAGTCA	cis-regulatory element involved in endosperm expression

\+ LTR

Site Name	Organism	Position	Strand	Matrix score.	sequence	function
LTR	Hordeum vulgare	253	-	6	CCGAAA	cis-acting element involved in low-temperature responsiveness
LTR	Hordeum vulgare	1359	-	6	CCGAAA	cis-acting element involved in low-temperature responsiveness

\+ MBS

Site Name	Organism	Position	Strand	Matrix score.	sequence	function
MBS	Arabidopsis thaliana	829	+	6	CAACTG	MYB binding site involved in drought-inducibility
MBS	Arabidopsis thaliana	726	+	6	CAACTG	MYB binding site involved in drought-inducibility

\+ TC-rich repeats

Site Name	Organism	Position	Strand	Matrix score.	sequence	function
TC-rich repeats	Nicotiana tabacum	233	+	9	GTTTTCTTAC	cis-acting element involved in defense and stress responsiveness

\+ TGA-element

Site Name	Organism	Position	Strand	Matrix score.	sequence	function
TGA-element	Brassica oleracea	1087	-	6	AACGAC	auxin-responsive element
TGA-element	Brassica oleracea	19	-	6	AACGAC	auxin-responsive element
TGA-element	Brassica oleracea	39	-	6	AACGAC	auxin-responsive element

\+ TGACG-motif

Site Name	Organism	Position	Strand	Matrix score.	sequence	function
TGACG-motif	Hordeum vulgare	1107	+	5	TGACG	cis-acting regulatory element involved in the MeJA-responsiveness

81 *P. patens* Pp3c7_10890V3.1.p

\+ ABRE

Site Name	Organism	Position	Strand	Matrix score.	sequence	function
ABRE	Arabidopsis thaliana	266	-	5	ACGTG	cis-acting element involved in the abscisic acid responsiveness
ABRE	Arabidopsis thaliana	1484	-	5	ACGTG	cis-acting element involved in the abscisic acid responsiveness

\+ ARE

Site Name	Organism	Position	Strand	Matrix score.	sequence	function
ARE	Zea mays	736	-	6	AAACCA	cis-acting regulatory element essential for the anaerobic induction
ARE	Zea mays	586	+	6	AAACCA	cis-acting regulatory element essential for the anaerobic induction
ARE	Zea mays	226	-	6	AAACCA	cis-acting regulatory element essential for the anaerobic induction

\+ AT-rich sequence

Site Name	Organism	Position	Strand	Matrix score.	sequence	function
AT-rich sequence	Pisum sativum	354	+	9	TAAAATACT	element for maximal elicitor-mediated activation (2copies)

\+ CCGTCC-box

Site Name	Organism	Position	Strand	Matrix score.	sequence	function
CCGTCC-box	Petroselinum hortense	1466	-	6	CCGTCC	

+ ERE

Site Name	Organism	Position	Strand	Matrix score.	sequence	function
ERE	Nicotiana glutinos	56	+	8	ATTTTAAA	
ERE	Nicotiana glutinos	752	-	8	ATTTTAAA	

+ TCA-element

Site Name	Organism	Position	Strand	Matrix score.	sequence	function
TCA-element	Nicotiana tabacum	1255	-	9	CCATCTTTTT	cis-acting element involved in salicylic acid responsiveness

+ WUN-motif

Site Name	Organism	Position	Strand	Matrix score.	sequence	function
WUN-motif	Nicotiana glutinosa	19	-	9	AAATTACTA	
WUN-motif	Nicotiana glutinosa	20	-	8	AAATTACT	

82 *P. patens* Pp3c11_24710V3.1.p

+ ABRE

Site Name	Organism	Position	Strand	Matrix score.	sequence	function
ABRE	Arabidopsis thaliana	955	-	6	CACGTG	cis-acting element involved in the abscisic acid responsiveness
ABRE	Arabidopsis thaliana	956	+	5	ACGTG	cis-acting element involved in the abscisic acid responsiveness

+ ARE

Site Name	Organism	Position	Strand	Matrix score.	sequence	function
ARE	Zea mays	155	-	6	AAACCA	cis-acting regulatory element essential for the anaerobic induction
ARE	Zea mays	959	-	6	AAACCA	cis-acting regulatory element essential for the anaerobic induction
ARE	Zea mays	1275	+	6	AAACCA	cis-acting regulatory element essential for the anaerobic induction
ARE	Zea mays	653	-	6	AAACCA	cis-acting regulatory element essential for the anaerobic induction

+ CCGTCC-box

Site Name	Organism	Position	Strand	Matrix score.	sequence	function
CCGTCC-box	Petroselinum hortense	1433	-	6	CCGTCC	

+ CGTCA-motif

Site Name	Organism	Position	Strand	Matrix score.	sequence	function
CGTCA-motif	Hordeum vulgare	440	+	5	CGTCA	cis-acting regulatory element involved in the MeJA-responsiveness

CGTCA-motif	Hordeum vulgare	875	-	5	CGTCA	cis-acting regulatory element involved in the MeJA-responsiveness
CGTCA-motif	Hordeum vulgare	509	+	5	CGTCA	cis-acting regulatory element involved in the MeJA-responsiveness

+ ERE

Site Name	Organism	Position	Strand	Matrix score.	sequence	function
ERE	Nicotiana glutinos	471	-	8	ATTTTAAA	

+ MBS

Site Name	Organism	Position	Strand	Matrix score.	sequence	function
MBS	Arabidopsis thaliana	129	-	6	CAACTG	MYB binding site involved in drought-inducibility

+ TC-rich repeats

Site Name	Organism	Position	Strand	Matrix score.	sequence	function
TC-rich repeats	Nicotiana tabacum	265	+	9	ATTCTCTAAC	cis-acting element involved in defense and stress responsiveness

+ TGACG-motif

Site Name	Organism	Position	Strand	Matrix score.	sequence	function
TGACG-motif	Hordeum vulgare	875	+	5	TGACG	cis-acting regulatory element involved in the MeJA-responsiveness
TGACG-motif	Hordeum vulgare	509	-	5	TGACG	cis-acting regulatory element involved in the MeJA-responsiveness
TGACG-motif	Hordeum vulgare	440	-	5	TGACG	cis-acting regulatory element involved in the MeJA-responsiveness

+ WUN-motif

Site Name	Organism	Position	Strand	Matrix score.	sequence	function
WUN-motif	Nicotiana glutinosa	539	+	8	AAATTACT	

83 *S. fallax* Sphfalx0015s0077.1.p

\+ ABRE

Site Name	Organism	Position	Strand	Matrix score.	sequence	function
ABRE	Arabidopsis thaliana	1246	-	5	ACGTG	cis-acting element involved in the abscisic acid responsiveness
ABRE	Arabidopsis thaliana	1202	-	5	ACGTG	cis-acting element involved in the abscisic acid responsiveness

\+ AuxRR-core

Site Name	Organism	Position	Strand	Matrix score.	sequence	function
AuxRR-core	Nicotiana tabacum	1346	-	7	GGTCCAT	cis-acting regulatory element involved in auxin responsiveness

\+ CAT-box

Site Name	Organism	Position	Strand	Matrix score.	sequence	function
CAT-box	Arabidopsis thaliana	1432	-	6	GCCACT	cis-acting regulatory element related to meristem expression

\+ ERE

Site Name	Organism	Position	Strand	Matrix score.	sequence	function
ERE	Nicotiana glutinos	279	-	8	ATTTCATA	

\+ WUN-motif

Site Name	Organism	Position	Strand	Matrix score.	sequence	function
WUN-motif	Brassica oleracea	926	+	9	AAATTTCCT	wound-responsive element

84 *S. fallax* Sphfalx0010s0197.1.p

+ ABRE

Site Name	Organism	Position	Strand	Matrix score.	sequence	function
ABRE	Arabidopsis thaliana	1299	+	5	ACGTG	cis-acting element involved in the abscisic acid responsiveness

+ ARE

Site Name	Organism	Position	Strand	Matrix score.	sequence	function
ARE	Zea mays	681	+	6	AAACCA	cis-acting regulatory element essential for the anaerobic induction
ARE	Zea mays	250	+	6	AAACCA	cis-acting regulatory element essential for the anaerobic induction
ARE	Zea mays	1396	+	6	AAACCA	cis-acting regulatory element essential for the anaerobic induction

+ CGTCA-motif

Site Name	Organism	Position	Strand	Matrix score.	sequence	function
CGTCA-motif	Hordeum vulgare	624	-	5	CGTCA	cis-acting regulatory element involved in the MeJA-responsiveness
CGTCA-motif	Hordeum vulgare	1358	+	5	CGTCA	cis-acting regulatory element involved in the MeJA-responsiveness
CGTCA-motif	Hordeum vulgare	1488	+	5	CGTCA	cis-acting regulatory element involved in the MeJA-responsiveness

+ GCN4_motif

Site Name	Organism	Position	Strand	Matrix score.	sequence	function
GCN4_motif	Oryza sativa	1035	-	7	TGAGTCA	cis-regulatory element involved in endosperm expression

+ MBS

Site Name	Organism	Position	Strand	Matrix score.	sequence	function
MBS	Arabidopsis thaliana	155	+	6	CAACTG	MYB binding site involved in drought-inducibility

+ P-box

Site Name	Organism	Position	Strand	Matrix score.	sequence	function
P-box	Petroselinum crispum	485	+	12	CAACAAACCCCTT	gibberellin-responsive element and part of a light responsive element
P-box	Oryza sativa	211	+	7	CCTTTTG	gibberellin-responsive element

+ TGACG-motif

Site Name	Organism	Position	Strand	Matrix score.	sequence	function
TGACG-motif	Hordeum vulgare	1488	-	5	TGACG	cis-acting regulatory element involved in the MeJA-responsiveness
TGACG-motif	Hordeum vulgare	1358	-	5	TGACG	cis-acting regulatory element involved in the MeJA-responsiveness
TGACG-motif	Hordeum vulgare	624	+	5	TGACG	cis-acting regulatory element involved in the MeJA-responsiveness

85 *M. polymorpha* Mapoly0009s0189.1.p

+ ABRE

Site Name	Organism	Position	Strand	Matrix score.	sequence	function
ABRE	Arabidopsis thaliana	1272	-	5	ACGTG	cis-acting element involved in the abscisic acid responsiveness

+ ARE

Site Name	Organism	Position	Strand	Matrix score.	sequence	function
ARE	Zea mays	816	+	6	AAACCA	cis-acting regulatory element essential for the anaerobic induction
ARE	Zea mays	575	+	6	AAACCA	cis-acting regulatory element essential for the anaerobic induction

+ CGTCA-motif

Site Name	Organism	Position	Strand	Matrix score.	sequence	function
CGTCA-motif	Hordeum vulgare	184	-	5	CGTCA	cis-acting regulatory element involved in the MeJA-responsiveness

+ GARE-motif

Site Name	Organism	Position	Strand	Matrix score.	sequence	function
GARE-motif	Brassica oleracea	97	+	7	TCTGTTG	gibberellin-responsive element

+ TGA-element

Site Name	Organism	Position	Strand	Matrix score.	sequence	function
TGA-element	Brassica oleracea	1280	-	6	AACGAC	auxin-responsive element
TGA-element	Brassica oleracea	973	-	6	AACGAC	auxin-responsive element

+ TGACG-motif

Site Name	Organism	Position	Strand	Matrix score.	sequence	function
TGACG-motif	Hordeum vulgare	184	+	5	TGACG	cis-acting regulatory element involved in the MeJA-responsiveness

86 *V. carteri* Vocar.0007s0345.1.p

+ ABRE

Site Name	Organism	Position	Strand	Matrix score.	sequence	function
ABRE	Arabidopsis thaliana	779	+	5	ACGTG	cis-acting element involved in the abscisic acid responsiveness
ABRE	Arabidopsis thaliana	756	+	5	ACGTG	cis-acting element involved in the abscisic acid responsiveness
ABRE	Arabidopsis thaliana	1425	+	5	ACGTG	cis-acting element involved in the abscisic acid responsiveness
ABRE	Arabidopsis thaliana	601	-	7	AACCCGG	cis-acting element involved in the abscisic acid responsiveness
ABRE	Hordeum vulgare	1422	+	9	CGTACGTGCA	cis-acting element involved in the abscisic acid responsiveness

+ ARE

Site Name	Organism	Position	Strand	Matrix score.	sequence	function
ARE	Zea mays	748	-	6	AAACCA	cis-acting regulatory element essential for the anaerobic induction

+ CAT-box

Site Name	Organism	Position	Strand	Matrix score.	sequence	function
CAT-box	Arabidopsis thaliana	534	+	6	GCCACT	cis-acting regulatory element related to meristem expression

+ CGTCA-motif

Site Name	Organism	Position	Strand	Matrix score.	sequence	function
CGTCA-motif	Hordeum vulgare	1402	-	5	CGTCA	cis-acting regulatory element involved in the MeJA-responsiveness
CGTCA-motif	Hordeum vulgare	1083	+	5	CGTCA	cis-acting regulatory element involved in the

						MeJA-responsiveness
CGTCA-motif	Hordeum vulgare	239	-	5	CGTCA	cis-acting regulatory element involved in the MeJA-responsiveness
CGTCA-motif	Hordeum vulgare	331	-	5	CGTCA	cis-acting regulatory element involved in the MeJA-responsiveness
CGTCA-motif	Hordeum vulgare	1223	+	5	CGTCA	cis-acting regulatory element involved in the MeJA-responsiveness

+ MBS

Site Name	Organism	Position	Strand	Matrix score.	sequence	function
MBS	Arabidopsis thaliana	1226	+	6	CAACTG	MYB binding site involved in drought-inducibility

+ TGACG-motif

Site Name	Organism	Position	Strand	Matrix score.	sequence	function
TGACG-motif	Hordeum vulgare	1402	+	5	TGACG	cis-acting regulatory element involved in the MeJA-responsiveness
TGACG-motif	Hordeum vulgare	331	+	5	TGACG	cis-acting regulatory element involved in the MeJA-responsiveness
TGACG-motif	Hordeum vulgare	1223	-	5	TGACG	cis-acting regulatory element involved in the MeJA-responsiveness
TGACG-motif	Hordeum vulgare	239	+	5	TGACG	cis-acting regulatory element involved in the MeJA-responsiveness
TGACG-motif	Hordeum vulgare	1083	-	5	TGACG	cis-acting regulatory element involved in the MeJA-responsiveness

87 *C. reinhardtii* Cre12.g553750.t1.1

+ ABRE

Site Name	Organism	Position	Strand	Matrix score.	sequence	function
ABRE	Arabidopsis thaliana	1219	-	5	ACGTG	cis-acting element involved in the abscisic acid responsiveness
ABRE	Arabidopsis thaliana	995	-	7	AACCCGG	cis-acting element involved in the abscisic acid responsiveness

+ CAT-box

Site Name	Organism	Position	Strand	Matrix score.	sequence	function
CAT-box	Arabidopsis thaliana	1256	-	6	GCCACT	cis-acting regulatory element related to meristem expression
CAT-box	Arabidopsis thaliana	701	-	6	GCCACT	cis-acting regulatory element related to meristem expression

+ CCGTCC-box

Site Name	Organism	Position	Strand	Matrix score.	sequence	function
CCGTCC-box	Petroselinum hortense	988	+	6	CCGTCC	
CCGTCC-box	Petroselinum hortense	897	-	6	CCGTCC	
CCGTCC-box	Petroselinum hortense	50	-	6	CCGTCC	

+ CGTCA-motif

Site Name	Organism	Position	Strand	Matrix score.	sequence	function
CGTCA-motif	Hordeum vulgare	1019	+	5	CGTCA	cis-acting regulatory element involved in the MeJA-responsiveness
CGTCA-motif	Hordeum vulgare	642	-	5	CGTCA	cis-acting regulatory element involved in the MeJA-responsiveness
CGTCA-motif	Hordeum vulgare	982	+	5	CGTCA	cis-acting regulatory element involved in the MeJA-responsiveness

+ GC-motif

Site Name	Organism	Position	Strand	Matrix score.	sequence	function
GC-motif	Zea mays	308	-	6	CCCCCG	enhancer-like element involved in anoxic specific inducibility

+ TGA-element

Site Name	Organism	Position	Strand	Matrix score.	sequence	function
TGA-element	Brassica oleracea	264	-	6	AACGAC	auxin-responsive element

+ TGACG-motif

Site Name	Organism	Position	Strand	Matrix score.	sequence	function
TGACG-motif	Hordeum vulgare	1019	-	5	TGACG	cis-acting regulatory element involved in the MeJA-responsiveness
TGACG-motif	Hordeum vulgare	982	-	5	TGACG	cis-acting regulatory element involved in the MeJA-responsiveness
TGACG-motif	Hordeum vulgare	642	+	5	TGACG	cis-acting regulatory element involved in the MeJA-responsiveness

88 *M. pusilla* 145219

+ AuxRR-core

Site Name	Organism	Position	Strand	Matrix score.	sequence	function
AuxRR-core	Nicotiana tabacum	148	+	7	GGTCCAT	cis-acting regulatory element involved in auxin responsiveness

+ CCGTCC-box

Site Name	Organism	Position	Strand	Matrix score.	sequence	function
CCGTCC-box	Petroselinum hortense	872	-	6	CCGTCC	

+ CGTCA-motif

Site Name	Organism	Position	Strand	Matrix score.	sequence	function
CGTCA-motif	Hordeum vulgare	1464	+	5	CGTCA	cis-acting regulatory element involved in the MeJA-responsiveness
CGTCA-motif	Hordeum vulgare	838	-	5	CGTCA	cis-acting regulatory element involved in the MeJA-responsiveness

+ GC-motif

Site Name	Organism	Position	Strand	Matrix score.	sequence	function
GC-motif	Zea mays	1318	+	6	CCCCCG	enhancer-like element involved in anoxic specific inducibility
GC-motif	Zea mays	817	-	6	CCCCCG	enhancer-like element involved in anoxic specific inducibility
GC-motif	Zea mays	583	+	6	CCCCCG	enhancer-like element involved in anoxic specific inducibility
GC-motif	Zea mays	269	+	6	CCCCCG	enhancer-like element involved in anoxic specific inducibility
GC-motif	Zea mays	904	-	6	CCCCCG	enhancer-like element involved in anoxic specific inducibility

+ LTR

Site Name	Organism	Position	Strand	Matrix	sequence	function

				score.		
LTR	Hordeum vulgare	1408	+	6	CCGAAA	cis-acting element involved in low-temperature responsiveness

+ OCT

Site Name	Organism	Position	Strand	Matrix score.	sequence	function
OCT	Zea mays	709	-	8	CGCGGATC	
OCT	Zea mays	1200	-	8	CGCGGATC	
OCT	Zea mays	454	-	8	CGCGGATC	

+ TGACG-motif

Site Name	Organism	Position	Strand	Matrix score.	sequence	function
TGACG-motif	Hordeum vulgare	1464	-	5	TGACG	cis-acting regulatory element involved in the MeJA-responsiveness
TGACG-motif	Hordeum vulgare	838	+	5	TGACG	cis-acting regulatory element involved in the MeJA-responsiveness

89 *V. carteri* Vocar.0008s0294.1.p

+ ABRE

Site Name	Organism	Position	Strand	Matrix score.	sequence	function
ABRE	Arabidopsis thaliana	102	+	5	ACGTG	cis-acting element involved in the abscisic acid responsiveness
ABRE	Arabidopsis thaliana	30	-	5	ACGTG	cis-acting element involved in the abscisic acid responsiveness
ABRE	Arabidopsis thaliana	1482	+	5	ACGTG	cis-acting element involved in the abscisic acid responsiveness
ABRE	Arabidopsis thaliana	679	+	6	CACGTG	cis-acting element involved in the abscisic acid responsiveness
ABRE	Arabidopsis thaliana	680	+	5	ACGTG	cis-acting element involved in the abscisic acid responsiveness
ABRE	Hordeum vulgare	1479	+	9	GCAACGTGTC	cis-acting element involved in the abscisic acid responsiveness

+ CAT-box

Site Name	Organism	Position	Strand	Matrix score.	sequence	function
CAT-box	Arabidopsis thaliana	1409	-	6	GCCACT	cis-acting regulatory element related to meristem expression

+ CGTCA-motif

Site Name	Organism	Position	Strand	Matrix score.	sequence	function
CGTCA-motif	Hordeum vulgare	1114	-	5	CGTCA	cis-acting regulatory element involved in the MeJA-responsiveness
CGTCA-motif	Hordeum vulgare	1080	+	5	CGTCA	cis-acting regulatory element involved in the MeJA-responsiveness
CGTCA-motif	Hordeum vulgare	950	-	5	CGTCA	cis-acting regulatory element involved in the MeJA-responsiveness

CGTCA-motif	Hordeum vulgare	1199	-	5	CGTCA	cis-acting regulatory element involved in the MeJA-responsiveness

+ LTR

Site Name	Organism	Position	Strand	Matrix score.	sequence	function
LTR	Hordeum vulgare	1397	-	6	CCGAAA	cis-acting element involved in low-temperature responsiveness
LTR	Hordeum vulgare	545	-	6	CCGAAA	cis-acting element involved in low-temperature responsiveness
LTR	Hordeum vulgare	79	+	6	CCGAAA	cis-acting element involved in low-temperature responsiveness

+ MBS

Site Name	Organism	Position	Strand	Matrix score.	sequence	function
MBS	Arabidopsis thaliana	259	-	6	CAACTG	MYB binding site involved in drought-inducibility

+ TGACG-motif

Site Name	Organism	Position	Strand	Matrix score.	sequence	function
TGACG-motif	Hordeum vulgare	1199	+	5	TGACG	cis-acting regulatory element involved in the MeJA-responsiveness
TGACG-motif	Hordeum vulgare	950	+	5	TGACG	cis-acting regulatory element involved in the MeJA-responsiveness
TGACG-motif	Hordeum vulgare	1080	-	5	TGACG	cis-acting regulatory element involved in the MeJA-responsiveness
TGACG-motif	Hordeum vulgare	1114	+	5	TGACG	cis-acting regulatory element involved in the MeJA-responsiveness

90 *C. reinhardtii* Cre09.g386731.t1.1

+ ABRE

Site Name	Organism	Position	Strand	Matrix score.	sequence	function
ABRE	Arabidopsis thaliana	352	+	5	ACGTG	cis-acting element involved in the abscisic acid responsiveness
ABRE	Arabidopsis thaliana	351	+	6	CACGTG	cis-acting element involved in the abscisic acid responsiveness

+ CAT-box

Site Name	Organism	Position	Strand	Matrix score.	sequence	function
CAT-box	Arabidopsis thaliana	273	-	6	GCCACT	cis-acting regulatory element related to meristem expression
CAT-box	Arabidopsis thaliana	202	+	6	GCCACT	cis-acting regulatory element related to meristem expression

+ CGTCA-motif

Site Name	Organism	Position	Strand	Matrix score.	sequence	function
CGTCA-motif	Hordeum vulgare	512	+	5	CGTCA	cis-acting regulatory element involved in the MeJA-responsiveness
CGTCA-motif	Hordeum vulgare	294	-	5	CGTCA	cis-acting regulatory element involved in the MeJA-responsiveness
CGTCA-motif	Hordeum vulgare	50	+	5	CGTCA	cis-acting regulatory element involved in the MeJA-responsiveness

+ GC-motif

Site Name	Organism	Position	Strand	Matrix score.	sequence	function
GC-motif	Oryza sativa	109	+	9	CGCCGCGCA	?

+ LTR

Site Name	Organism	Position	Strand	Matrix score.	sequence	function
LTR	Hordeum vulgare	1180	-	6	CCGAAA	cis-acting element involved in low-temperature responsiveness
LTR	Hordeum vulgare	852	+	6	CCGAAA	cis-acting element involved in low-temperature responsiveness

+ MBS

Site Name	Organism	Position	Strand	Matrix score.	sequence	function
MBS	Arabidopsis thaliana	899	-	6	CAACTG	MYB binding site involved in drought-inducibility
MBS	Arabidopsis thaliana	136	+	6	CAACTG	MYB binding site involved in drought-inducibility
MBS	Arabidopsis thaliana	1354	+	6	CAACTG	MYB binding site involved in drought-inducibility

+ TGACG-motif

Site Name	Organism	Position	Strand	Matrix score.	sequence	function
TGACG-motif	Hordeum vulgare	512	-	5	TGACG	cis-acting regulatory element involved in the MeJA-responsiveness
TGACG-motif	Hordeum vulgare	50	-	5	TGACG	cis-acting regulatory element involved in the MeJA-responsiveness
TGACG-motif	Hordeum vulgare	294	+	5	TGACG	cis-acting regulatory element involved in the MeJA-responsiveness

91 *M. pusilla* 50949

+ ABRE

Site Name	Organism	Position	Strand	Matrix score.	sequence	function
ABRE	Arabidopsis thaliana	758	+	5	ACGTG	cis-acting element involved in the abscisic acid responsiveness
ABRE	Arabidopsis thaliana	985	+	5	ACGTG	cis-acting element involved in the abscisic acid responsiveness
ABRE	Hordeum vulgare	647	-	9	CGTACGTGCA	cis-acting element involved in the abscisic acid responsiveness
ABRE	Oryza sativa	1390	+	9	GCCGCGTGGC	cis-acting element involved in the abscisic acid responsiveness
ABRE	Hordeum vulgare	651	+	9	CGCACGTGTC	cis-acting element involved in the abscisic acid responsiveness

+ ARE

Site Name	Organism	Position	Strand	Matrix score.	sequence	function
ARE	Zea mays	261	+	6	AAACCA	cis-acting regulatory element essential for the anaerobic induction

+ CCGTCC-box

Site Name	Organism	Position	Strand	Matrix score.	sequence	function
CCGTCC-box	Petroselinum hortense	1170	-	6	CCGTCC	
CCGTCC-box	Petroselinum hortense	857	-	6	CCGTCC	
CCGTCC-box	Petroselinum hortense	425	+	6	CCGTCC	

+ CGTCA-motif

Site Name	Organism	Position	Strand	Matrix score.	sequence	function
CGTCA-motif	Hordeum vulgare	831	+	5	CGTCA	cis-acting regulatory element involved in the MeJA-responsiveness
CGTCA-motif	Hordeum vulgare	810	+	5	CGTCA	cis-acting regulatory element involved in the MeJA-responsiveness
CGTCA-motif	Hordeum vulgare	322	+	5	CGTCA	cis-acting regulatory element involved in the MeJA-responsiveness
CGTCA-motif	Hordeum vulgare	393	-	5	CGTCA	cis-acting regulatory element involved in the MeJA-responsiveness

CGTCA-motif	Hordeum vulgare	45	-	5	CGTCA	cis-acting regulatory element involved in the MeJA-responsiveness

+ LTR

Site Name	Organism	Position	Strand	Matrix score.	sequence	function
LTR	Hordeum vulgare	131	+	6	CCGAAA	cis-acting element involved in low-temperature responsiveness

+ OCT

Site Name	Organism	Position	Strand	Matrix score.	sequence	function
OCT	Zea mays	1456	-	8	CGCGGATC	

+ TGA-element

Site Name	Organism	Position	Strand	Matrix score.	sequence	function
TGA-element	Brassica oleracea	1242	-	6	AACGAC	auxin-responsive element
TGA-element	Brassica oleracea	1	+	6	AACGAC	auxin-responsive element
TGA-element	Brassica oleracea	791	-	6	AACGAC	auxin-responsive element
TGA-element	Brassica oleracea	473	+	6	AACGAC	auxin-responsive element
TGA-element	Brassica oleracea	197	+	6	AACGAC	auxin-responsive element

+ TGACG-motif

Site Name	Organism	Position	Strand	Matrix score.	sequence	function
TGACG-motif	Hordeum vulgare	810	-	5	TGACG	cis-acting regulatory element involved in the MeJA-responsiveness
TGACG-motif	Hordeum vulgare	831	-	5	TGACG	cis-acting regulatory element involved in the MeJA-responsiveness
TGACG-motif	Hordeum vulgare	393	+	5	TGACG	cis-acting regulatory element involved in the MeJA-responsiveness
TGACG-motif	Hordeum vulgare	45	+	5	TGACG	cis-acting regulatory element involved in the MeJA-responsiveness
TGACG-motif	Hordeum vulgare	322	-	5	TGACG	cis-acting regulatory element involved in the MeJA-responsiveness

92 *M.* sp. 62857

+ ABRE

Site Name	Organism	Position	Strand	Matrix score.	sequence	function
ABRE	Arabidopsis thaliana	1187	+	5	ACGTG	cis-acting element involved in the abscisic acid responsiveness
ABRE	Arabidopsis thaliana	920	+	5	ACGTG	cis-acting element involved in the abscisic acid responsiveness
ABRE	Arabidopsis thaliana	323	-	5	ACGTG	cis-acting element involved in the abscisic acid responsiveness
ABRE	Arabidopsis thaliana	95	+	5	ACGTG	cis-acting element involved in the abscisic acid responsiveness
ABRE	Arabidopsis thaliana	94	+	6	CACGTG	cis-acting element involved in the abscisic acid responsiveness

+ CCGTCC-box

Site Name	Organism	Position	Strand	Matrix score.	sequence	function
CCGTCC-box	Petroselinum hortense	1026	-	6	CCGTCC	
CCGTCC-box	Petroselinum hortense	275	+	6	CCGTCC	
CCGTCC-box	Petroselinum hortense	746	+	6	CCGTCC	

+ CGTCA-motif

Site Name	Organism	Position	Strand	Matrix score.	sequence	function
CGTCA-motif	Hordeum vulgare	1445	-	5	CGTCA	cis-acting regulatory element involved in the MeJA-responsiveness
CGTCA-motif	Hordeum vulgare	1185	-	5	CGTCA	cis-acting regulatory element involved in the MeJA-responsiveness
CGTCA-motif	Hordeum vulgare	1166	-	5	CGTCA	cis-acting regulatory element involved in the MeJA-responsiveness
CGTCA-motif	Hordeum vulgare	303	-	5	CGTCA	cis-acting regulatory element involved in the MeJA-responsiveness
CGTCA-motif	Hordeum vulgare	510	-	5	CGTCA	cis-acting regulatory element involved in the MeJA-responsiveness
CGTCA-motif	Hordeum vulgare	780	+	5	CGTCA	cis-acting regulatory element involved in the MeJA-responsiveness

CGTCA-motif	Hordeum vulgare	558	-	5	CGTCA	cis-acting regulatory element involved in the MeJA-responsiveness

\+ GC-motif

Site Name	Organism	Position	Strand	Matrix score.	sequence	function
GC-motif	Zea mays	117	+	6	CCCCCG	enhancer-like element involved in anoxic specific inducibility
GC-motif	Zea mays	1209	-	6	CCCCCG	enhancer-like element involved in anoxic specific inducibility

\+ MBS

Site Name	Organism	Position	Strand	Matrix score.	sequence	function
MBS	Arabidopsis thaliana	992	-	6	CAACTG	MYB binding site involved in drought-inducibility

\+ TATC-box

Site Name	Organism	Position	Strand	Matrix score.	sequence	function
TATC-box	Oryza sativa	1395	+	7	TATCCCA	cis-acting element involved in gibberellin-responsiveness

\+ TGACG-motif

Site Name	Organism	Position	Strand	Matrix score.	sequence	function
TGACG-motif	Hordeum vulgare	780	-	5	TGACG	cis-acting regulatory element involved in the MeJA-responsiveness
TGACG-motif	Hordeum vulgare	510	+	5	TGACG	cis-acting regulatory element involved in the MeJA-responsiveness
TGACG-motif	Hordeum vulgare	558	+	5	TGACG	cis-acting regulatory element involved in the MeJA-responsiveness
TGACG-motif	Hordeum vulgare	303	+	5	TGACG	cis-acting regulatory element involved in the MeJA-responsiveness
TGACG-motif	Hordeum vulgare	1445	+	5	TGACG	cis-acting regulatory element involved in the MeJA-responsiveness
TGACG-motif	Hordeum vulgare	1185	+	5	TGACG	cis-acting regulatory element involved in the MeJA-responsiveness
TGACG-motif	Hordeum vulgare	1166	+	5	TGACG	cis-acting regulatory element involved in the MeJA-responsiveness
