# Supplementary material for: Systematic characterization of the branch point binding protein, splicing factor 1, gene family in plant development and stress responses
Source: BMC Plant Biol. 2020 Aug 18;20:379. doi: 10.1186/s12870-020-02570-6 (PMC7433366; doi:10.1186/s12870-020-02570-6)
Supplement: Supplementary file 7 — Additional file 7: Table S6. Primers used for RT-PCR and qPCR analysis. [file 12870_2020_2570_MOESM7_ESM.docx]

**Table S6 Primers used for RT-PCR and qPCR analysis.**

| **Gene name** | **Forward primer** (5'-3') | **Reverse primer** (5'-3') | **Purpose** |
| --- | --- | --- | --- |
| AT5G51300.1 | CCCAAACCTCCAGTACAGCC | TCCAAGAAACATCACACCTCTC | qPCR |
|  | CTGCACCGGCCTCGTCTA | ATCTCCAAGAAACATCACACCTCTC | RT-PCR |
| AT5G51300.2 | ACTGATGTGTTTGTGAGCAA | TTCGAACATCCAACTCCCGG | qPCR |
|  | TCGAATCAACTGGCTTTCAACA | TTCGAACATCCAACTCCCGG | RT-PCR |
| AT5G51300.3 | GTACCCTGGGCTCCCAAACCT | GCCTGAATCTCTCTCCACTA | qPCR |
|  | CCGGCCTCGTCTACTGAT | GCCTGAATCTCTCTCCACTA | RT-PCR |
| AtActin1 | CCCGCTATGTATGTCGC | AAGGTCAAGACGGAGGAT | qPCR |
| AtActin2 | TGTGCCAATCTACGAGGGT | GCTGGTCTTTGAGGTTTCC | RT-PCR |
| Potri.001G126400.1 | GGCAGAGATGAAATGAGGCTCT | TGTGAGTCCCAAACTTCCAACA | qPCR |
|  | GCAGTCTGGTGCTCAACCT | TGTGAGTCCCAAACTTCCAACA | RT-PCR |
| Potri.001G126400.2 | AACTTGACTGTGCCACCTCC | CTCGCTAGCCCAAATCTCTCA | qPCR |
|  | CAACTCAGACTGTGACGCCT | CTCGCTAGCCCAAATCTCTCA | RT-PCR |
| 18S rRNA (Pt) | CTCTGCCCGTTGCTCTGATGAT | CCTTGGATGTGGTAGCCGTTTCT | qPCR and RT-PCR |
